# Sea-level contribution from Thwaites Glacier doubles in an observation-constrained ice-sheet model

**DOI:** 10.1038/s41467-026-77976-1

**Published:** 2026-10-12

**Authors:** Mattia Poinelli, Eric Rignot, Hélène Seroussi, Joshua Cuzzone, Tyler Pelle, Eric Larour

**Affiliations:** 1 Joint Institute for Regional Earth System Science & Engineering, University of California, Los Angeles, CA USA; 2 Jet Propulsion Laboratory, California Institute of Technology, Pasadena, CA USA; 3 University of California, Irvine, CA USA; 4 Thayer School of Engineering, Dartmouth College, Hanover, NH USA; 5 Scripps Institution of Oceanography, University of California, San Diego, La Jolla, CA USA

**Keywords:** Cryospheric science, Projection and prediction

## Abstract

Thwaites Glacier is a major contributor to Antarctic mass loss and a key source of uncertainty in future sea-level rise. Ocean-driven melting at its grounding line drives rapid retreat, yet most models treat the transition between grounded and floating ice as a sharp boundary, neglecting kilometer-scale seawater intrusions that develop as the grounding line migrates and forms a grounding zone. Here, we show that actively-melting grounding zones are required to reproduce nearly three decades of observed retreat, thinning, and acceleration of Thwaites Glacier in a calibrated ice-sheet model, with successful reconstruction occurring only when grounding zones are represented as 2-5 km wide regions. Extending this model forward, we show that grounding-zone processes govern glacier’s detachment from stabilizing seafloor ridges, enabling unpinning as early as 2038 and producing up to 30 ± 6 mm of global mean sea-level rise by 2100 - approximately double previous projections even without additional climate warming.

## Abstract (web summary)

Grounding zones, where seawater intrudes under grounded ice and causes melting, are largely absent from ice-sheet models. Adding them improves Thwaites Glacier hindcasts 4-fold and doubles sea-level projections with respect to past estimates.

## Introduction

**Fig. 1:**
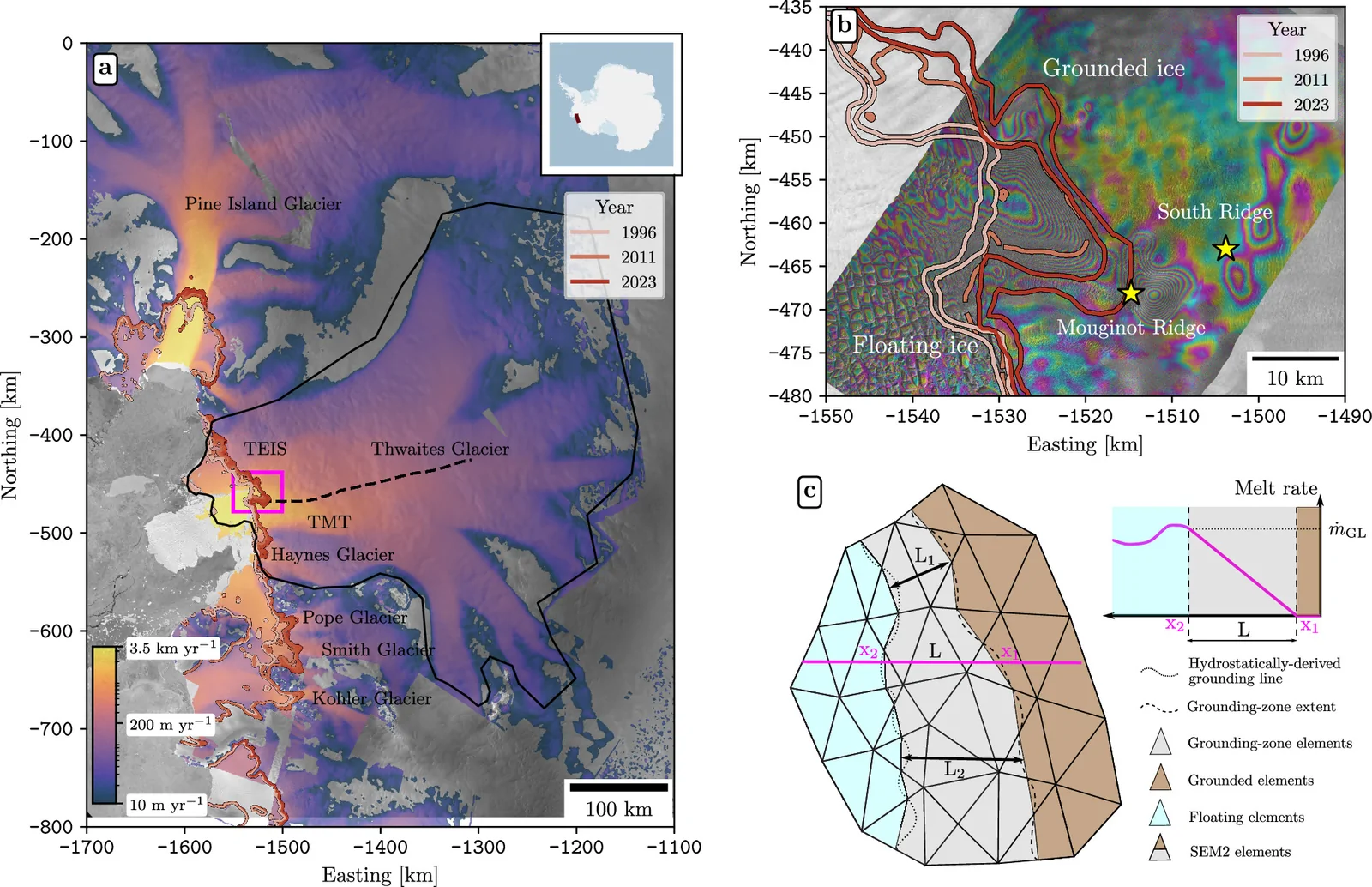
Thwaites Glacier underwent rapid changes in the past few decades. **a** Ice-sheet speed derived from ERS-1/2 and RADARSAT-1 Synthetic Aperture Radar (SAR) data between 1996 and 2001^34^, overlaying the RADARSAT-derived SAR mosaic. Red lines are grounding-line locations from 1996 to 2023^36^. Dashed line is a flow-line along the fastest flowing sector of Thwaites. Magenta box is the area of observed fastest retreat. TMT Thwaites Main trunk, TEIS Thwaites East Ice Shelf. **b** Differential, interferometric SAR fringes from ICEYE acquired in 2023^23^, in the magenta box of panel (**a**). Red lines are the observed grounding zone extent from 1996 to 2023. **c** Grounding zone parameterization in ISSM, where L is grounding-zone length, ${\dot{m}}_{GL}$ is melt rate the entrance of the grounding zone (*X*_2_), blue and brown elements are floating and grounded ice elements. Gray elements are actively-melting grounding zone elements. SEM2 are elements with a sub-element-melt parameterization^61^.

The Amundsen Sea Embayment (ASE) sector of West Antarctica accounts for ~one-third of the mass loss from the Antarctic Ice Sheet^1^, underscoring its outsized influence on future sea-level rise. In recent decades, Thwaites Glacier, a major component of ASE (Fig. 1a) with a sea-level equivalent of^2^ 65 cm, has experienced rapid retreat, thinning and acceleration^3^. These changes are driven largely by processes operating at the grounding line, the transition where grounded ice becomes afloat on the ocean surface in the form of ice shelves. There, intrusions of warm modified Circumpolar Deep Water (mCDW) drive intense ocean-induced melting^4^ that removes grounded ice and destabilizes the glacier. Since at least the mid-1990s^5^, mCDW has eroded the floating ice base of Thwaites, leading to progressive ungrounding from the seafloor, driving approximately 25 km of grounding-line retreat in the glacier’s western, fastest-flowing sector (Fig. 1b).

Previous studies^6–8^ have demonstrated that retreat of the grounding line into retrograde seafloor can trigger unstable ice loss and have explored the sensitivity of Thwaites to basal melt forcing. Despite advances in numerical simulations, ice-sheet models generally struggle to simultaneously reproduce historical glacier retreat, ice-flow acceleration and thinning^9^. Coupled ice-ocean simulations improve agreement relative to uncoupled models with idealized ocean forcing^10^, but substantial mismatches remain, particularly in how retreat is partitioned between the fast-flowing, deep main trunk and the slower, shallower eastern ice shelf, which are respectively underestimated and overestimated^10,11^. These discrepancies likely point to deficiencies in the representation of model boundaries^12^, including uncertainties at the seafloor^2^, at the ice base^6,13^ and at the grounding line^14^. As a result, Thwaites Glacier remains a major source of uncertainty in estimating the short- and long-term contribution of Antarctica to the rise of global sea-level^15^.

An approach to reducing uncertainty in sea-level projections is to calibrate ice-sheet models against available observations^9,16–20^, which now span more than four decades^1^. This strategy is based on the premise that recent trends strongly influence near-future glacier evolution^21,22^ and that the reliability of projections should be measured by how well models match the modern observation record. High-resolution satellite observations from the US-Finnish Synthetic Aperture Radar (SAR) constellation ICEYE have recently revealed kilometer-scale fluctuations at tidal frequencies in grounding-line position along the main trunk of Thwaites Glacier^23^. These observations (Fig. 1b) indicate that seawater penetrates several kilometers beneath grounded ice, episodically lifting it during tidal cycles to form a dynamic “grounding zone" characterized by active flushing of pressurized, seawater of mCDW origin. Past modeling studies suggested that kilometer-scale seawater intrusions at the grounding zone could substantially enhance ice mass loss^24–28^. Yet, many ice-sheet models treat the grounding line as a discrete boundary between grounded and floating ice and do not represent dynamic, actively-melting grounding zones. In addition, commonly used ocean-induced melt parameterizations are based on simplified plume models in which the melt rates, in many implementations, decrease toward zero at the grounding line by construction^29^. In contrast, recent satellite observations^3,30^ and high-resolution ocean modeling^31,32^ indicate that ocean-driven melt is not only substantial in close proximity and within the grounding zone, it may even be a dominant factor in glacier dynamics^27^.

Here, we quantify the contribution of melt induced by seawater intrusions beneath grounded ice to the historical retreat of Thwaites Glacier from 1996 to 2023 using the Ice-sheet and Sea-level System Model (ISSM)^33^. We implement actively-melting grounding zones in ISSM (Fig. 1c), building on past numerical frameworks^25,28^. Glacier sensitivity over the historical period is evaluated across a range of grounding-zone lengths and submarine melt rates using a Bayesian calibration approach (Methods). To evaluate the model, we compare the results with three key independent observational records: (i) surface velocity^34^, (ii) ice thickness^35^, and (iii) position of the grounding line^36^. Together, these independent constraints provide a stringent test of the model fidelity, enabling a characterization of historical changes in the glacier state. Following model calibration on the hindcast ensemble, we extend the simulations to 2100 to estimate the contribution of Thwaites Glacier to global sea-level rise under a controlled, constant climate forcing baseline (i.e., a lower-bound scenario) that isolates the role of grounding-zone melting from future ocean-warming uncertainty.

## Results

### Reconstructing historical changes of the glacier state

The most extensive retreat of the Thwaites Glacier has occurred along its western and fastest-flowing sector, the Thwaites Main Trunk (TMT), which has migrated inland along a deep bathymetric channel at a rate of ~800 m yr^−1^, or 25 km since 1996 (Fig. 1b and Supplementary Fig. 1). Currently, the glacier remains pinned on the Mouginot Ridge, a bathymetric high of a few km in size located roughly 950 m below sea level^23^. High-resolution ICEYE SAR observations have shown  frequent, albeit temporary, ungrounding extending up to 10 km inland of Mouginot Ridge (Fig. 1b and Supplementary Fig. 1), indicating recurrent kilometer-scale seawater intrusions beneath grounded ice. In contrast, the eastern sector of the glacier, the Thwaites Eastern Ice Shelf (TEIS), has undergone a slower and more spatially heterogeneous retreat at a rate of 400–700 m yr^−1^ since 1996, corresponding to 10–20 km^3,36^.

**Fig. 2:**
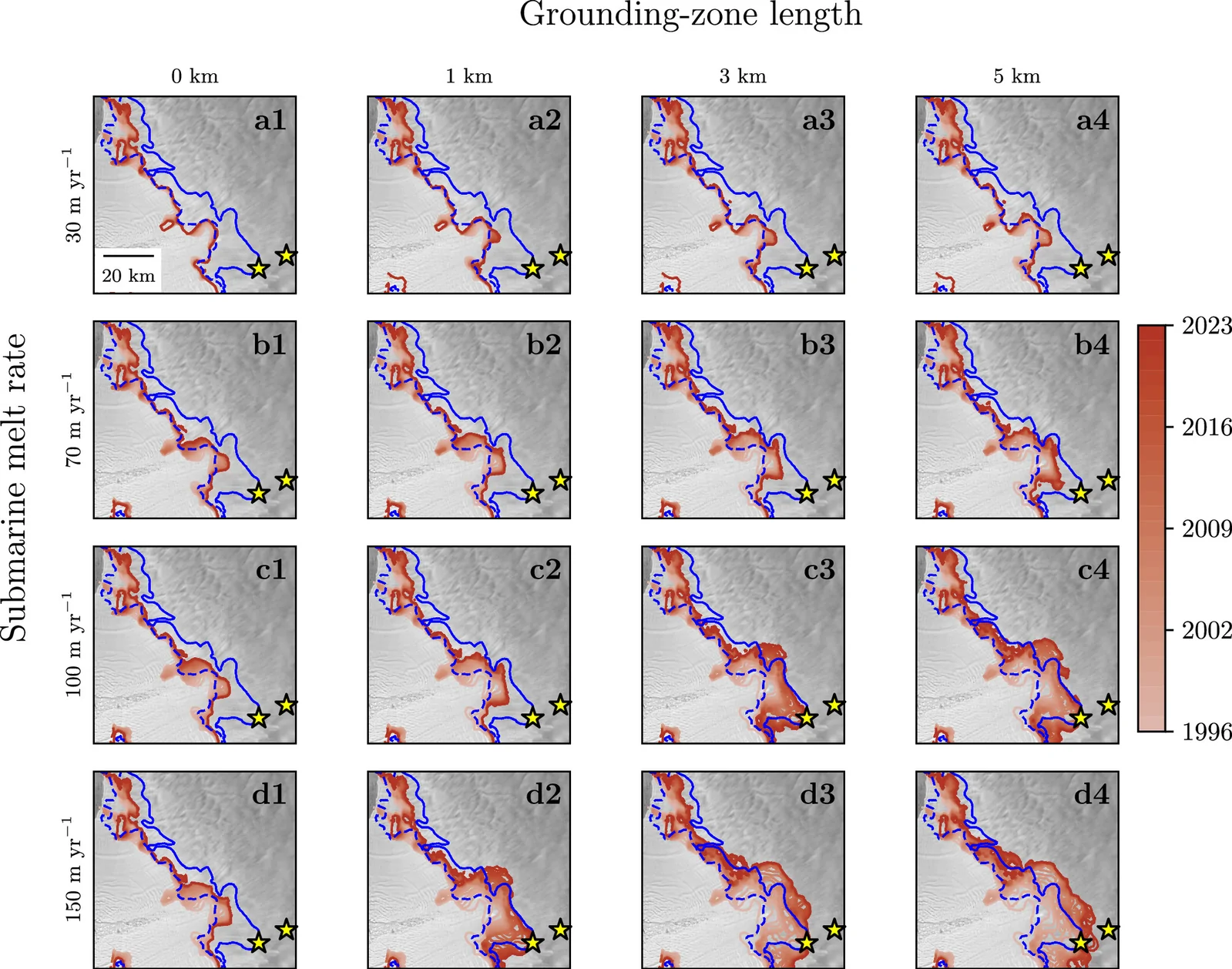
Reconstruction of Thwaites’ historical retreat. Color-coded lines are the modeled groundingline location from 1996 to 2023. The dashed and solid blue lines are observed locations in 1996 and 2023, respectively^23^. Yellow stars are the location of Mouginot Ridge and South Ridge. Background is the RADARSAT-derived SAR mosaic.

Using ISSM initialized to a state representative of the mid-1990s (Fig. 1a and Supplementary Fig. 2, 3, Methods), we simulate the evolution of the Thwaites Glacier until 2023 (Fig. 2). Ice flow is governed by a regularized Coulomb friction law, which best reproduces the acceleration of the neighboring Pine Island Glacier^13,37^. In this configuration, we explicitly represent an actively-melting grounding zone (Fig. 1C, Methods) that allows ocean-driven basal melt to occur inland of the flotation line calculated by the model, in agreement with satellite observations^23,31,38^, although we do not account for the associated reduction in basal friction due to enhanced lubrication by seawater intrusion and/or subglacial discharge. Model results indicate that the rate of grounding-line retreat is strongly controlled by both the basal melt rate beneath floating ice and the length of seawater intrusions into the grounding zone (Fig. 2), in agreement with previous studies^10,24,25,28^. Overall, higher melt rates and intrusions with length scales comparable to the ridge size lead to greater retreat by extending grounding-zone melting across a larger portion of the stabilizing ridge.

**Fig. 3:**
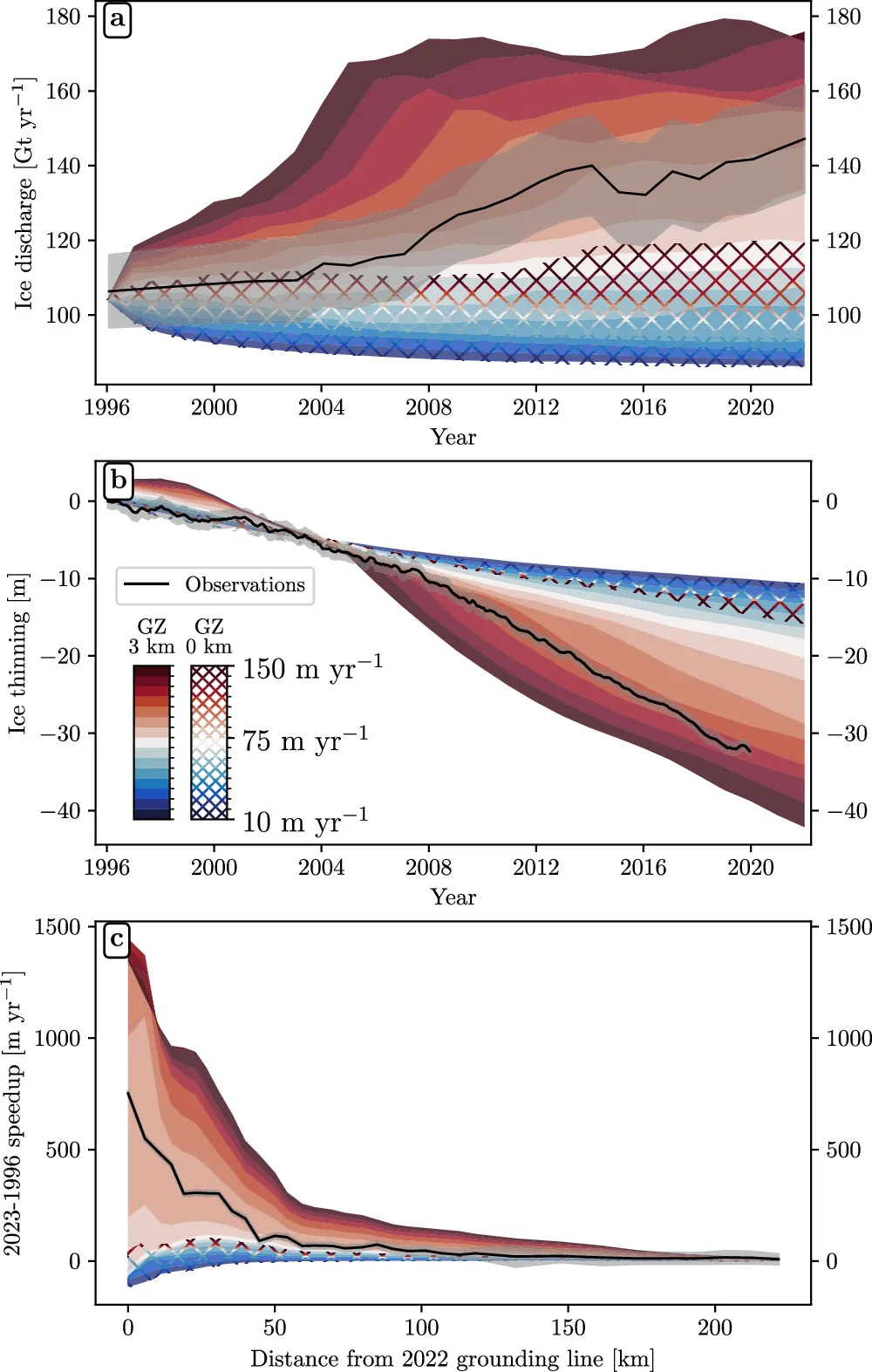
Reconstruction of past ice discharge, glacier thinning and ice-flow speed-up. Solid colored areas are model results with a melt rate range between 10 and 150 m yr^−1^ (color-coded) and a grounding-zone length of 3 km (solid area) and no grounding zone (hatched area). **a** Ice discharge at the grounding line. **b** Glacier thinning calculated as the average over grounded ice in the model domain. **c** Glacier speed-up along the fastest moving sector of Thwaites (i.e., along the dashed black flowline in Fig. 1a). Black lines with gray shades are observed time series and associated error bar from^1^ in **a,**^35^ in **b**, and^34^ in (**c**).

However, the model systematically overestimates the retreat of TEIS by 15 km, a bias also reported in previous modeling studies^8,10,11^, if using the state-of-the-art bed topography^2^ (Supplementary Fig. 4). When initializing the model to reconstruct the glacier state in 1996, we find that seafloor elevations under floating ice are too deep to be consistent with the grounding-line location calculated via flotation. Within the uncertainty bounds of bed topography^2^, we apply a localized 100 m lift of the seafloor in regions constrained by gravity inversion to satisfy the flotation consistency with the observed 1996 grounding line (Supplementary Fig. 4). With this correction in place, the model simulates grounding-line retreat, glacier thinning, and ice-flow acceleration even without the grounding-zone module, similarly to traditional ice-sheet models (Fig. 2). In this configuration, however, the model requires relatively high submarine melt rates^30,31^ of 150 m yr^−1^ in order to align with observations acquired between 1996 and early 2000s (Fig. 3a). Moreover, these simulations still underestimate discharge and thinning observed between 2004 and 2022 (Fig. 3a, b), the glacier acceleration (Fig. 3c) as well as the grounding-line retreat (Fig. 2).

On the other hand, explicitly resolving kilometer-scale grounding zones enables agreement with all observational constraints over the 25-year record. Using a grounding zone of 1 km in length, the model captures the concurrent retreat of both TEIS and TMT over 25 years, but with a relatively high submarine melt rate of 150 m yr^−1^ (Fig. 2d2). For a 3 km grounding zone, the ensemble simulations fully reproduce the concurrent retreat of TEIS and TMT at a submarine melt rate of 120 m yr^−1^ (Fig. 2c3), and capture the associated ice discharge (Fig. 3a). Furthermore, a melt rate of 100-120 m yr^−1^, which is consistent with satellite observations (Supplementary Fig. 5) and ocean modeling^31^, yields modeled thinning that closely matches the 32 m of grounded-ice thinning observed over the simulated 25 years and calculated as a spatial average over the model domain (Fig. 3b).

**Fig. 4:**
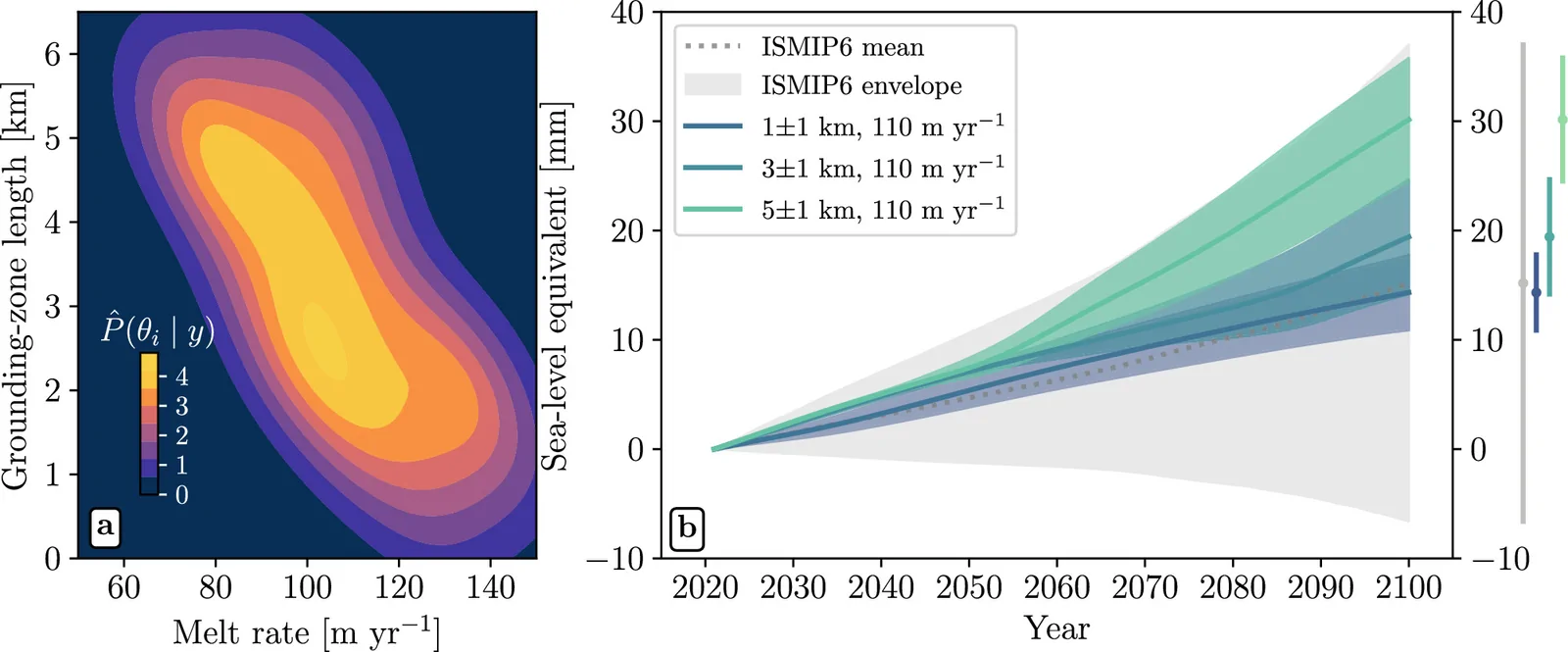
Bayesian posterior distribution and sea-level projections. **a** Normalized posterior distribution in a Bayesian framework, where $\hat{P}({\theta }_{i}| y)$ is the probability distribution of the parameter ***θ***_*i*_ (melt rate and grounding-zone length) conditioned on the observed historical trend *y*. **b** Ensemble of sea-level equivalent from model simulations and the ISMIP6 envelope under constant forcing scenario^41^. Colored lines are simulations for different grounding-zone length and melt rate of 110 m yr^−1^.

Overall, a Bayesian analysis (Methods) reveals a well-constrained region of joint parameter space for grounding-zone length and submarine melt rate that successfully and simultaneously reproduces the observed grounding-line retreat, ice thinning, and glacier acceleration (Fig. 4a, Supplementary Fig. 6 and Supplementary Tables 1–4). The joint posterior distribution exhibits a tilted structure, indicating a compensatory relationship between the two parameters, as previously noted^28^. The posterior distribution identifies parameter combinations that minimize misfit with historical satellite trends, with central values of grounding-zone length between 2 and 5 km and melt rate between 90 and 120 m yr^−1^ (Fig. 4a and Supplementary Fig. 6). This structure reflects parameter trade-offs rather than a single optimal solution, underscoring the importance of jointly calibrating multiple variables to reduce parameter uncertainty^18^. Notably, simulations without a grounding zone are four times less likely to reproduce the historical trend than simulations with a grounding zone of a few kilometers (Fig. 4a).

### Simulating the future of Thwaites Glacier

**Fig. 5:**
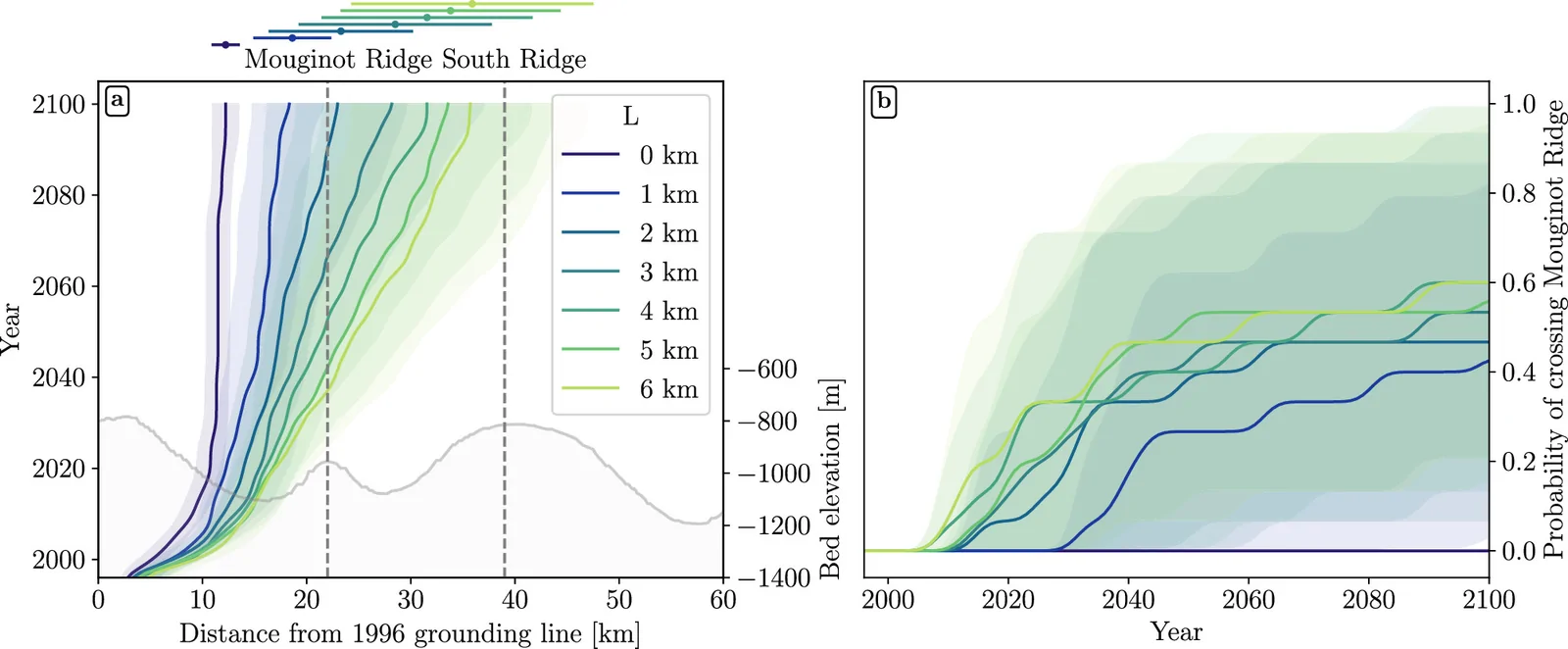
Future retreat beyond Mouginot Ridge. **a** Modeled grounding-line position relative to the observed 1996 location, calculated as the average along the fastest moving sector of Thwaites (i.e., along the dashed black flowline in Fig. 1a). Each line (shaded area) represents the average (standard deviation) along all melt-rate simulations and grouped by individual grounding-zone lengths. **b** Cumulative probability of grounding-line retreat beyond Mouginot Ridge for different grounding-zone length, before 2100.

Under a lower-bound forcing scenario derived from climate conditions averaged during the historical period 1996–2023^39^, we extend the simulations to the end of the century using the same physical configuration that successfully reproduces the observational record. We find that the retreat of the TMT is only temporarily stabilized by Mouginot Ridge (Fig. 5a and Supplementary Fig. 7). Once unpinned, the glacier continuously retreats across predominantly retrograde seafloor toward the next major topographic high, South Ridge (Fig. 1b). Mouginot Ridge therefore exerts a stabilizing influence on the present-day position of Thwaites Glacier, with the likelihood of detachment being controlled by the length of the grounding zone (Fig. 5a, b). Simulations that omit a grounding zone or employ a narrow 1 km length, regardless of the imposed melt rate, do not retreat beyond Mouginot Ridge by 2100 (Fig. 5a and Supplementary Fig. 7), consistent with their fourfold lower likelihood of reproducing the historical retreat (Fig. 4a).

In contrast, simulations with a 2–3 km grounding zone predict a ~45% probability of unpinning from the Mouginot Ridge by the end of the century, with simulations employing 2 km to retreat beyond the ridge on average in 2088 and in 2065 for a 3 km length (Fig. 5). On the other hand, simulations consistent with recent (2020s) satellite-inferred grounding-zone extent (4–6 km) show a higher probability ~60% of crossing the ridge by 2100. In particular, at 5–6 km, unpinning occurs as early as 2038, allowing the grounding line to reach South Ridge well before 2100. Across our model ensemble, ridge crossing is primarily controlled by intrusion lengths that are comparable to the characteristic ridge scale.

### Sea-level projections

All simulations produce sustained sea-level contributions throughout the century (Fig. 4b and Supplementary Table 5), without indication of slowdown or stabilization feedback, in agreement with previous studies^7,8^. Notably, the envelope of our projections lies in the upper half of the uncertainty range of the Ice Sheet Model Intercomparison Project for CMIP6 (ISMIP6) under the same forcing scenario^40^. The ISMIP6 projections exhibit a large uncertainty^41^, with a mean of 15.2 ± 21.8 mm of sea-level equivalent, and some simulations even project a late-century slowdown in ice discharge. Although the large spread in the ISMIP6 envelope partly reflects the use of a multi-model ensemble^40^ rather than a single modeling framework, our results further demonstrate that the inclusion of key physical processes^14^, together with calibration against historical observations, provides a reliable pathway for narrowing uncertainty in ensemble projections^9,19,22^.

In these projections, we assume that there is no interannual variability in the thermal forcing of the ocean by the end of the century. Oceanographic observations collected in the Amundsen Sea Embayment since the 1990s indicate that water temperatures at 700 m depth have remained stable in the range of 1.0 to 1.2 ^∘^C^5,42,43^, while the temperature of CDW at the edge of the continental shelf is not likely to exceed 1.5 ^∘^C^42^. As a sensitivity test, future warming could increase ocean temperatures by 0.5 to 0.7 ^∘^C compared to the conditions simulated by our model, which, assuming a linear relationship between thermal forcing and melt rates^44^, would increase basal melting by ~10 m yr^−1^. We calculate that such an increase in melt would raise projected ice loss by ~15% (Supplementary Fig. 8a, b). We therefore conclude that our projections are less sensitive to moderate changes in thermal forcing than to grounding-zone extent, with each 1 km increase in grounding-zone length producing an average 22% change in projected sea-level equivalent (Supplementary Fig. 8c, d). Notably, simply introducing a grounding zone leads to a sea-level rise increase by 51% with respect to simulations with traditional grounding-line formulations (Supplementary Fig. 8d). Lastly, this analysis does not account for potential future shoaling of the thermocline^45^, which would likely increase melt rates by exposing a larger fraction of the ice shelf base to relatively warm mCDW.

The timing of unpinning from Mouginot Ridge modulates the contribution of Thwaites Glacier to sea level^46^ (Fig. 4,5). Adopting a melt rate of 110 m yr^−1^ - which (i) minimizes misfit with observations (Fig. 4a), (ii) is consistent with satellite-based estimates (Supplementary Fig. 5), and (iii) aligns with ocean numerical models^31 ^- we project that simulations omitting a grounding zone, yields a sea-level contribution of 8.3 ± 3.0 mm, which is lower than the ISMIP6 mean ensemble (Supplementary Table 5, Figure 4b) and inconsistent with the calibrated hindcast constraints. On the other hand, prescribing a narrow, 1 ± 1 km grounding zone brings the sea-level equivalent of Thwaites to 14.3 ± 3.1 mm, which is comparable to the ISMIP6 mean. Increasing the length of the grounding-zone to 3 ± 1 km, i.e., the maximum of the Bayesian posterior distribution (Fig. 4a), we estimate a lower-bound sea-level contribution resulting in a contribution of 19.4 ± 5.2 mm by 2100, i.e., an increase of 130% relative to the mean of the ISMIP6 ensemble. A grounding-zone length of 5 ± 1 km, consistent with recent high-resolution observations^23^, with frequent documented intrusions that extend even beyond Mouginot Ridge (Fig. 1b), produces projections of 30.1 ± 5.7 mm by 2100, which is twice the mean of the ISMIP6 ensemble under a constant climate forcing scenario.

## Discussion

We present a calibrated model that reproduces independently acquired historical trends, including velocity and thickness change as well as grounding-line migration, establishing a strong benchmark for projection skill. This performance improvement arises from explicitly representing actively-melting grounding zones, rather than treating the grounding line as a discrete grounded-floating boundary. This framework also highlights sensitivity to bathymetric uncertainties that hinder accurate reconstruction of historical grounding-line positions. The results underscore that, within the observationally supported range of prescribed melt rates considered here, grounding-zone extent exerts a stronger control on retreat and unpinning than moderate variations in thermal forcing, when the latter is represented by a simple depth-dependent melt parameterization. Oceanic and geometric feedback associated with the evolving sub-shelf cavity are not included in this analysis and may further modulate melt rates during retreat^47,48^.

A remaining limitation of our simulations is the assumption of a fixed grounding-zone length, whereas the extent of the grounding zone likely varies with local bed and surface slopes, height above flotation, tidal amplitude and subglacial water pressure^23,26,38,49^. In our framework, the grounding-zone length should be interpreted as an effective, time-averaged intrusion scale that reproduces decadal evolution. Satellite observations indicate that kilometer-scale seawater intrusions have persisted over multiple years and expanded gradually over decadal timescales^36^, supporting the use of a time-averaged grounding-zone length as a first-order representation of contemporary conditions. Consistent with this interpretation, our Bayesian calibration constrains an effective grounding-zone scale that integrates these processes over the historical period. Developing a prognostic parameterization that links grounding-zone extent to evolving geometry and subglacial hydrology remains an important direction for future modeling work, but our results suggest that the mean kilometer-scale extent, rather than short-term variability, exerts the dominant control on multi-decadal retreat and ridge unpinning.

Our results are subject to limitations arising from the use of a fixed ice front and uncertainties in the initial geometry. Although calving and ice-front retreat have possibly contributed to the historical retreat of Thwaites Glacier^8,50^, our experimental design intentionally excludes these processes. By doing so, this sensitivity framework allows us to isolate the effect of active grounding-zone melting and directly assess its contribution to glacier evolution. Furthermore, our treatment of submarine melting relies on a simplified parameterization, rather than resolving ocean circulation and heat transport within evolving ice-shelf cavities. Although this approach does not capture the full complexity of the time-variable dynamics of the ocean, the melt rates adopted here are consistent with satellite-derived estimates^30^ and high-resolution ocean modeling studies^31,51^. By calibrating melt rates against observed changes in glacier state, we constrain the integrated impact of ocean forcing over multi-decadal timescales. Fully coupled ice-ocean simulations would allow a detailed representation of cavity circulation and the glacier response^10,20,47,48^, but such models remain limited by sparse observational constraints within the Thwaites Ice Shelf and are computationally expensive^32^. Comprehensive in-situ measurements of temperature, salinity and circulation beneath the ice shelf^52^ remain therefore important to reduce structural uncertainty and develop more physically complete melt parameterizations.

In conclusion, our results show that kilometer-scale, actively-melting grounding zones fundamentally alter both hindcast and forecast modeling skills for Thwaites Glacier. By explicitly resolving grounding zones and calibrating the model against multiple independent observational constraints, we reproduce the modern observational record and obtain sea-level contributions that lie toward the upper end of previously reported uncertainty ranges. Therefore, actively-melting grounding zones should be systematically incorporated in regional and continental-scale ice-sheet models, including within international intercomparison frameworks. At the same time, expanded in-situ observations near grounding zones are urgently needed^14,27^ to better constrain heat delivery to sub-ice-shelf cavities and melt dynamics. Ultimately, improving the representation of these processes is critical to narrowing the uncertainty of Antarctica’s contribution to future sea-level rise.

## Methods

### Ice-sheet model configuration

We simulate the past, present, and future dynamics and thermodynamics of Thwaites Glacier, in the Amundsen Sea Embayment sector of West Antarctica, using the Ice-sheet and Sea-level System Model (ISSM)^33^. The ice sheet model domain covers the grounded and floating portions of Thwaites and Haynes Glaciers, which are discretized using an unstructured triangular mesh (Supplementary Fig. 2a). We include Haynes Glacier within the model domain because it is dynamically connected to Thwaites Glacier along the western boundary, and the two share a continuous ice trunk. This inclusion is necessary to accurately simulate the evolution of the two glaciers. However, our analysis focuses exclusively on Thwaites, as it dominates the observed ice-loss signal^1^. The two-dimensional mesh contains 89,824 elements generated with a Bidimensional Anisotropic Mesh Generator. The element size varies from 500 m near the grounding zone to capture small-scale spatial features on fast-flowing ice to 20 km on slow-moving regions. The mesh remains fixed throughout the transient simulations. The inland extent of the 500 m resolution (Supplementary Fig. 2a) is selected to capture the full range of grounding-zone migration over the simulation period, ensuring that the grounding zone is always modeled at 500 m resolution.

We reconstruct and initialize the state of the glacier system by configuring ISSM with conditions representative of the 1990s. We assimilate observational data from that period, where and when available. The model domain is based on a 1996–2001 velocity map derived from ERS-1/2 and RADARSAT-1 SAR data^53^, using a fixed ice front. The surface elevation is from the Antarctic Digital Elevation Model (DEM) produced from ERS-1 radar altimetry during the 1994–1995 geodetic phase^54^. The thickness of the ice and the transition from floating to grounded ice are derived from the historical DEM assuming hydrostatic equilibrium. The seafloor elevation is from Bedmachine v3.7^2^, which uses reconstructions based on circumpolar gravity anomalies under floating ice^55^ and mass conservation on grounded ice. To reproduce the position of the grounding line observed with double-difference interferometric SAR in 1996, the elevation of the seafloor is increased by up to 100 m where Bedmachine v3.7 uses gravity inversion, that is under floating ice, with seafloor depth of ~1000 m. The correction of 100 m is within the errors of gravity inversion techniques^55^. We define an average “observed" grounding-line location for 1996 from a set of 10 grounding lines retrieved from ERS-1 and ERS-2 between 1992 and 1996. For the observed position in 2021–2023, we use a long time series of short-time ICEYE repeat pass data.

We derive ice hardness for floating ice and basal friction coefficient for grounded ice (Supplementary Fig. 2c) using inversion methods that minimize the discrepancy between the modeled and observed surface velocity (Supplementary Fig. 2d), here the SAR-derived 1996–2001 glacier velocity map^53^. To simulate glacier flow, we use the 2-D Shelfy-Stream Approximation^33^ (SSA). To model sliding at the glacier base, we select a regularized Coulomb friction law^56^, which represents the relationship between the basal shear stress (*τ*_*b*_) and the basal ice velocity (*u*_*b*_) as:

1 $${\tau }_{b}=\frac{{C}_{s}| {u}_{b}{| }^{\frac{1}{m}-1}{u}_{b}}{{(1+\frac{{C}_{s}}{{C}_{max}N}| {u}_{b}| )}^{\frac{1}{m}}}$$

where *C*_*s*_ is the friction coefficient, *N* is the effective pressure, computed as the weight of the ice column, and *C*_*max*_ is the upper limit of the Iken’s bound (*τ*_*b*_/*N*)^57^. Maximum misfits of ~800 m yr^−1^ occur over the floating tongue, primarily within the flexure zone, where the model assumes hydrostatic flotation despite the ice not being fully afloat. The use of a regularized Coulomb friction law (with *m* = 3) over a Budd- or Weertman-style friction law was shown to be better able to reproduce the historical speed-up of the Pine Island Glacier^13^, located 250 km north of Thwaites Glacier. Note that the friction coefficient is kept constant over the simulated time, and we do not include the impact of subglacial hydrology networks. The effective pressure *N* is indeed calculated as the ice overburden pressure, effectively representing either a frozen bed or a highly efficient drainage network into underlying aquifers. Importantly, however, Coulomb-limited sliding is activated primarily in the fastest-flowing regions near the grounding zone, leaving areas where water pressure approaches the overburden pressure (i.e., low *N*) governed by the *m* = 3 power-law sliding relationship. Consequently, the prescribed overburden effective pressure has a limited influence on the simulated sliding over the grounded domain. Nevertheless, these remain limitations of the model, as the use of a time-invariant basal friction coefficient that does not directly depend on basal lubrication may lead to an underestimation of ice thinning and speed-up over the course of the transient simulation by construction^21^.

To retrieve the hardness of grounded ice, we extrude the domain into a three-dimensional model and use the Blatter-Pattyn Higher-Order (HO) stress balance approximation^33^ with 10 vertical terrain-following levels to initialize the ice temperature based on an enthalpy thermal model^58^. To initialize the thermal model, we solve the higher-order stress balance to obtain three-dimensional velocity and pressure fields, then run the thermal model to steady state forced by the geothermal heat flux^59^. The resulting temperature field is depth-averaged to inform the ice hardness on grounded ice.

### Seawater intrusions at the grounding zone

We introduce the concept of seawater intrusions under grounded ice in ISSM (Fig. 1c), building on^24,25,28^. Basal melt in the km-wide grounding zone (${\dot{m}}_{x}$) decreases linearly from a maximum value at the grounding line ${\dot{m}}_{{{{\rm{GL}}}}}$, calculated by hydrostatic equilibrium^60^, to zero at a specified intrusion distance L:

2 $${\dot{m}}_{x}={\dot{m}}_{{{{\rm{GL}}}}}\left(1-\frac{x}{{{{\rm{L}}}}}\right)$$

Basal melt, governed by Eq. (2), is applied to each element upstream of the flotation line (Fig. 1c), while the linear decrease in melt with distance is consistent with high-resolution ocean modeling^31^.

In simulations without a grounding zone, the location of the grounding line within individual elements is tracked using the migration parameterization of the SEM2 sub-element grounding line, where the basal friction is integrated over the grounded portion of an element^61^. We extend the parameterization of SEM2 at the end of the grounding zone, i.e., at the intrusion distance L, effectively simulating the end of the grounding zone as an element with partial melting. This implementation is freely available in ISSM v2026.1^33^.

Note that the basal friction coefficient is not modified within the grounding zone, despite the expectation that seawater intrusion reduces basal resistance through enhanced lubrication. In standard inversions, the region immediately inland of the grounding line is often inferred to have high basal friction, effectively pinning the glacier to the seafloor. Our approach instead generates enhanced melt in this region, weakening this pinning effect by ungrounding the glacier from stabilizing bedrock highs through construction. Future developments of this module should incorporate a parameterization that decreases the friction coefficient in regions affected by seawater intrusion and/or subglacial discharge to better represent basal lubrication.

### Hindcast ensemble

After configuring the model, we perform a 6-year ice sheet spin-up (Supplementary Fig. 3) using a constant surface mass balance prescribed by the regional atmospheric model Glacier Energy and Mass Balance (GEMB)^39^, based on average climate conditions for the period 1996–2023, and a fixed basal melt rate of 10 m yr^−1^. The forcing of climate at the ice surface and the relatively low basal melt rate under the ice shelf allow the dynamic model to relax to the imposed initial conditions. The short spin-up is necessary to ensure that i) the initial modeled grounding line matches its observed location in 1996 (Supplementary Fig. 3a) and ii) the modeled loss at the grounding line agrees with the observed mass loss in the mid 1990s (Supplementary Fig. 3b). The spun-up model reproduces the observed initial ice volume to within 1.4% from the observed state, providing confidence that the initial state is still representative of the historical glacier geometry. We acknowledge that short spin-up is a modeling choice, and extending it forward would continue to modify the initial conditions through model drift and therefore lead to a different transient evolution. To this end, the bathymetric correction of 100 m under floating ice is also necessary for the model to adjust to the 1996 conditions (Supplementary Fig. 4 and discussion in the manuscript), without impacting the melt parameterization, as seafloor under floating ice is below the parameterization cap of 900 m^55^. Following the short spin-up, we run a suite of numerical simulations under constant-forcing baseline with an average surface mass balance for 1996–2023^39^. Finally, we use a fixed time-step of two weeks to ensure numerical convergence.

The goal of the hindcast exercise is to reproduce three independently-obtained satellite trends: surface velocity^34^, thickness change^35^, and grounding-line migration^36^. It is important to note that the last readily-observable metric to assess the full state of the glacier is the location of the ice front, as calving could lead to buttress removal and glacier acceleration. This aspect, however, is not investigated in this study as the ice front is fixed throughout the simulation. Similarly, we do not investigate the impact of ungrounding of pinning points near the ice front of TEIS and TMT, which could alter the buttressing force of the ice shelf^50^. In the hindcast simulations, we focus and isolate the sensitivity of the historical changes to the following parameters ${{{{\boldsymbol{\theta }}}}}_{i}=({\dot{m}}_{{{{\rm{GL}}}}},\,{\mbox{L}})$, where ${\dot{m}}_{{{{\rm{GL}}}}}$ is the maximum melting rate at the hydrostatically-derived grounding line^60^ and L is the length of the grounding zone, following the numerical framework of^28^. We perform a total of 105 simulations combining ${\dot{m}}_{{{{\rm{GL}}}}}$ and L.

### Experiment set-up

The ocean-driven melting of floating ice is represented using a depth-dependent linear basal melt parameterization^10^, with a maximum melt rate (i.e., ${\dot{m}}_{{{{\rm{GL}}}}}$ in Eq. (2)) at and below 900 m, linearly decreasing to zero at 300 m. No basal melt is applied to floating ice shallower than 300 m. The maximum melt rate ${\dot{m}}_{{{{\rm{GL}}}}}$ at 900 m ranges from 10 m yr^−1^ to 150 m yr^−1^ in increments of 10 m yr^−1^. This parameterization reproduces the amplitude of enhanced melting at depth near the grounding line (${\dot{m}}_{{{{\rm{GL}}}}}$ in Eq. (2)) inferred from satellite observations^30^ (Supplementary Fig. 5) and numerical models^51^, although it is less sophisticated than fully coupled ocean circulation models^10,47,48^. Melt rates in the eastern sector of Thwaites Glacier (TEIS) have a maximum melt of 10 m yr^−1^, following robotic observations near the grounding zone^52^. This assumption is supported by observations showing that mCDW in this region is found at roughly 580 m depth, with in-situ data showing suppressed melting in this area^52^, also consistent with satellite data (Supplementary Fig. 5)^30^, and ocean modeling results^51^. Because the grounding-line positions from the 1990s to today lie at shallower depths in some areas, mCDW - the main origin of melting in the area - would struggle to spill above the current grounding-line location and into the glacier basin. Any such intrusion would most likely occur through the deep bathymetric channel beneath the TMT, but it would take centuries to reach the eastern sector^46^.

Regarding grounding-zone length (i.e., L in Eq. (2)), we test the sensitivity of glacier dynamics using scenarios without a grounding zone and with lengths ranging from 1 km to 6 km, varying in 1 km increments. These values are consistent with typical lengths of seawater intrusions documented with satellite observations in the Amundsen Sea Embayment^23^ and in other sectors of Antarctica^38,49^.

### Bayesian calibration and sea-level projections

To identify the combinations of melt rate and grounding-zone length that are most likely to explain historical trends, we employ a Bayesian calibration approach following the framework of^16,17^. The Bayes theorem is written as:

3 $$\hat{P}({{{{\boldsymbol{\theta }}}}}_{i}| y)=\left\Vert \frac{{{{\mathcal{L}}}}(y| {{{{\boldsymbol{\theta }}}}}_{i})\,P({{{{\boldsymbol{\theta }}}}}_{i})}{P(y)}\right\Vert$$

where $\hat{P}({{{{\boldsymbol{\theta }}}}}_{i}| y)$ is the posterior distribution, representing the probability of the parameter ***θ***_*i*_ conditioned on the observed data *y*; ${{{\mathcal{L}}}}(y| {{{{\boldsymbol{\theta }}}}}_{i})$ is the likelihood, quantifying to what extent the set of model parameters ***θ***_*i*_ reproduces the observed data *y*; *P*(***θ***_*i*_) is the prior distribution, reflecting the initial uncertainty about the parameter before observing the data. Finally, *P*(*y*) is the marginal likelihood, calculated as the sum of all tested parameter combinations and serving as a normalization constant.

We begin by generating a uniform (randomly selected) prior ensemble consisting of 10,000 samples of melt rate and grounding-zone length, which are the parameters ***θ***_*i*_. These are drawn uniformly from the hindcast sensitivity grid, with a melt rate ${\dot{m}}_{{{{\rm{GL}}}}} \sim {{{\mathcal{U}}}}(10,150)$ m yr^−1^ and a grounding-zone length $\,{\mbox{L}}\, \sim {{{\mathcal{U}}}}(0,6000)$ m. To ensure statistical robustness, the model outputs are linearly interpolated from the uniform space ***θ***_*i*_ to construct a set of surrogate models *f*, which reproduce the key observables:

4 $$f:({\dot{m}}_{{{{\rm{GL}}}}},\,{\mbox{L}})\mapsto \left[{R}_{{{{\rm{TMT}}}}},{R}_{{{{\rm{TEIS}}}}},D,T,S\right],$$

where *R* denotes the retreat of the grounding line (for TMT and TEIS), *D* denotes the ice discharge at the grounding line, *T* is the thickness change of the glacier, and *S* is the alteration in glacier speed.

For each parameter sample ${{{{\boldsymbol{\theta }}}}}_{i}=({\dot{m}}_{{{{\rm{GL}}}}},\,{\mbox{L}})$, we evaluate the modeled output (*R*, *D*, *T*, *S*) using the surrogate model *f* and compare them with observational values using a Gaussian likelihood function:

5 $${{{\mathcal{L}}}}(y| {{{{\boldsymbol{\theta }}}}}_{i})=\frac{1}{\sigma \sqrt{2\pi }}\exp \left[-\frac{1}{2}{\left(\frac{f({{{{\boldsymbol{\theta }}}}}_{i})-y}{\sigma }\right)}^{2}\right],$$

where *y* is the observed value of grounding-line retreat *R*, ice discharge *D*, thickness *T* and speed changes *S*. *σ* is the uncertainty (standard deviation) that is associated with each individual observable *y*. Assuming uniform priors, the posterior weights are proportional to the normalized likelihoods:

6 $${w}_{i}=\frac{{{{\mathcal{L}}}}({{{\boldsymbol{\theta }}}}i)}{\mathop{\sum }_{j=1}^{N}{{{\mathcal{L}}}}({{{{\boldsymbol{\theta }}}}}_{j})}.$$

We perform resampling from the prior ensemble using these weights to obtain posterior distributions $\hat{P}({{{{\boldsymbol{\theta }}}}}_{i})$, which are smoothed into a continuous curve and are used to identify the most likely combination of parameters.

For each simulation, we extend the model until the year 2100 to estimate the contribution to sea-level rise, calculated from the volume of ice lost above flotation in the form of a sea-level equivalent. Using the posterior distribution *P*(***θ***_*i*_∣*y*) obtained from the Bayesian calibration to weight the most likely simulations, we identify the high-density parameter combinations ${{{{\boldsymbol{\theta }}}}}_{i}=({\dot{m}}_{{{{\rm{GL}}}}},\,{\mbox{L}})$ that are most consistent with the data and extract the corresponding sea-level trajectories.

### Satellite observations

We use a suite of satellite-derived datasets to evaluate the likelihood of model results as a historical trend *y* in the Bayesian framework. Ice surface velocities are obtained from Synthetic Aperture Radar (SAR) interferometry^53^, providing high-resolution ice motion across grounded and floating ice as an average between 1996 and 2001 using ERS-1/2 and RADARSAT-1 SAR data, and between 2020–2022 using Sentinel1-a/b, RADARSAT-2, and Landsat-8 data. Glacier speed-up is calculated along a flowline in reference to the average velocity in 1996–2001.

The position of the grounding line between 1996 and 2023 (Supplementary Fig. 1) is retrieved with quadruple differential Intereferometric SAR (InSAR). For the 1990s, we use ERS-1 and ERS-2 tandem data. For 2023-2025, we use the ICEYE constellation^23^ on the main trunk and RADARSAT Constellation Mission (RCM) data on the eastern ice shelf. To quantify changes in ice thickness, we use incorporated altimetry data from ICESat, ICESat-2, and CryoSat^35^. The ice discharge at the grounding line is calculated using the vector velocity and ice thickness as a mass flux at the grounding line, following the approach of ref. ^1^.

## Supplementary information

Supplementary Information

Transparent Peer Review file

## Source data

Source Data

## Data Availability

Ice sheet velocity data are available at ref. ^34^, glacier elevation change is available at ref. ^35^, grounding-line locations and grounding zone extensions are available at ref. ^36^. Source data are provided with this paper.

## Code availability

The Ice-Sheet and Sea-Level System model (ISSM) is freely available on Github/Zenodo^62^, together with the grounding-zone parameterization. Code to reproduce the model simulations and model output are available on Zenodo^63^. The raw data for generating the figures are available under Source Data.
