# Supplementary material for: Sea-level contribution from Thwaites Glacier doubles in an observation-constrained ice-sheet model
Source: Nat Commun. 2026 Oct 12;17:10364. doi: 10.1038/s41467-026-77976-1 (PMC13658800; doi:10.1038/s41467-026-77976-1)
Supplement: Supplementary file 1 [file 41467_2026_77976_MOESM1_ESM.pdf]

Supplementary Information: Sea-level contribution from Thwaites Glacier doubles in an observation-constrained ice-sheet model

Mattia Poinelli,[1,2,*] Eric Rignot,[1], Hélène Seroussi[3],
Josh Cuzzone[4], Tyler Pelle[5], Eric Larour[2]

[1]Department of Earth System Science, University of California, Irvine, CA, USA
[2]Jet Propulsion Laboratory, California Institute of Technology, Pasadena, CA, USA
[3]Thayer School of Engineering, Dartmouth College, Hanover, NH, USA
[4]Joint Institute for Regional Earth System Science and Engineering, University of California, Los Angeles, CA, USA
[5]Scripps Institute of Oceanography, University of California, San Diego, CA, USA
[*]To whom correspondence should be addressed; E-mail: mattiap@ucla.edu

This PDF contains:

- Supplementary Figures 1 to 8
- Supplementary Tables 1 to 5
- References

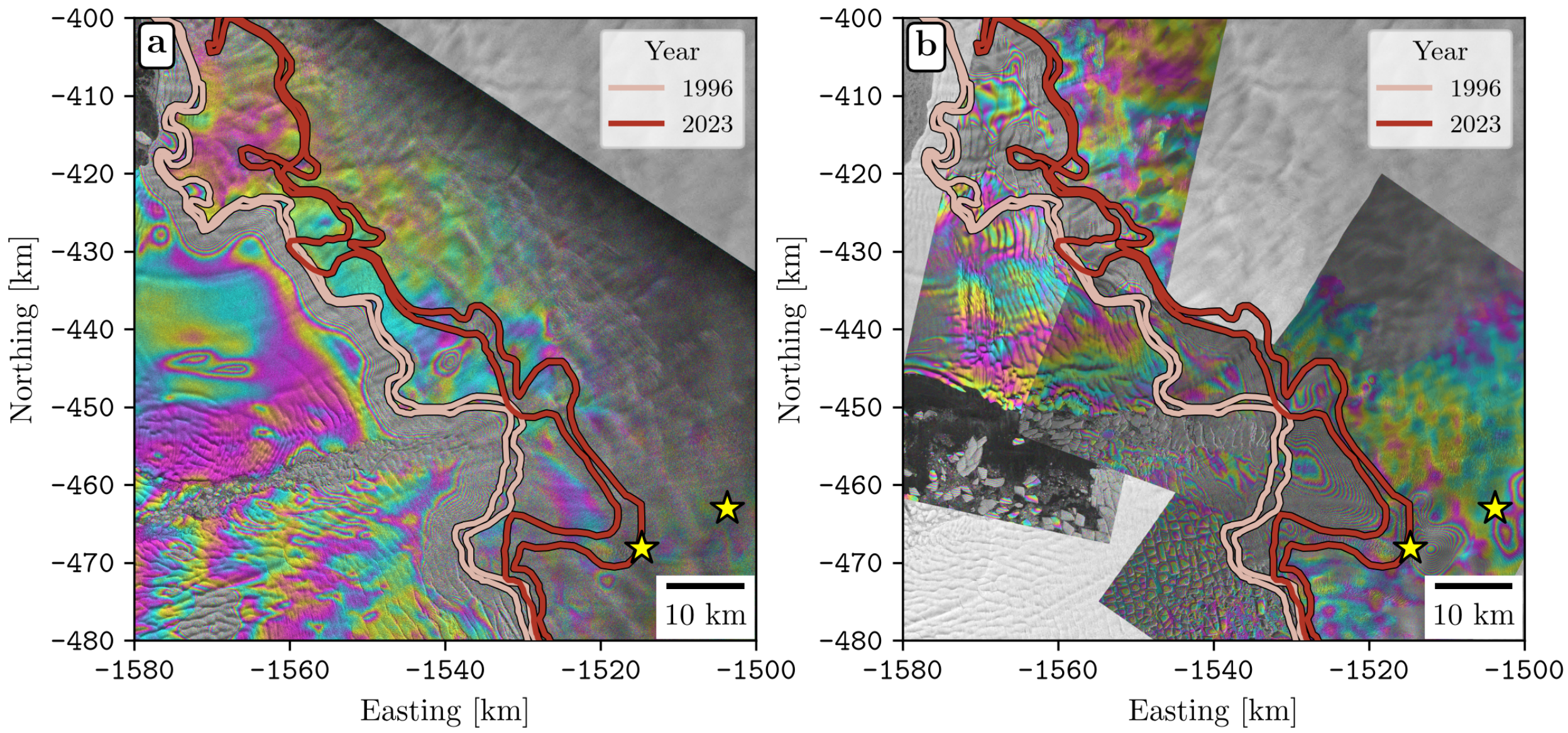

Supplementary Figure 1. Retreat of Thwaites Glacier from satellite observations. Grounding-zone extents of Thwaites Glacier inferred for A) year 1994-1996 (light red boundaries) using Earth Remote Sensing satellite 1 and 2 interferometric SAR data at the C-band frequency (5.6 cm wavelength) overlaid on a differential interferogram from 1994 combining orbits 13726, 13769, 13812, 13855, Frames 5193 5211 acquired March 01, 03, 07, and 10 1994; B) year 2023 (dark red boundaries) using RADARSAT Constellation Mission C-band SAR data and ICEYE US X-band (3.1 cm wavelength) SAR data acquired over a period of several months overlaid on a composite set of differential interferograms from ICEYE (May 12, 13, 14, 2023) on the Thwaites Main Trunk and RCM (April 19, 23 and 27, 2023) on the Thwaites Eastern Ice Shelf. Copyright ESA, CSA, and ICEYE US.

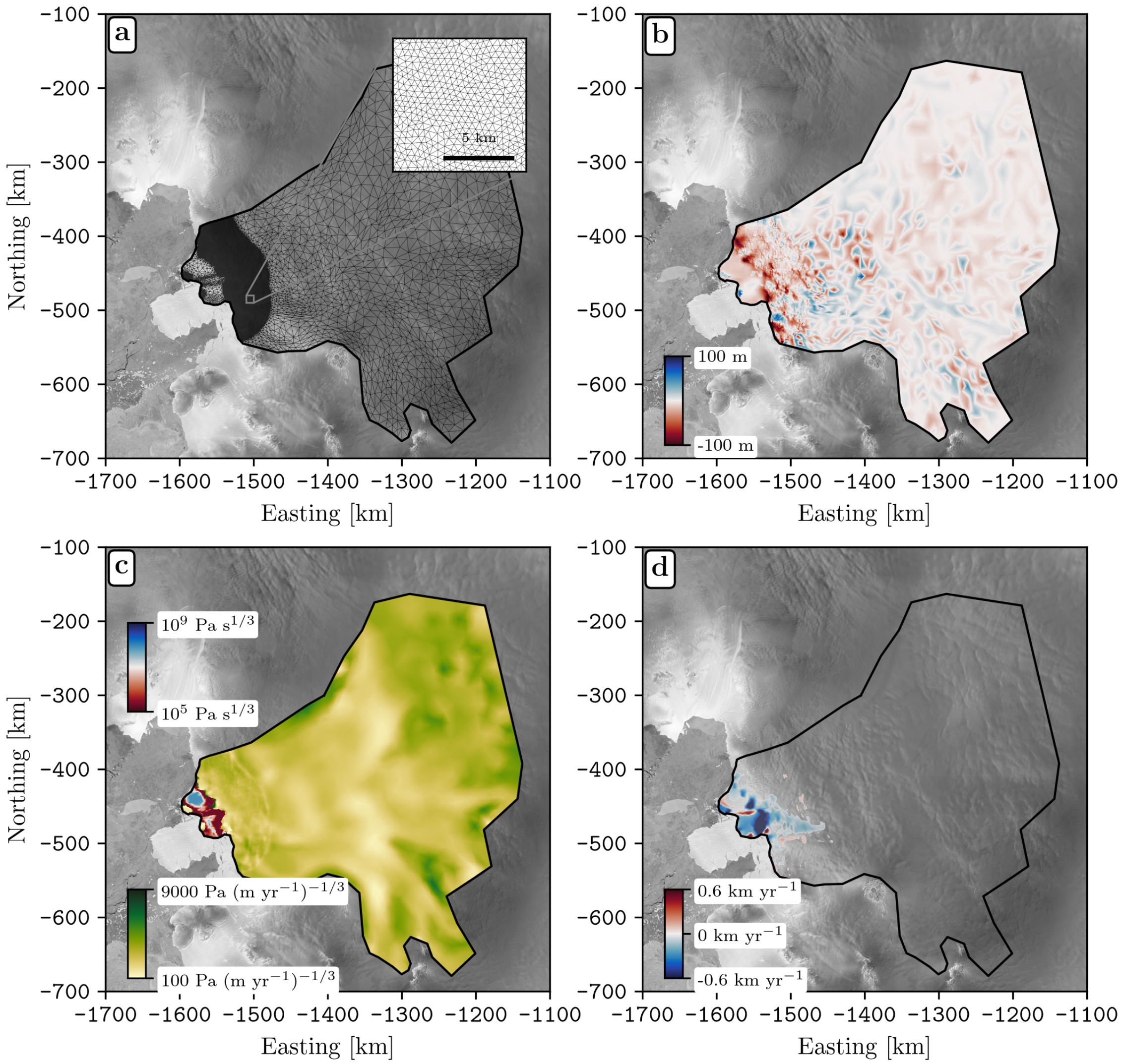

Supplementary Figure 2. Model configuration and inversion. A) Finite element mesh plotted on the model domain, with a zoom-in on areas around the grounding zone where mesh size is fixed at 500 m. (B) Difference between ice sheet thickness at the beginning of the spin-up and the initial geometry from (*1*). (C) Results of the inversion: ice hardness B on floating ice and friction coefficient C_s on grounded ice. (D) Model-data misfit in terms of ice speed after the inversions for friction and ice rheology. Transparent data is misfit below 30 m yr^{-1}. Black line is the model domain. Background is the RADARSAT-1 mosaic of Antarctica.

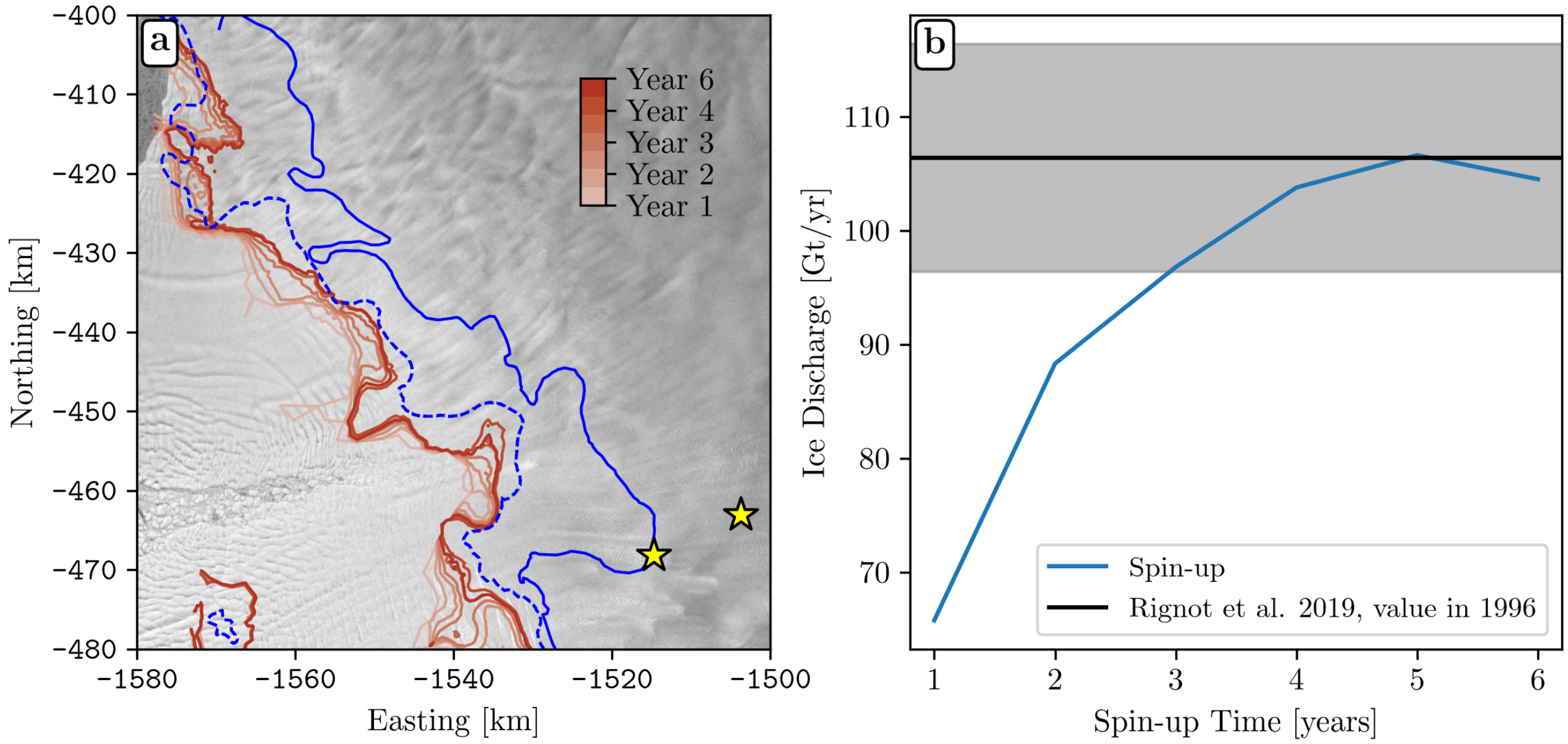

Supplementary Figure 3. Model spin-up. A) Modeled grounding line retreat during the spin-up phase (color-coded). Dashed blue line is the grounding line location in 1996 (*2*), solid blue line is the grounding line location in 2023 (*3*). Yellow stars are the location of Mouginot Ridge and South Ridge. Background is the RADARSAT-1 mosaic of Antarctica. (B) Ice discharge during the spin-up phase. Black line is observed ice discharge in 1996 and shaded area is the standard deviation (*4*).

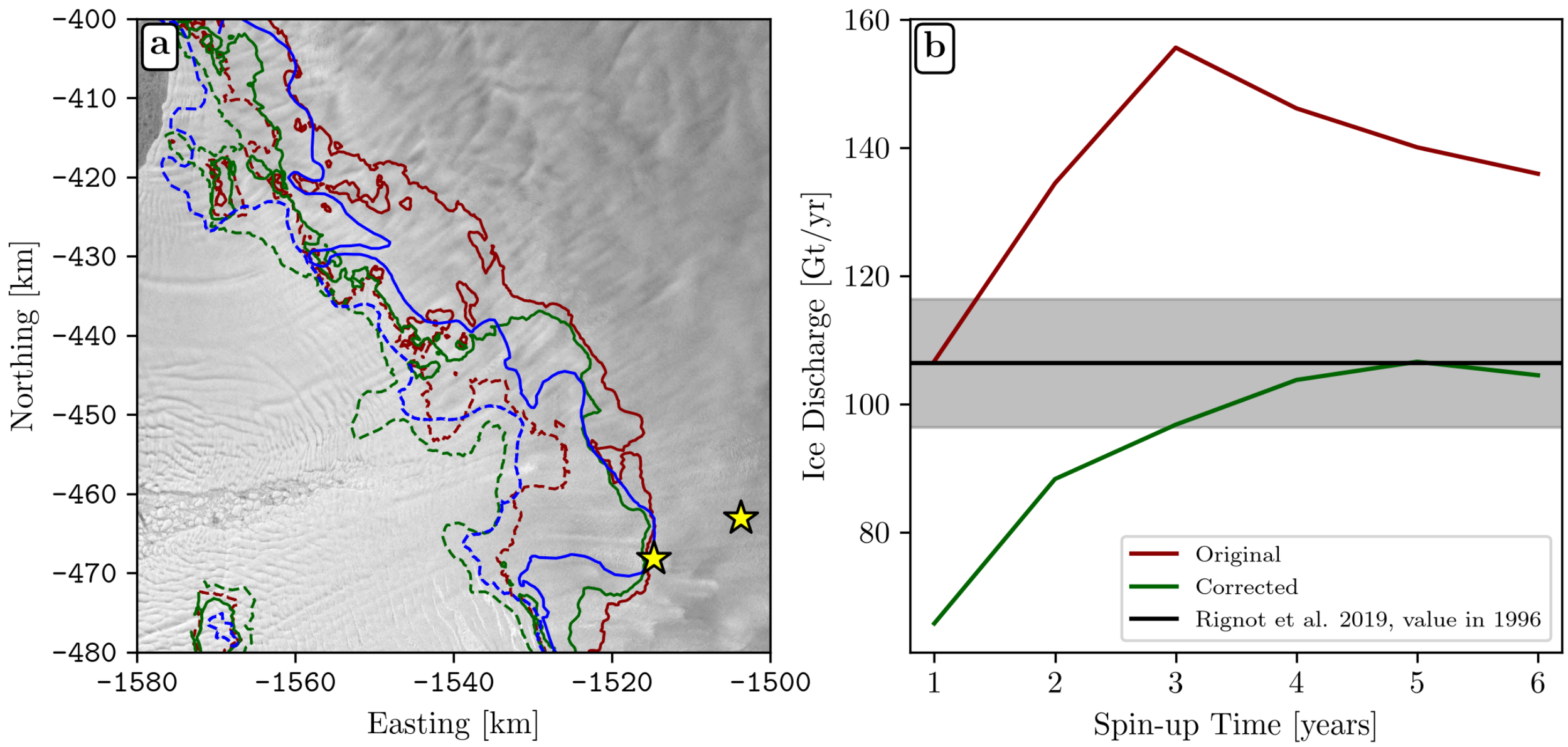

Supplementary Figure 4: Implications of the 100 m seafloor correction. **a** Modeled grounding line location for maximum a-posteriori simulations (melt rate of 100 m yr^{-1} and grounding zone of 3 km) in 2023 for model run with original bathymetry (*5*) (solid red line), and with 100 m lifting under floating ice (solid green line). Dashed green and red lines are the grounding line location at the beginning of the hindcast exercise, representative of 1996. Dashed blue line is the observed grounding line location in 1996 (*6*), solid blue line is the observed grounding line location in 2023 (*3*). Yellow stars are the location of Mouginot Ridge and South Ridge. Background is the RADARSAT-1 mosaic of Antarctica. **b** Ice discharge for simulation with original bed (red line) and with 100 m lifting under floating ice (green line). Black line is observed ice discharge in 1996 and shaded area is the standard deviation (*4*).

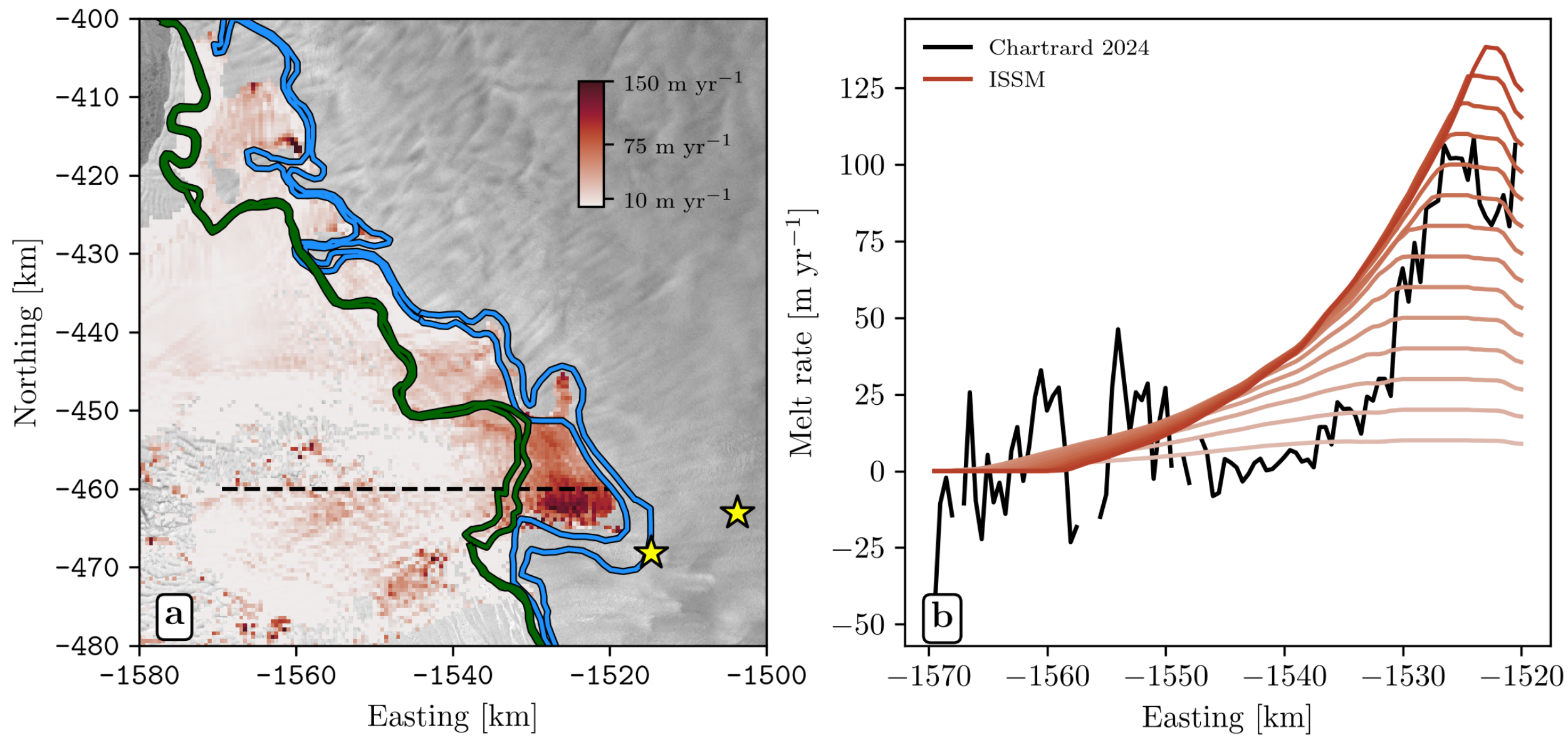

Supplementary Figure 5: Model-data comparison. **a** Basal melt rate as average between 2010-2021 from satellite observations (*7*). Dark green line is the extent of the grounding zone in 1996 on Thwaites Main Trunk and Eastern Ice Shelf from satellite observations (*6*). Blue line is the extent of the grounding zone in 2023 (*3*). **b** Observed melt rate (*7*) (black line) and the prescribed depth-dependent melt-rate parameterizations (red lines), with different shades indicating different maximum prescribed melt rates, along the dashed transect shown in panel **a**.

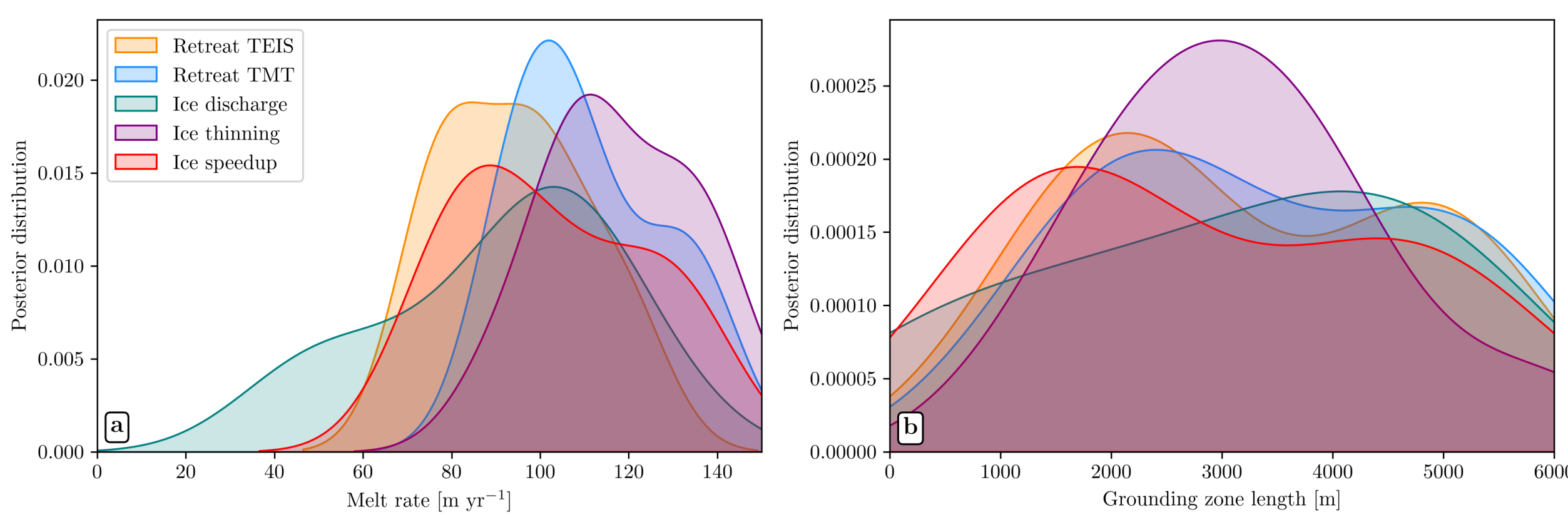

Supplementary Figure 6: Bayesian calibration. Posterior density function $\hat{P}(\boldsymbol{\theta}_i \mid y)$, representing the probability of simulations employing the parameter θ_i to reproduce the observed data y. The discrete distribution is smoothed in a continuous curve. In **a**, θ_i is melt rate; in **b**, θ_i is grounding-zone length.

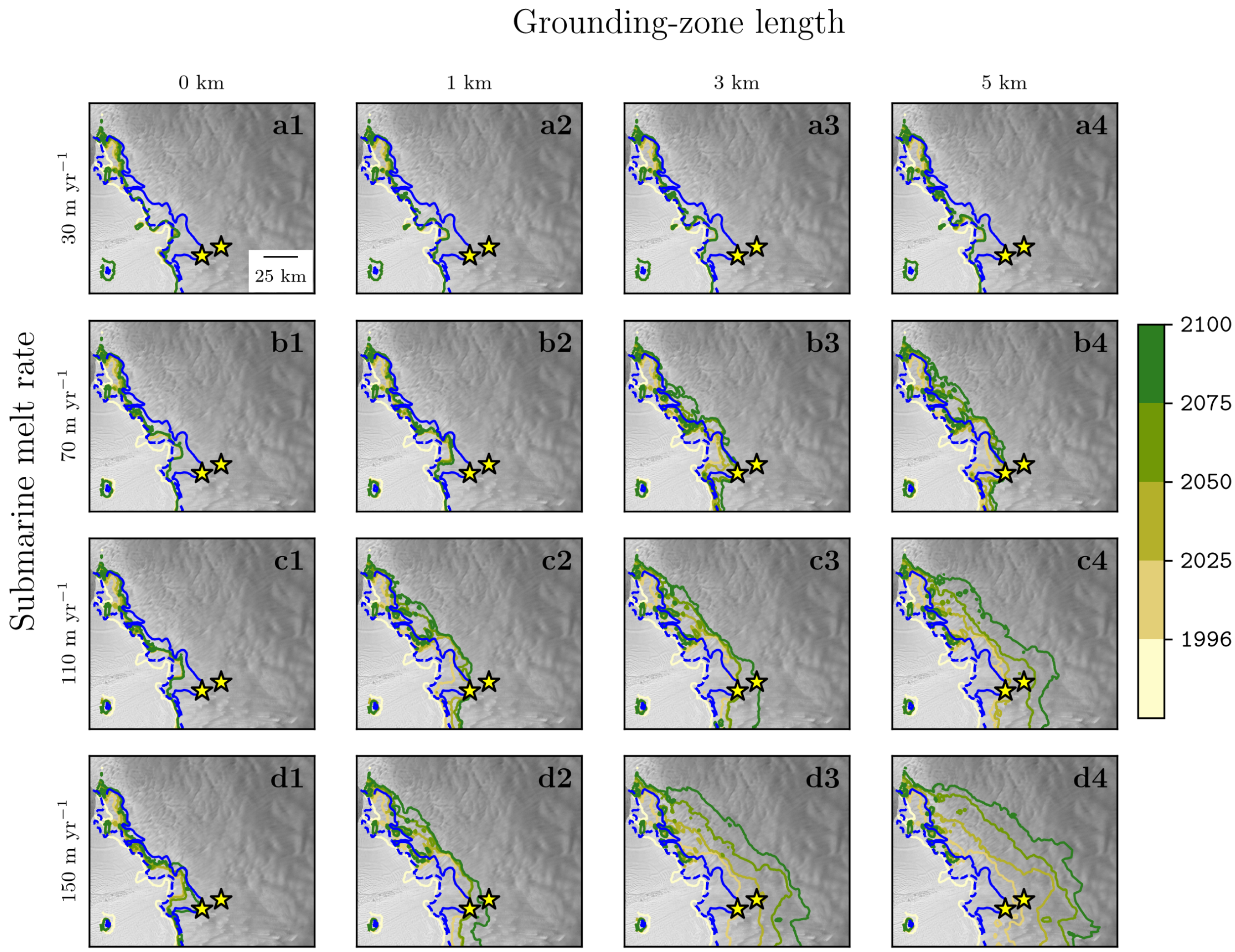

Supplementary Figure 7: Future grounding line retreat. Color-coded lines are the modeled grounding line location from 1996 to 2100. The dashed and solid blue lines are the grounding line locations observed in 1996 and 2023, respectively (*6*). Yellow stars are the location of Mouginot Ridge and South Ridge. Background is the RADARSAT-1 mosaic of Antarctica.

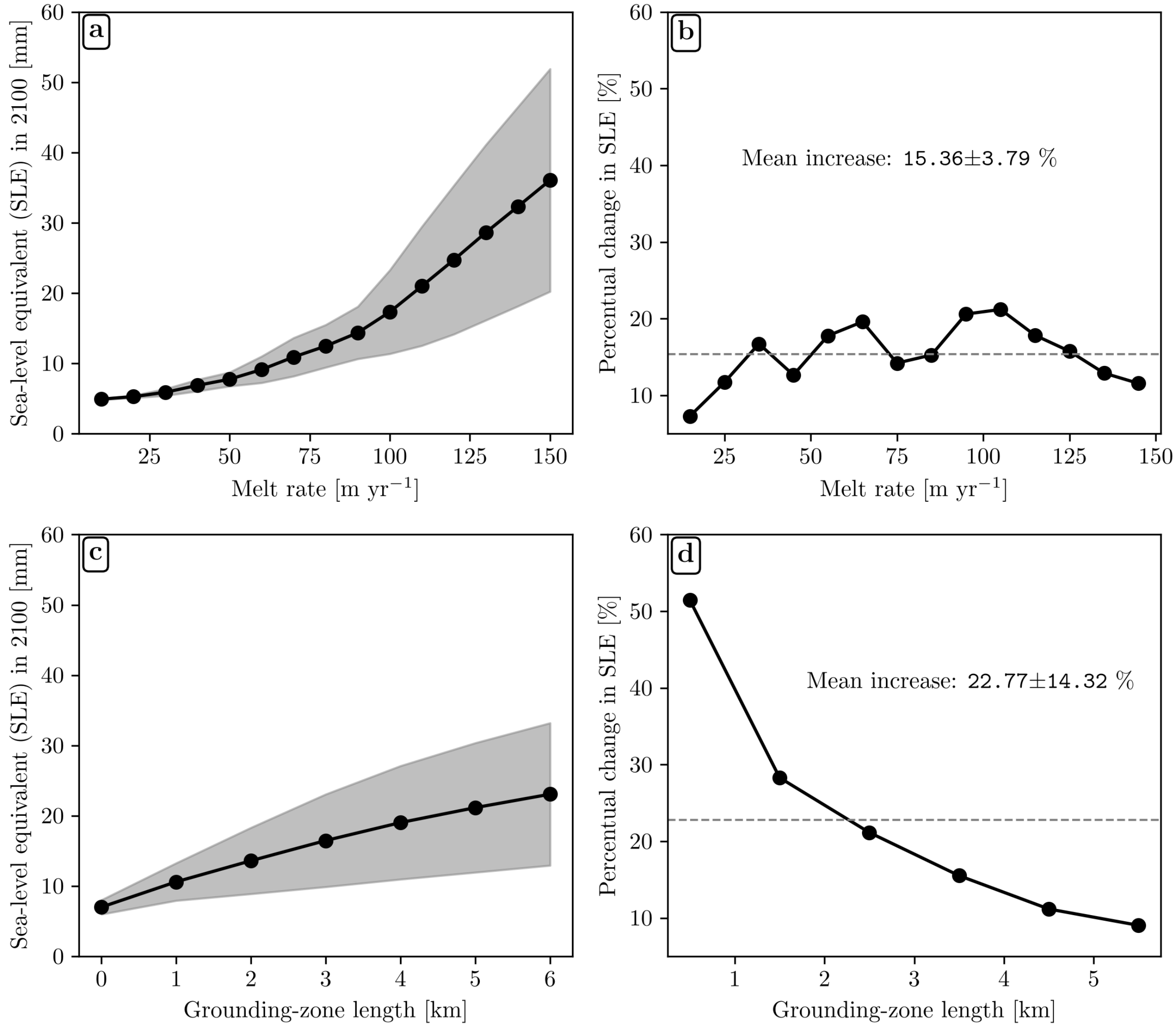

Supplementary Figure 8: Sea-level equivalent in 2100 as function of melt rate and grounding-zone length. a Sea-level equivalent (SLE) in 2100 for simulations with different melt rates. Black line is the average and shaded area is the standard deviation across simulations with different grounding-zone lengths. **b** Percentage change in SLE for consecutive 10 m yr^{-1} increases in basal melt rate. **c** Sea-level equivalent (SLE) in 2100 for simulations with different grounding-zone lengths. Black line is the average and shaded area is the standard deviation across simulations with different melt rates. **d** Percentage change in SLE for consecutive 1 km increases in grounding-zone length.

Supplementary Table 1. RMSE values for TEIS Retreat [km^2]. RMSE is the root-mean-square error between modeled results and observed grounding line retreat for TEIS, y =379±25 km^2 (*8*). Minimum RMSE is underlined.

	Grounding-zone length						
Melt Rate	0 km	1 km	2 km	3 km	4 km	5 km	6 km
10 m yr^{-1}	207.1	187.8	180.8	176.7	173.3	170.2	167.3
20 m yr^{-1}	199.3	179.7	172.2	167.3	162.8	158.6	154.9
30 m yr^{-1}	190.8	169.7	160.1	154.0	148.8	144.1	140.1
40 m yr^{-1}	182.3	158.6	147.7	140.0	133.7	129.1	125.0
50 m yr^{-1}	171.1	145.2	132.4	125.0	117.7	110.3	103.0
60 m yr^{-1}	160.2	119.2	106.9	98.7	91.4	82.8	75.7
70 m yr^{-1}	146.4	105.6	92.2	82.5	75.0	68.0	60.8
80 m yr^{-1}	134.6	96.9	82.1	72.9	63.0	51.7	36.4
90 m yr^{-1}	124.6	92.1	74.4	61.2	39.3	18.6	2.1
100 m yr^{-1}	117.5	83.7	65.5	34.7	3.9	21.1	40.5
110 m yr^{-1}	114.2	77.6	46.6	**1.3**	30.9	51.3	66.1
120 m yr^{-1}	111.6	72.0	17.1	30.1	54.4	73.0	88.9
130 m yr^{-1}	109.6	65.9	12.6	50.7	75.2	94.2	108.4
140 m yr^{-1}	107.8	52.6	35.0	69.0	94.3	110.3	124.4
150 m yr^{-1}	106.4	30.6	51.2	87.2	108.1	124.2	147.4

Supplementary Table 2. RMSE values for TMT retreat [km^2]. RMSE is the root-mean-square error between modeled results and observed grounding line retreat for TMT, y =567±25 km^2 (*8*). Minimum RMSE is underlined.

	Grounding-zone length						
Melt Rate	0 km	1 km	2 km	3 km	4 km	5 km	6 km
10 m yr^{-1}	552.2	545.7	542.3	540.3	538.8	537.7	536.6
20 m yr^{-1}	532.6	520.3	508.6	503.2	499.4	495.0	490.5
30 m yr^{-1}	511.4	473.6	456.6	448.4	442.6	437.5	432.3
40 m yr^{-1}	481.9	437.3	424.5	400.5	368.2	344.5	328.6
50 m yr^{-1}	443.0	404.2	340.7	312.1	297.0	285.9	276.9
60 m yr^{-1}	413.2	334.2	281.5	265.3	253.1	228.0	207.9
70 m yr^{-1}	397.5	277.1	248.3	217.3	193.7	162.7	133.5
80 m yr^{-1}	385.6	248.1	202.1	168.5	112.7	**2.5**	67.5
90 m yr^{-1}	375.4	223.5	161.7	49.8	79.3	169.5	235.3
100 m yr^{-1}	364.8	180.2	86.6	115.4	237.3	297.4	327.8
110 m yr^{-1}	354.7	146.4	89.6	258.5	329.9	371.6	407.4
120 m yr^{-1}	309.9	125.5	206.1	342.3	399.8	444.1	476.1
130 m yr^{-1}	279.1	42.2	318.4	407.3	465.1	497.7	509.4
140 m yr^{-1}	259.1	98.7	375.1	466.9	504.7	514.3	584.4
150 m yr^{-1}	244.1	189.8	429.9	504.4	517.0	591.8	848.8

Supplementary Table 3. RMSE values for glacier thinning [m]. RMSE is the root-mean-square error between modeled results and observed glacier thinning between 1996 and 2020, y =-12.84±0.92 m (*9*). Minimum RMSE is underlined.

	Grounding-zone length						
Melt Rate	0 km	1 km	2 km	3 km	4 km	5 km	6 km
10 m yr^{-1}	6.3	6.7	6.8	6.8	6.7	6.7	6.6
20 m yr^{-1}	6.1	6.4	6.4	6.4	6.3	6.2	6.1
30 m yr^{-1}	5.9	6.1	6.2	6.0	5.9	5.7	5.5
40 m yr^{-1}	5.7	5.9	5.8	5.7	5.6	5.4	5.2
50 m yr^{-1}	5.7	5.8	5.8	5.6	5.3	5.0	4.6
60 m yr^{-1}	5.7	5.9	5.7	5.3	4.9	4.4	4.0
70 m yr^{-1}	5.7	5.7	5.3	4.9	4.4	3.8	3.1
80 m yr^{-1}	5.7	5.5	5.0	4.4	3.7	2.9	1.8
90 m yr^{-1}	5.7	5.3	4.6	3.7	2.6	1.3	0.1
100 m yr^{-1}	5.7	5.1	4.0	2.7	1.1	0.3	1.5
110 m yr^{-1}	5.7	4.8	3.4	1.3	0.3	1.8	3.1
120 m yr^{-1}	5.7	4.4	2.2	**<u>0.0</u>**	1.8	3.3	4.6
130 m yr^{-1}	5.7	3.9	1.2	1.2	3.0	4.6	6.1
140 m yr^{-1}	5.7	3.5	0.2	2.3	4.3	6.0	7.6
150 m yr^{-1}	5.6	2.9	0.8	3.4	5.6	7.4	9.2

Supplementary Table 4. RMSE values for glacier speed-up [m yr^{-1}]. RMSE is the root-mean-square error between modeled results and the mean observed glacier speed-up along the along the fastest moving sector of Thwaites (dashed line in Fig. 1A), from 1996 to 2022, y =145±18.3 m yr^{-1} (*8*). Minimum RMSE is underlined.

	Grounding-zone length						
Melt Rate	0 km	1 km	2 km	3 km	4 km	5 km	6 km
10 m yr^{-1}	160.0	158.9	158.2	157.8	157.5	157.2	157.0
20 m yr^{-1}	155.4	152.7	149.9	148.7	147.8	146.8	145.8
30 m yr^{-1}	150.0	140.6	135.5	133.6	132.5	131.5	130.7
40 m yr^{-1}	142.8	130.5	128.4	126.9	125.2	123.5	121.7
50 m yr^{-1}	132.2	124.5	121.6	119.0	117.2	115.6	114.2
60 m yr^{-1}	125.6	118.6	113.7	111.2	109.6	106.7	104.1
70 m yr^{-1}	121.1	112.1	108.1	105.0	100.9	86.8	78.8
80 m yr^{-1}	118.2	107.5	102.6	96.1	73.3	**<u>15.5</u>**	35.2
90 m yr^{-1}	115.9	104.0	94.1	60.9	48.6	78.6	104.6
100 m yr^{-1}	113.3	99.3	70.9	71.8	114.9	146.3	158.2
110 m yr^{-1}	111.5	91.5	62.3	134.0	167.7	180.6	186.8
120 m yr^{-1}	109.6	85.7	119.9	180.0	193.4	200.3	208.3
130 m yr^{-1}	106.4	64.2	174.3	200.2	211.9	227.2	230.5
140 m yr^{-1}	104.0	27.6	200.6	215.6	236.2	233.7	247.2
150 m yr^{-1}	102.3	115.6	210.9	239.5	237.4	253.9	581.9

Supplementary Table 5: Tabulated sea-level contribution in 2100. Calculated sea-level equivalent [mm] of Thwaites glacier in 2100 from model simulations. Mean of the ISMIP6 ensemble (*10*) is 15.2±21.8 mm.

	Grounding-zone length						
Melt Rate	0 km	1 km	2 km	3 km	4 km	5 km	6 km
10 m yr^{-1}	4.8	4.9	4.9	4.9	5.0	5.0	5.0
20 m yr^{-1}	4.9	5.1	5.3	5.3	5.4	5.4	5.5
30 m yr^{-1}	5.2	5.5	5.6	5.8	6.2	6.4	6.6
40 m yr^{-1}	5.4	6.0	6.7	7.1	7.4	7.6	7.8
50 m yr^{-1}	5.7	7.1	7.7	8.0	8.2	8.5	8.9
60 m yr^{-1}	5.8	7.9	8.3	8.9	10.1	11.0	11.8
70 m yr^{-1}	6.0	8.4	9.9	11.7	12.8	13.5	14.0
80 m yr^{-1}	6.6	9.4	12.4	13.8	14.6	15.0	15.3
90 m yr^{-1}	7.3	11.6	14.1	15.1	15.5	16.8	20.0
100 m yr^{-1}	7.8	13.2	15.2	15.8	19.0	23.0	27.2
110 m yr^{-1}	8.3	14.3	15.8	19.4	25.2	30.2	33.8
120 m yr^{-1}	8.8	15.1	17.5	25.1	31.4	35.9	39.4
130 m yr^{-1}	9.1	15.7	22.1	30.7	36.5	41.2	45.2
140 m yr^{-1}	9.4	16.6	27.0	35.4	41.5	46.5	49.9
150 m yr^{-1}	9.9	18.1	31.4	39.9	46.5	51.2	55.4

References

1. J. L. Bamber, R. A. Bindschadler, An improved elevation dataset for climate and ice-sheet modelling: validation with satellite imagery. *Annals of Glaciology* **25**, 439–444 (1997).

2. E. Rignot, J. Mouginot, M. Morlighem, H. Seroussi, B. Scheuchl, Widespread, rapid grounding line retreat of Pine Island, Thwaites, Smith, and Kohler glaciers, West Antarctica, from 1992 to 2011. *Geophysical Research Letters* **41** (10), 3502–3509 (2014).

3. E. Rignot, *et al.*, Widespread seawater intrusions beneath the grounded ice of Thwaites Glacier, West Antarctica. *Proceedings of the National Academy of Sciences* **121** (22), e2404766121 (2024).

4. E. Rignot, *et al.*, Four decades of Antarctic ice sheet mass balance from 1979–2017. *Proceedings of the National Academy of Sciences of the United States of America* **116** (4), 1095–1103 (2019).

5. M. Morlighem, *et al.*, Deep glacial troughs and stabilizing ridges unveiled beneath the margins of the Antarctic ice sheet. *Nature Geoscience* pp. 1–6 (2019).

6. E. Rignot, B. Scheuchl, E. Ciraci, Thirty years of glacier grounding line retreat in Antarctica. *Proceedings of the National Academy of Science of the USA, under review* (2025).

7. A. M. Chartrand, I. M. Howat, I. R. Joughin, B. E. Smith, Thwaites Glacier thins and retreats fastest where ice-shelf channels intersect its grounding zone. *The Cryosphere* **18** (11), 4971–4992 (2024).

8. E. Rignot, J. Mouginot, B. Scheuchl, S. Jeong, Changes in Antarctic Ice Sheet Motion Derived From Satellite Radar Interferometry Between 1995 and 2022. *Geophysical Research Letters* **49** (23), e2022GL100141 (2022).

9. J. Nilsson, A. S. Gardner, F. S. Paolo, Elevation change of the Antarctic Ice Sheet: 1985 to 2020. *Earth System Science Data* **14** (8), 3573–3598 (2022).

10. H. Seroussi, *et al.*, Evolution of the Antarctic Ice Sheet Over the Next Three Centuries From an ISMIP6 Model Ensemble. *Earth's Future* **12** (9), e2024EF004561 (2024).
