# Supplementary material for: Sea-level contribution from Thwaites Glacier doubles in an observation-constrained ice-sheet model
Source: Nat Commun. 2026 Oct 12;17:10364. doi: 10.1038/s41467-026-77976-1 (PMC13658800; doi:10.1038/s41467-026-77976-1)
Supplement: Supplementary file 2 [file 41467_2026_77976_MOESM2_ESM.pdf]

nature portfolio

Peer Review File

Sea-level contribution from Thwaites Glacier doubles in an observation-constrained ice-sheet model.

Corresponding Author: Dr Mattia Poinelli

This file contains all reviewer reports in order by version, followed by all author rebuttals in order by version.

Attachments originally included by the reviewers as part of their assessment can be found at the end of this file.

Version 0:

Reviewer comments:

Reviewer #1

(Remarks to the Author)
Sea-level contribution from Thwaites Glacier doubles in an observation-constrained ice-sheet model
Poinelli, M., E. Rignot, H. Seroussi, J. Cuzzone, T. Pelle, and E. Larour

Submitted for publication in Nature Communications

General Comments

This data-constrained modeling study assesses the impact of ocean-driven melt in a glacier-wide, variable-length grounding zone on the evolution of the neighboring Thwaites and Haynes Glaciers within the Amundsen Sea Embayment of West Antarctica. The experimental design is appropriately targeted and suitably simplified, leading to results that are largely well supported and presented (there are a couple italicized specific comments below that, if addressed, would help strengthen the conclusions). This timely study represents an informative next-step in illustrating the importance of grounding-zone process inclusion in simulating past dynamics and improving projections of the cryospheric contributions to future eustatic sea level, which is of clear and broad societal relevance. It also provides an example of how continental-scale ice sheet models can effectively parameterize the dynamic impact of ice-ocean interactions inland of the grounding line while research continues into the many processes and feedbacks operating within this critical region of outlet glaciers.

As the authors importantly state, this remains a lower-bound scenario due [in part] to the “controlled, constant climate forcing”. Inasmuch, the study’s design and focus are squarely on isolating and highlighting the importance of various melt rates within variable-length grounding zones. The authors use Bayesian calibration to determine the most likely combinations of melt rate and grounding-zone length that successfully hindcast multiple dynamic targets for outlet glaciers (namely the grounding-line, ice-discharge, ice-thickness, and ice-speed evolutions), offer a method to simulate grounding zones within ice-sheet models, and intercompare a suite of their resulting sea-level projections against the ISMIP6 ensemble under similar constant forcing. Even without feedbacks associated with oceanic and/or atmospheric variability, they find that grounding-zone inclusion roughly doubles previously published results.

Specific Comments

A few specific points and edits to consider:

The ice domain includes both Thwaites Glacier and Haynes Glacier, yet most figures highlight the area of fastest retreat and discussions implicitly attribute the basin-wide changes to Thwaites Glacier alone. I assume Haynes Glacier is included in the bulk analyses of the model output. While changes on Thwaites are expected to dominate the signals, in the title and text, Haynes Glacier is all but omitted (other than it is indeed part of the simulations). In your revisions, please consider addressing the simulated evolution of Haynes Glacier, or at least why you opt to model it but not discuss it within your

analyses.

How is the basal friction coefficient, Cs, treated in the grounding-zone parameterization of this well-lubricated region? Is ocean incursion only influencing melt rates in these simulations or both basal melt and drag? To aid in the interpretation of the results, whether and how the coefficient is varied should be explicitly stated and justified within the Methods section and perhaps briefly included (and/or referenced) around Lines 73-77. See comment below regarding the inversions (Lines 252-254), as assuming the ocean incursion does/does not impact dynamic drag within the grounding zone can have leading effects on both the model initialization and prognostic outcomes.

Line 16: This is an inadvertently confusing placement of the citation 2 superscript. I would recommend shifting it, so it doesn't inadvertently look like it is changing the units for the reader:
of 65 cm^2, --> of^2 65 cm,
(consider making all similar changes elsewhere; e.g., Lines 71, 145, 146)

Line 17: where grounded ice become --> where grounded ice becomes

Line 18: in form of ice shelves --> in the form of ice shelves

Line 21: in the glacier central --> in the glacier's central

Line 74: Laboratory work and microseismicity indicate basal ice containing debris that slides over a bumpy bed leads to strain hardening, which would effectively remove an Iken limit. It would therefore be informative to know where and when the denominator in equation (1) is most strongly activated in these simulations (i.e., significantly differs from unity).

Zoet, L. K., Ikari, M. J., Alley, R. B., Marone, C., Anandakrishnan, S., Carpenter, B. M., & Scuderi, M. M. (2020)

Zoet, L., IGS Global Seminar Series, February 4, 2026.

Line 79+: Note that the impact of the length of seawater intrusions into the grounding zone on grounding-line retreat is also consistent with previous studies (see reference 21). This current study takes a large and important step forward by using far more target data to constrain the combinations of melt rate and grounding-line length. As found in21, and further supported here, given Thwaites' rugged, stabilizing basal topographic highs across which it flows to reach the Amundsen Sea, grounding-zone melt most effectively removes the locally provided basal drag once the length of the grounding zone nearly matches or exceeds the characteristic length scales of many of these ridges. This reduces the time spent grounded on these stabilizing points and, combined with MISI and the regularized Coulomb treatment of the basal rheology, ultimately leads to enhanced retreat rates. That is why it takes a higher melt rate when the grounding zone is shorter to peel back the grounding line in a manner that matches the observed retreat rates. Thinning to floatation is more localized while drag remains high just upstream on the same bump, so the torch needs to be hotter to better match the integrated mass loss. Perhaps worth adding a statement about this relationship between the typical length scales of the ridges, the simulated grounding-zone lengths, and the resulting retreat rates to your discussion. (Also see supporting concluding statement on Lines 130-131.)

Lines 87-92: These three sentences need to be reworded for clarity. Also note minor edit:
results still underestimates --> results still underestimate

Line 98: Fix units, 100 m yr^{-1} --> 100 Gt yr^{-1}

Line 100 (and elsewhere; e.g., Line 336-340): When referring to glacier thinning (or speeds) in the manuscript, please make it clear where the comparisons to data and/or reported output values are being made in the model domain. There is a lot of "grounded ice" in this ASE domain, and even within the embayment-wide grounding zone or along the dashed flow-line across the fastest flowing sector of Thwaites. Is this a nodal value through time or an average value (over which region) through time?

Line 119 and Fig. 1 caption: Is "South Ridge" an extension of the ITGC's GHOST Ridge? If it is, it would be helpful to the community to include an (a.k.a., GHOST Ridge). (Though, GHOST Ridge might be another 10-km or so upglacier.) Whether distinct or not, note that the name is then changed to "Ridge South" in Extended Data Fig. 1, Extended Data Fig. 4, and Supplementary Figure 3.

Line 123: consistently with their fourfold --> consistent with their fourfold

Line 140-141: Indeed, but your calibrated results also clearly indicate how important process inclusion can be to projected outcomes. Not only do their omission risk skewing the results, but also the attribution. Taken together, this can lead to progressively less reliable predictions as well as more collective misunderstanding. You highlight these critical points later in Lines 195-204, but might want to consider a word or two here as well to help build the import of your results as opposed to holding off and including an apparent leap at the very end of the manuscript.

Line 153: There is a reference to Fig. S6A, which doesn't appear to exist in my downloaded version of the manuscript. The reference to Fig. 4A holds.

Line 165: as well as glacier migration --> as well as grounding-line migration
(Note, although some might only consider grounded ice, an ice shelf is also part of a glacier. Therefore, strictly speaking, with a fixed ice front, the simulated glacier never migrates. As your historical data focus is on the grounding-line evolution here, not that of the ice front, I would recommend rewording as above to remove any potential confusion.)

Lines 195-199: On first read, there was a lot of repetition as "kilometer-scale" is included twice in succession and "actively-melting grounding zones" is repeated three times. I'm assuming this was intentional, but perhaps consider rewording (unless you feel strongly that this was done with purpose). Also, "actively-melting" should indeed be hyphenated all three times.

Lines 232-235: Is adaptive mesh refinement included? Given the number of simulations in the ensemble, I'm assuming not. However, if the mesh is static, please include a statement on how close the most retreated grounding lines in the 100-year suite of experiments are to the transition between 500-m and 20-km nodal spacings, as that can numerically impact your results.

Lines 252-254: As the surface-velocity misfit colorbar is saturated, it would be useful to also include max and min values of the misfits in the Methods text. Given this study is assessing grounding-zone impacts on ice dynamics, please include a statement/discussion of the likely impact of the grounding-zone proximal misfits on the overall results.
(i.e., The experiments start with inversion-assigned values to ice hardness and basal drag that appear to be overly suppressing the ice flow in this critical region to best match the speeds here as well as elsewhere in the domain. Then you apply melt in this grounding-zone region, which, in time, ultimately turns off the basal drag there that was contributing to the initially slower-than-observed ice speeds once the ice goes afloat (as the grounding line retreats). In doing so, you have removed the somewhat artificial speed limiter (as flagged by the misfit) and therefore one might expect a potentially-more-drastic loss of ice volume above floatation (heightened contribution to sea level) than would occur if the inverted values for friction coefficients were lower there, and instead the momentum-balancing drag was distributed more widely to improve the initial misfits within the very zone on which this modeling effort is focused. Also, please see the italicized comment above pertaining to C_s in the grounding zone.)

Line 258: remove indent before "where C_s" just after equation (1)

Line 258: A word on how the effective pressure is calculated is this study (under what assumptions), whether it is a static value or evolving, and whether the parameterization of the grounding zone includes an ocean-incursion impact on N there (or alternatively, an impact on C_s, as inquired above) would be helpful to the interpretation of results. Please note, at the very least, the limitations and potential impact of these various modeling assumptions should be acknowledged for the reader.

Line 265: remove "a" as there are two fields here;
to obtain a three-dimensional velocity and pressure fields --> to obtain three-dimensional velocity and pressure fields

Line 269: Please note missing ref; simulating seawater intrusions with a linear ramp in basal melt builds on 21, 22, and 25.

Line 281: Rather than stopping at 6 years, what happens after 8, 10, or 12 spin-up years if a constant SMB is maintained (Extended Data Fig 1B and Supp Fig 3B)? The downward drift in ice discharge between years 5 and 6 mirrors the upward drift between years 4 and 5. If the drift continues as a mirrored image, subsequent spin-up years might fall outside the target standard deviation range around the data. If that is the case, consider what impact starting with a drifting IC has on your results. On first take, it might mean having to kick the system a bit harder to overcome the diminishing ice discharge and reproduce the hindcasts. The larger grounding-zone and/or melt-rate forcing required to overcome the spin-up drift would then also lead to increased sea-level rise projections. Nature presents us with glaciers that are never truly in steady state with their ice-ocean-atmosphere-solid Earth-substrate system, so having a drifting starting point is quite reasonable. However, the chosen spin-up duration and then starting the experiments with the existing drift are modeling choices as opposed to fits with temporal trends in the data. A discussion regarding the impact of these choices on the results is therefore warranted.

Lines 296-297: I went back and forth with this one. As worded, the referenced studies did indeed indicate what is stated here. However, the preconditioning processes that can ultimately lead to calving as well as the very existence of an ice shelf (even if freely spreading) matter to Thwaites' past and future dynamics. If this sector continues to flow along as it has during the satellite era, "unlikely to be the dominant driver […] under current conditions" might suffice here.
However, there is healthy, ongoing scientific debate on this point and on Thwaites' dynamic trajectory. The challenge is deciding whether it is best to support your modeling choices with the dependent clause "although recent studies…" or simply to remind the reader that "It is important to note that the last metric to assess the full state of the glacier is the location of the ice front, as calving could lead to buttress removal and glacier acceleration."
Whichever you decide, I would recommend rephrasing "last metric" to "last readily-observable metric" as these are all metrics that can be directly compared against the existing satellite record, but certainly not the only metrics that control the full state of the glacier.

Line 328: Beyond stating that it serves as a normalization constant, it might be helpful to also briefly explain how the marginal likelihood is determined. As these are discrete parameter values for melt rate and grounding zone length that are being tested, wouldn't it simply be the sum of all tested combinations comprising the product in the numerator of (3)? If so, shouldn't Extended Data Fig. 3 look like discrete bar charts (probability mass functions) as opposed to continuous posterior distribution functions? Or perhaps, because of the linear interpolation and large sampling, you have generated enough

discrete values to approximate them as continuous functions? Either way, please include a clarifying statement.

References: Note many titles are missing capitalization of place names. e.g., thwaites west antarctica, etc.

Extended Data Fig. 3 caption: For improved readability, consider including commas, a semicolon, "In", and "in"--> "employing the parameter, θ_i, to reproduce the data, y. In (A), θ_i is melt rate; in (B)θ_i is grounding zone length."

Extended Data Fig. 5: For quick visual comparison and to help with the interpretation of the relative impact of the two parameters, it would be helpful if plots A and C had the same ordinate axis limits, and again for plots B and D.

Supplementary Figure 2 C: missing units on the basal friction coefficient, C_s

Supplementary Figure 2 caption: to be consistent with Eqn 1:
friction coefficient C on grounded ice --> friction coefficient C_s on grounded ice

Congratulations on this targeted and important contribution in advancing our understanding of the impact of grounding zones on ice dynamics, as supported by data, physical understanding, and theoretical reasoning.

(Remarks on code availability)

Reviewer #2

(Remarks to the Author)
Please see attached document.

(Remarks on code availability)
The title of the repository does not match the current manuscript.
The file description on the repository says there is a 'step 16' to reproduce the sensitivity simulations in the paper, but I cannot find this.
The repository also mentions step 11 in the 'readme.m' but this doesn't seem to exist.
I could not load the mat file as intended by the author (as a 'model' format), error is: "Warning: Variable 'md' originally saved as a model cannot be instantiated as an object and will be read in as a uint32."
I could not run the Matlab script, I got the error: Unrecognized function or variable 'organizer'.

Version 1:

Reviewer comments:

Reviewer #1

(Remarks to the Author)
Sea-level contribution from Thwaites Glacier doubles in an observation-constrained ice-sheet model
Poinelli, M., E. Rignot, H. Seroussi, J. Cuzzone, T. Pelle, and E. Larour

Revised for publication in Nature Communications

General Comments

The authors did a fantastic job addressing reviewer concerns from the first round. Congratulations on your important, carefully-planned, and well-presented research contribution. Thank you for your thorough revision efforts.

As this is my second review, I have a few minor points to consider.

Specific Comments

Fig. 1 caption: I realize this is in direct conflict with Ref. 2's first round of comments, but I see blue, dark brown, and light brown on my image per the original caption. I do not see "grey" elements. Please triple check this.

Line 106: calculated as spatial average --> calculated as a spatial average

Line 156 and Supplementary Fig. 8 d): If I'm interpreting Supp Fig 8 correctly, both panels (a) and (c) include the full suite of experiments (all grounding zone lengths, L, in (a) and all melt rates in (c)). What (b) and (c) are illustrating is that i) for melting rates, above about 40 m/yr, additional melting still leads to more SLE, but at a similar % increase for each 10 m/yr jump,

whereas ii) for L, the biggest SLE % change occurs by simply introducing a grounding zone; thereafter, while SLE still rises with increasing grounding zone length, the rate of SLE change diminishes with each added km. Nevertheless, the mean increase in % SLE change is higher for L changes than it is for basal melting rate changes. If this is all correct, then shouldn't the text in the Fig. 8 panel d state "Mean increase", not "Mean decrease", despite the downward trend in % SLE change with increasing L?

Line 216: extra period

Line 229: M.P. build model domain and run the sensitivity study --> M.P. built the model domain and ran the sensitivity study

Line 269: to remove any ambiguity between ice bed and seafloor consider either:
The elevation of the bed is increased --> the elevation of the seabed is increased -or- the bathymetry is increased

Line 270: with bed elevation of approximately 1000 m. --> with seafloor depth of approximately -1000 m.

Line 274: Best to remain consistent throughout the manuscript with ice stiffness, ice hardness, and rheology, all being used interchangeably for the same parameter, B, which is often referred to as the ice hardness parameter. I would recommend against calling B the ice rheology, as this is a temperature dependent parameter within the flow law that governs the rheology of ice that is implemented. While indeed part of the rheological equation, the power, n, is more influential in specifying the simulated "power-law" ice rheology, just as m is more influential than the basal friction coefficient in specifying the simulated basal rheology.
We derive ice stiffness for floating ice --> We derive the ice hardness parameter for floating ice -or- We derive the ice hardness for floating ice

Lines 280-281, 285-287: Though you use a regularized Coulomb friction law that depends on N, you are not including a water system (stated on Line 194; helpful to reiterate here). You then elect to calculate the effective pressure as equal to the evolving ice overburden pressure everywhere for THW (i.e., you are thereby assuming zero water pressure, which equates to either a frozen bed, highly efficient infiltration into underlying aquifers, or an extremely efficient, channelized basal water system to help dewater most of the bed). This simplifying assumption, $N \dashrightarrow P_i$, does not match existing data for most of THW, other than near channelized systems along the margins, around bedrock highs and fluted bedforms, and/or perhaps proximal to the grounding zone to help efficiently feed subglacial water to the ocean (until the point where ocean influences reach upglacier into the grounding zone). Furthermore, isn't $N = P_i$ directly contrary to the Iken assumption of a perfectly lubricated bed, which is the essence of that particular component of the Coulomb friction law? Based on your careful post-processing analysis to address another review question about where the Iken limit is activated in the model domain versus sliding following an m=3 power-law, you now report that Coulomb sliding only kicks in across the fastest-flowing regions, just upstream of the grounding line. Therefore, while $P_w = 0$ Pa does not match data nor the conditions for Coulomb sliding and ocean incursion, given that Coulomb sliding was only activated in regions we might anticipate are ripe for reduced dynamic drag, the choice of this sliding law might have fortuitously helped partially compensate for the static and high basal friction coefficient field across this region. Inasmuch, the targeted sensitivity results and discussions remain reasonable under your stated modeling assumptions. However, for the benefit of the wider readership, please consider including an additional comment regarding the known oversimplification of assigning $N = P_i$ when simulating THW. In that discussion, you might point out that, fortunately, across most of the natural domain where $P_w \dashrightarrow P_i$ and therefore effective pressures are actually low, a power-law bed rheology dominates your simulated sliding and therefore the assignment of overburden pressures everywhere is essentially ignored.

Line 291: As above
To retrieve the rheology of grounded ice --> To retrieve the ice hardness of grounded ice

Line 296: As above
To inform the ice rheology --> to inform the ice hardness

Line 325: As above
To this end, the bed correction --> To this end, the seabed correction -or- To this end, the bathymetric correction

Lines 334-336 and again on Lines 344-345: Please check your output to make sure the drag on the pinning points remained unchanged throughout the simulations. With enhanced flux across the grounding line and the highest melt rates concentrated near the grounding line, there is significant potential to change the drag across the far-field pinning points. Changes in flux lead to thickness propagations across the ice shelf and concentrating melt at depth effectively planes off ice layers that would otherwise be advected across all remaining down-glacier sites. (i.e., Basal melt need not be proximal to the pinning point to impact the buttressing it offers.) Even if the simulated drag happens to remain unchanged on the pinning points, the wording here needs to be changed because of these potential dynamic feedbacks.

Supplementary Figure 4 caption: As above
Implications of the 100 m bed correction --> Implications of the 100 m seabed correction

with original bed topography --> with original seabed topography -or- with original bathymetry

Again, well done and thank you for your contribution.

(Remarks on code availability)

Reviewer #2

(Remarks to the Author)
Having read the revised manuscript from Poinelli et al., I am pleased to see that all of my suggestions have been taken into account. In particular, the inclusion of statements regarding uncertainty in initial geometry and limitations with the model setup. I am satisfied that all of my previous comments have been addressed and will be happy to see the manuscript published.

(Remarks on code availability)

Open Access This Peer Review File is licensed under a Creative Commons Attribution 4.0 International License, which permits use, sharing, adaptation, distribution and reproduction in any medium or format, as long as you give appropriate credit to the original author(s) and the source, provide a link to the Creative Commons license, and indicate if changes were made.
In cases where reviewers are anonymous, credit should be given to 'Anonymous Referee' and the source.
The images or other third party material in this Peer Review File are included in the article’s Creative Commons license, unless indicated otherwise in a credit line to the material. If material is not included in the article’s Creative Commons license and your intended use is not permitted by statutory regulation or exceeds the permitted use, you will need to obtain permission directly from the copyright holder.
To view a copy of this license, visit https://creativecommons.org/licenses/by/4.0/

Manuscript: NCOMMS-26-033269-T

Title: Sea-level contribution from Thwaites Glacier doubles in an observation-constrained ice-sheet model

Authors: Mattia Poinelli, Eric Rignot, Hélène Seroussi, Josh Cuzzone, Tyler Pelle, Eric Larour

A Response to the anonymous reviewers

First of all, we would like to thank the Editor and the two anonymous reviewers of *Nature Communications* for their constructive comments and suggestions. The manuscript has undergone a thorough revision according to the editor and reviewers' comments, which, in our opinion, have helped improve both quality and impact of this manuscript. Furthermore, we have substantially revised the structure of the manuscript in order to agree with the formatting standards of *Nature Communications*, per editor request. In particular, we have moved all extended Data Figures and Tables in the Supplementary information.

Please find our responses to the two anonymous reviewers below to this point. For the reviewers' and editor's convenience, we have highlighted our responses to the comments in blue. Moreover, we have attached a tracked-changes version to highlight our edits with respect to the original version of the manuscript, which are also in blue. Please note that when we mention specific lines in the response to reviewers, we are referring to lines number in the tracked changes version of the manuscript.

Contents

A Response to the anonymous reviewers **1**
A.1 Response to reviewer 1 2
A.2 Response to reviewer 2 9

A.1 Response to reviewer 1

Reviewer Comment 1.1 — General Comments

This data-constrained modeling study assesses the impact of ocean-driven melt in a glacier-wide, variable-length grounding zone on the evolution of the neighboring Thwaites and Haynes Glaciers within the Amundsen Sea Embayment of West Antarctica. The experimental design is appropriately targeted and suitably simplified, leading to results that are largely well supported and presented (there are a couple italicized specific comments below that, if addressed, would help strengthen the conclusions). This timely study represents an informative next-step in illustrating the importance of grounding-zone process inclusion in simulating past dynamics and improving projections of the cryospheric contributions to future eustatic sea level, which is of clear and broad societal relevance. It also provides an example of how continental-scale ice sheet models can effectively parameterize the dynamic impact of ice-ocean interactions inland of the grounding line while research continues into the many processes and feedbacks operating within this critical region of outlet glaciers.

As the authors importantly state, this remains a lower-bound scenario due [in part] to the "controlled, constant climate forcing". Inasmuch, the study's design and focus are squarely on isolating and highlighting the importance of various melt rates within variable-length grounding zones. The authors use Bayesian calibration to determine the most likely combinations of melt rate and grounding-zone length that successfully hindcast multiple dynamic targets for outlet glaciers (namely the grounding-line, ice-discharge, ice-thickness, and ice-speed evolutions), offer a method to simulate grounding zones within ice-sheet models, and intercompare a suite of their resulting sea-level projections against the ISMIP6 ensemble under similar constant forcing. Even without feedbacks associated with oceanic and/or atmospheric variability, they find that grounding-zone inclusion roughly doubles previously published results.

Reply: Thank you! We appreciate the effort of the reviewer in addressing our work. We are happy to hear that they find the study interesting and timely. Please find a detailed response to your comments and feedback below to this point.

Reviewer Comment 1.2 — Specific Comments. A few specific points and edits to consider:

The ice domain includes both Thwaites Glacier and Haynes Glacier, yet most figures highlight the area of fastest retreat and discussions implicitly attribute the basin-wide changes to Thwaites Glacier alone. I assume Haynes Glacier is included in the bulk analyses of the model output. While changes on Thwaites are expected to dominate the signals, in the title and text, Haynes Glacier is all but omitted (other than it is indeed part of the simulations). In your revisions, please consider addressing the simulated evolution of Haynes Glacier, or at least why you opt to model it but not discuss it within your analyses.

Reply: We include Haynes Glacier within the model domain because it is dynamically connected to Thwaites Glacier along the western boundary, and the two share a continuous ice trunk. This inclusion is necessary to accurately simulate the evolution of Thwaites Glacier. However, our analysis focuses exclusively on Thwaites Glacier, as it does dominate the observed ice-loss signal. Consequently, the dynamics of Haynes Glacier are not examined in detail here. We now specifically mention this limitation in the 'Ice-sheet model configuration' section (Line 270).

Reviewer Comment 1.3 — How is the basal friction coefficient, Cs, treated in the grounding-zone parameterization of this well-lubricated region? Is ocean incursion only influencing melt rates in these simulations or both basal melt and drag? To aid in the interpretation of the results, whether and how the coefficient is varied should be explicitly stated and justified within the Methods section and perhaps briefly

included (and/or referenced) around Lines 73-77. See comment below regarding the inversions (Lines 252-254), as assuming the ocean incursion does/does not impact dynamic drag within the grounding zone can have leading effects on both the model initialization and prognostic outcomes.

Reply: We agree with the reviewer that the basal drag at the grounding zone is an important factor to consider. In our model configuration, and in the new grounding zone module in ISSM, we only introduce melting below grounded ice using a linearly decay law as proposed in Robel et al 2022. We do not explicitly account for the spatial evolution of the basal friction coefficient in the grounding zone. This is an important aspect of the system, and one possible approach would be to prescribe a linear decay of the friction coefficient to zero across the grounding zone, representing the progressively lubricated region, as suggested by the reviewer. We have added a sentence in the Results (around line 80) and another in the Methods section (around line 338) to acknowledge this limitation and discuss it explicitly.

Reviewer Comment 1.4 — Line 16: This is an inadvertently confusing placement of the citation 2 superscript. I would recommend shifting it, so it doesn't inadvertently look like it is changing the units for the reader: of 65 cm^2, $-->$ of[2] 65 cm, (consider making all similar changes elsewhere; e.g., Lines 71, 145, 146)

Reply: Thanks for pointing out the confusion with this placement of citations. We have changed the location of the reference.

Reviewer Comment 1.5 — Line 17: where grounded ice become $-->$ where grounded ice becomes

Reviewer Comment 1.6 — Line 18: in form of ice shelves $-->$ in the form of ice shelves

Reviewer Comment 1.7 — Line 21: in the glacier central $-->$ in the glacier's central

Reply: Done.

Reviewer Comment 1.8 — Line 74: Laboratory work and microseismicity indicate basal ice containing debris that slides over a bumpy bed leads to strain hardening, which would effectively remove an Iken limit. It would therefore be informative to know where and when the denominator in equation (1) is most strongly activated in these simulations (i.e., significantly differs from unity).

Zoet, L. K., Ikari, M. J., Alley, R. B., Marone, C., Anandakrishnan, S., Carpenter, B. M., Scuderi, M. M. (2020)

Zoet, L., IGS Global Seminar Series, February 4, 2026.

Reply: We agree that debris-rich basal ice sliding over a rough bed may exhibit strain hardening, potentially reducing or eliminating the applicability of an Iken limit under some conditions. To place the role of the regularized Coulomb scheme in the context of our simulations, we evaluated the argument of the denominator in Eq. (1), i.e. $C_s|\mathbf{u}_b|/(C_{\max}N)$, throughout the model domain, as suggested by the reviewer. As shown in Review Figure 1 below, the regularization is active only in the fastest-flowing regions immediately upstream of the grounding line, where the argument reaches values of order 10^2. Elsewhere over the grounded ice sheet, the argument remains close to zero and the denominator is effectively unity, such that the friction law behaves similarly to the underlying power-law relation. Therefore, if strain hardening were to weaken or remove the Iken limit in some regions, our results indicate that this would primarily affect areas adjacent to the grounding line. We have added a sentence in the 'Ice-sheet model configuration' section around line 314 to clarify where the Iken-bound regularization is active.

Reviewer Comment 1.9 — Line 79+: Note that the impact of the length of seawater intrusions into the grounding zone on grounding-line retreat is also consistent with previous studies (see reference 21).

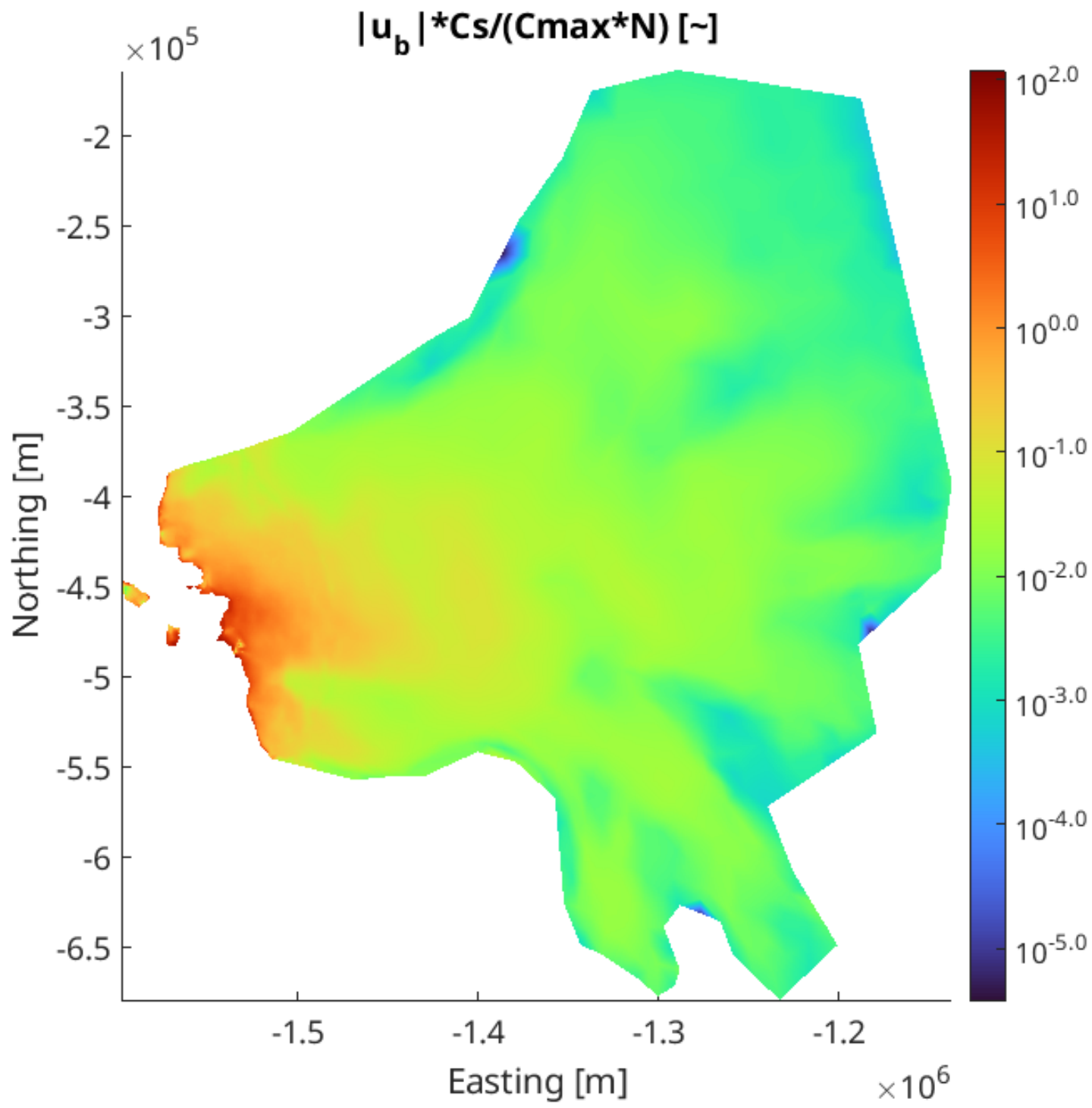

Review Figure 1: Denominator of Equation 1. The regularization is active only in the fastest-flowing regions near the grounding line, i.e. the denominator reaches values of order 10^2.

This current study takes a large and important step forward by using far more target data to constrain the combinations of melt rate and grounding-line length. As found in 21, and further supported here, given Thwaites' rugged, stabilizing basal topographic highs across which it flows to reach the Amundsen Sea, grounding-zone melt most effectively removes the locally provided basal drag once the length of the grounding zone nearly matches or exceeds the characteristic length scales of many of these ridges. This reduces the time spent grounded on these stabilizing points and, combined with MISI and the regularized Coulomb treatment of the basal rheology, ultimately leads to enhanced retreat rates. That is why it takes a higher melt rate when the grounding zone is shorter to peel back the grounding line in a manner that matches the observed retreat rates. Thinning to floatation is more localized while drag remains high just upstream on the same bump, so the torch needs to be hotter to better match the integrated mass loss. Perhaps worth adding a statement about this relationship between the typical length scales of the ridges, the simulated grounding-zone lengths, and the resulting retreat rates to your discussion. (Also see supporting concluding statement on Lines 130-131.)

Reply: Thank you for the suggestion. We find that the reviewer's narrative is a clear and elegant way to describe groundining-zone processes occuring over stabilizing ridges. We have added a brief discussion of the relationship between the grounding-zone width and ridge size around line 87, and we revisit this point later in the Results , around line 146.

Reviewer Comment 1.10 — Lines 87-92: These three sentences need to be reworded for clarity. Also note minor edit: results still underestimates −− > results still underestimate

Reply: Thank you! We have revised these sentences for clarity.

Reviewer Comment 1.11 — Line 98: Fix units, 100 m yr-1 −− > 100 Gt yr-1

Reply: Done.

Reviewer Comment 1.12 — Line 100 (and elsewhere; e.g., Line 336-340): When referring to glacier thinning (or speeds) in the manuscript, please make it clear where the comparisons to data and/or reported output values are being made in the model domain. There is a lot of "grounded ice" in this ASE domain, and even within the embayment-wide grounding zone or along the dashed flow-line across the fastest flowing sector of Thwaites. Is this a nodal value through time or an average value (over which region) through time?

Reply: Thanks for pointing out this confusion. Ice thinning is calculated as spatial average over grounded ice in the model domain while ice speedup as the difference between velocity in 2023 and 1996. We now mention this explicitely in the text around line 113.

Reviewer Comment 1.13 — Line 119 and Fig. 1 caption: Is "South Ridge" an extension of the ITGC's GHOST Ridge? If it is, it would be helpful to the community to include an (a.k.a., GHOST Ridge). (Though, GHOST Ridge might be another 10-km or so upglacier.) Whether distinct or not, note that the name is then changed to "Ridge South" in Extended Data Fig. 1, Extended Data Fig. 4, and Supplementary Figure 3.

Reply: The GHOST Ridge is located much farther inland (approximately 70 km from the present-day grounding line; Shwans et al., 2023, Journal of Glaciology). Since our simulations indicate that the Thwaites Glacier grounding line does not reach the GHOST Ridge within the simulated period, and is not expected to do so for at least several hundred years, we do not discuss it in the manuscript. We have, however, corrected the ridge names to South Ridge and Mouginot Ridge in all figures.

Reviewer Comment 1.14 — Line 123: consistently with their fourfold −− > consistent with their fourfold

Reply: Done.

Reviewer Comment 1.15 — Line 140-141: Indeed, but your calibrated results also clearly indicate how important process inclusion can be to projected outcomes. Not only do their omission risk skewing the results, but also the attribution. Taken together, this can lead to progressively less reliable predictions as well as more collective misunderstanding. You highlight these critical points later in Lines 195-204, but might want to consider a word or two here as well to help build the import of your results as opposed to holding off and including an apparent leap at the very end of the manuscript.

Reply: Done. We now introduce this point earlier in the manuscript (around line 158) so that it does not come as a surprise to the reader later in the text.

Reviewer Comment 1.16 — Line 153: There is a reference to Fig. S6A, which doesn't appear to exist in my downloaded version of the manuscript. The reference to Fig. 4A holds.

Reply: Thanks for spotting it. We have removed the reference as this was a figure generated for an old version of the manuscript.

Reviewer Comment 1.17 — Line 165: as well as glacier migration −− > as well as grounding-line migration (Note, although some might only consider grounded ice, an ice shelf is also part of a glacier. Therefore, strictly speaking, with a fixed ice front, the simulated glacier never migrates. As your historical data focus is on the grounding-line evolution here, not that of the ice front, I would recommend rewording as above to remove any potential confusion.)

Reply: Good point. We have revised this sentence and others in the manuscript when referring to grounding-line migration. As our ice front is fixed, grounding-line migration is what we mean.

Reviewer Comment 1.18 — Lines 195-199: On first read, there was a lot of repetition as "kilometer-scale" is included twice in succession and "actively-melting grounding zones" is repeated three times. I'm assuming this was intentional, but perhaps consider rewording (unless you feel strongly that this was done with purpose). Also, "actively-melting" should indeed be hyphenated all three times.

Reply: We have substantially revised these sentences to remove repetitions.

Reviewer Comment 1.19 — Lines 232-235: Is adaptive mesh refinement included? Given the number of simulations in the ensemble, I'm assuming not. However, if the mesh is static, please include a statement on how close the most retreated grounding lines in the 100-year suite of experiments are to the transition between 500-m and 20-km nodal spacings, as that can numerically impact your results.

Reply: Adaptive mesh refinement is enabled during the model initialization, but the mesh remains fixed throughout the transient simulations. During the initialization, we ensured that the expected grounding-line retreat over the 100-year simulation period is well resolved by a mesh with a resolution of 500 m. We now clarify this point in the model description around line 277.

Reviewer Comment 1.20 — Lines 252-254: As the surface-velocity misfit colorbar is saturated, it would be useful to also include max and min values of the misfits in the Methods text. Given this study is assessing grounding-zone impacts on ice dynamics, please include a statement/discussion of the likely impact of the grounding-zone proximal misfits on the overall results. (i.e., The experiments start with inversion-assigned values to ice hardness and basal drag that appear to be overly suppressing the ice flow in this critical region to best match the speeds here as well as elsewhere in the domain. Then you apply melt in this grounding-zone region, which, in time, ultimately turns off the basal drag there that was contributing to the initially slower-than-observed ice speeds once the ice goes afloat (as the grounding line retreats). In doing so, you have removed the somewhat artificial speed limiter (as flagged by the misfit) and therefore one might expect a potentially-more-drastic loss of ice volume above floatation (heightened contribution to sea level) than would occur if the inverted values for friction coefficients were lower there, and instead the momentum-balancing drag was distributed more widely to improve the initial misfits within the very zone on which this modeling effort is focused. Also, please see the italicized comment above pertaining to Cs in the grounding zone.)

Reply: We have included the maximum velocity misfit in the Methodology section (approximately 800 m yr^{-1} over the floating ice). That is a correct assumption of our treatement of high-friction areas immediately inland of the grounding zone and we now mention this limitation in the text, around line 82. Furthermore, we now explicitly discuss the lack of a proper treatment of the basal friction coefficient (around 338). As the reviewer points out, this is an important limitation of the study, and we now acknowledge it explicitly in the text.

Reviewer Comment 1.21 — Line 258: remove indent before "where Cs" just after equation (1)

Reply: Done.

Reviewer Comment 1.22 — Line 258: A word on how the effective pressure is calculated is this study (under what assumptions), whether it is a static value or evolving, and whether the parameterization of the grounding zone includes an ocean-incursion impact on N there (or alternatively, an impact on Cs, as inquired above) would be helpful to the interpretation of results. Please note, at the very least, the limitations and potential impact of these various modeling assumptions should be acknowledged for the reader.

Reply: Thank you for pointing out this missing aspect in the description of the model. The effective pressure is simply computed as

$$N = \rho_{\text{ice}} * g * H \tag{1}$$

where (H) is the ice thickness, corresponding to the overburden pressure of the ice column. At present, the model does not account for the influence of ocean intrusion on basal pressure, nor does it include a subglacial hydrology system. Similarly, the basal friction coefficient is held constant throughout the simulations. We now explicitly acknowledge these limitations in the Methods section around line 307.

Reviewer Comment 1.23 — Line 265: remove "a" as there are two fields here; to obtain a three-dimensional velocity and pressure fields $-- >$ to obtain three-dimensional velocity and pressure fields

Reply: Done.

Reviewer Comment 1.24 — Line 269: Please note missing ref; simulating seawater intrusions with a linear ramp in basal melt builds on 21, 22, and 25.

Reply: Done. We have included missing references.

Reviewer Comment 1.25 — Line 281: Rather than stopping at 6 years, what happens after 8, 10, or 12 spin-up years if a constant SMB is maintained (Extended Data Fig 1B and Supp Fig 3B)? The downward drift in ice discharge between years 5 and 6 mirrors the upward drift between years 4 and 5. If the drift continues as a mirrored image, subsequent spin-up years might fall outside the target standard deviation range around the data. If that is the case, consider what impact starting with a drifting IC has on your results. On first take, it might mean having to kick the system a bit harder to overcome the diminishing ice discharge and reproduce the hindcasts. The larger grounding-zone and/or melt-rate forcing required to overcome the spin-up drift would then also lead to increased sea-level rise projections. Nature presents us with glaciers that are never truly in steady state with their ice-ocean-atmosphere-solid Earth-substrate system, so having a drifting starting point is quite reasonable. However, the chosen spin-up duration and then starting the experiments with the existing drift are modeling choices as opposed to fits with temporal trends in the data. A discussion regarding the impact of these choices on the results is therefore warranted.

Reply: Thank you for pointing out this issue. We agree that extending the spin-up period would likely modify the initial state and, consequently, the subsequent transient simulations. As the reviewer suggests, a longer spin-up could lead to increased grounding-zone melt and grounding-line retreat as the model continues to adjust and reduce the initial drift. The choice of a 6-year spin-up was made to ensure that (i) the modeled grounding line remains consistent with its observed location in 1996, and (ii) the modeled mass loss at the grounding line is in agreement with observations from the mid-1990s. In other words, we intentionally selected a spin-up duration that minimizes drift away from the observed glacier configuration at the start of the transient simulations. We acknowledge, however, that this is a modeling choice, and we now explicitly discuss the associated uncertainty in the manuscript around line 354.

Reviewer Comment 1.26 — Lines 296-297: I went back and forth with this one. As worded, the referenced studies did indeed indicate what is stated here. However, the preconditioning processes that can ultimately lead to calving as well as the very existence of an ice shelf (even if freely spreading) matter to Thwaites' past and future dynamics. If this sector continues to flow along as it has during the satellite era, "unlikely to be the dominant driver [...] under current conditions" might suffice here. However, there is healthy, ongoing scientific debate on this point and on Thwaites' dynamic trajectory. The challenge is deciding whether it is best to support your modeling choices with the dependent clause "although recent studies..." or simply to remind the reader that "It is important to note that the last metric to assess the full state of the glacier is the location of the ice front, as calving could lead to buttress removal and glacier acceleration." Whichever you decide, I would recommend rephrasing "last metric" to "last readily-observable metric" as these are all metrics that can be directly compared against the existing satellite record, but certainly not the only metrics that control the full state of the glacier.

Reply: Done. We have removed the sentence beginning with "Although" to avoid downplaying the importance of studies that investigate calving as a key process controlling the dynamics of Thwaites Glacier. We have also replaced the previous wording with "last readily observable metric" when referring to the calving front.

Reviewer Comment 1.27 — Line 328: Beyond stating that it serves as a normalization constant, it might be helpful to also briefly explain how the marginal likelihood is determined. As these are discrete parameter values for melt rate and grounding zone length that are being tested, wouldn't it simply be the sum of all tested combinations comprising the product in the numerator of (3)? If so, shouldn't Extended Data Fig. 3 look like discrete bar charts (probability mass functions) as opposed to continuous posterior distribution functions? Or perhaps, because of the linear interpolation and large sampling, you have generated enough discrete values to approximate them as continuous functions? Either way, please include a clarifying statement.

Reply: That is correct. Strictly speaking, the figure could indeed be presented as a discrete bar chart, since the prior ensemble is generated from a finite set of parameter combinations, ($\boldsymbol{\theta}_i$). However, for visualization purposes, we smoothed the discrete distribution to obtain a continuous curve. We now clarify this point in the figure's caption and around line 426. Furthermore, the reviewer is correct that the marginal likelihood is computed as the sum over all tested parameter combinations appearing in the numerator (line 409). We have revised the text to make this explicit.

Reviewer Comment 1.28 — References: Note many titles are missing capitalization of place names. e.g., thwaites west antarctica, etc.

Reply: We have fixed the references that were missing capitalizations.

Reviewer Comment 1.29 — Extended Data Fig. 3 caption: For improved readability, consider including commas, a semicolon, 'In', and 'in' 'employing the parameter, θ_i, to reproduce the data, y. In (A), θ_i is melt rate; in (B)θ_i is grounding zone length.'

Reply: Done.

Reviewer Comment 1.30 — Extended Data Fig. 5: For quick visual comparison and to help with the interpretation of the relative impact of the two parameters, it would be helpful if plots A and C had the same ordinate axis limits, and again for plots B and D.

Reply: Done.

Reviewer Comment 1.31 — Supplementary Figure 2 C: missing units on the basal friction coefficient, Cs

Reply: We now include units for friction coefficient.

Reviewer Comment 1.32 — Supplementary Figure 2 caption: to be consistent with Eqn 1: friction coefficient C on grounded ice friction coefficient Cs on grounded ice

Reply: Done.

Reviewer Comment 1.33 — Congratulations on this targeted and important contribution in advancing our understanding of the impact of grounding zones on ice dynamics, as supported by data, physical understanding, and theoretical reasoning.

Reply: Thank you again! We sincerely appreciate the reviewer's thoughtful comments and constructive suggestions. In our opinion, the review process has substantially improved the readability, clarity, and overall quality of this manuscript.

A.2 Response to reviewer 2

Reviewer Comment 2.1 — Summary

Poinelli et al. use an ice-sheet model to investigate the impact of basal melting, due to seawater intrusion, in the grounding-zone of Thwaites Glacier. They calibrate their model over a historical period and then use this to produce estimates of future sea-level contributions. Their method is to first initialize the model to an approximate 1990s state and then run an ensemble of hindcast simulations, while varying two parameters which control the magnitude and extent of basal melting. Using a Bayesian framework, they calibrate the grounding-zone melt parameters by comparing model performance with historical observations of ice thickness, ice speed and grounding-line position. To match the 1996 to 2023 observations they find that the model needs melt rates above 100m/yr and a grounding-zone of at least 1km, whereas simulations with no grounding-zone melting fail to reproduce observations. The authors therefore conclude that some degree of grounding-zone melting, due to seawater incursion, is needed in future modelling studies, and including this could push projections to the higher end of those given in the ISMIP6 ensemble.

The manuscript is very well written, with high quality figures, it flows well and is easy to read. The methodology is almost sufficient but would benefit from further details about model initialization (see below), as would the discussion. The results are clear from the figures and whilst the basal melt-rate parameterization is a simple depth-dependent approach, it shows how melting in the grounding-zone (via seawater intrusion or other means) could impact the future evolution of Thwaites Glacier. Whether the level of this melting is realistic (see below), it demonstrates that it plays a non-negligible role that certainly needs considering in other modelling studies and for other regions, particularly those glaciers that experience tidal uplift. I am encouraged to see the authors using combined observational constraints to calibrate their icesheet model over a historical period, before extending into future projections – in my mind this is definitely needed in more modelling studies.

I have some concerns related to the initialization of the ice-sheet model. My main concern is how the initialization impacts the transient evolution, particularly over the hindcast period as this is used for calibration, and whether the result of higher grounding-zone melting is compensating for errors in the initial setup.

Reply: We appreciate the effort of the reviewer in addressing our work. We are happy to hear that they find the study well-written and easy to read. Please find a detailed response to the reviewer's concerns regarding the initialization of the ice-sheet model and other issues raised by the reviewer below to this point.

Reviewer Comment 2.2 — My first question is how was the initial geometry generated? It is not clear whether the 2003-2004 ICESAT-corrected DEM from Bamber et al. (2009) represents the 1990s or mid-2000s. Could there be any temporal mismatches in data (surface elevation, grounding-line position, speed) that leads to a bias in the calibration?

The authors have made adjustments to the bed to achieve matching grounding-line positions, but could the mismatch be coming from temporal inconsistencies in input data? Whilst the authors show a limited comparison of modelled velocities against observations (Supp Fig 2D), it would be useful to see how the initial modelled geometry also compares.

Reply: Thank you for your comment, as it prompted us to perform a more in-depth review of the datasets used. Different that what stated in the original version of the manuscript, we do not use the 1 km Bamber (2009) DEM, which would indeed be more representative of the mid-2000s, as suggested by the reviewer. Instead, our initialization uses the 5 km DEM from Bamber (1997), which is derived from ERS-1 observations acquired during the 1994–1995 geodetic mission. We have revised the model description (around line 286)

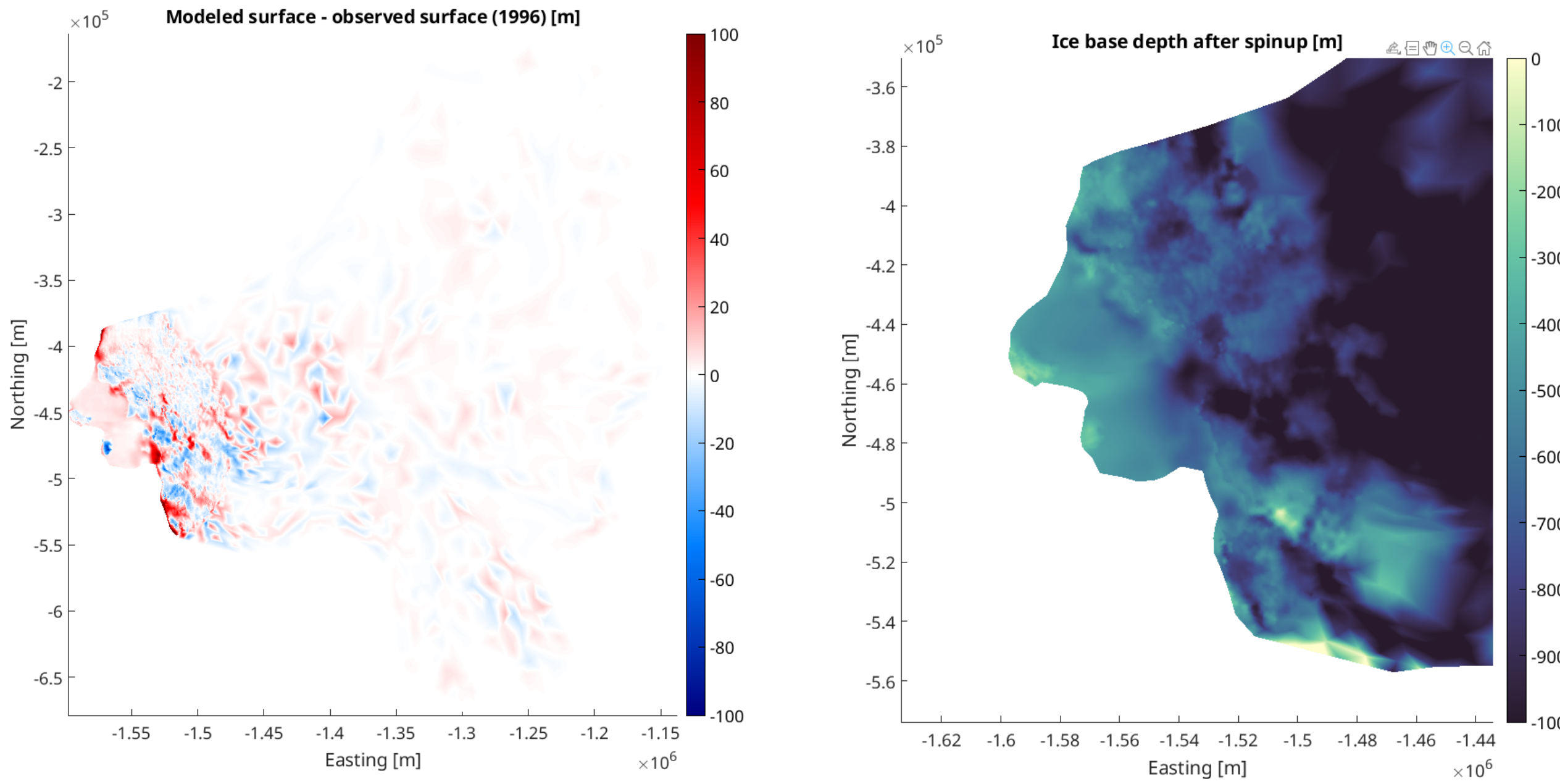

A **B**

Review Figure 2. A) Difference between the modeled ice surface after spin-up and the 1995 ice surface DEM of Bamber (1997). B) Ice base elevation after spin-up, illustrating that both pinning points are located at depths shallower than 300 m below sea level.

accordingly to clarify this. In our opinion, the use of this DEM, together with grounding-line positions from hydrostatic equilibrium (with appropriate seafloor correction) and ice velocities representative of the mid-1990s, provides the most consistent historical initialization currently achievable and minimizes temporal mismatches between the different observational datasets. Now, this configuration still lacks an appropriate representation of the ice front, please see our response to your comments 2.4, 2.5.

To further assess the quality of the initialization, we compare the model geometry after spin-up with the historical DEM. Although differences arise as the model adjusts to the prescribed bathymetry and forcing during the spin-up, these are generally on the order of 50 m and only locally reach approximately 100 m. We also calculated the total difference in ice volume between the post-spin-up model state and the volume derived from the Bamber DEM, which is 1.4%. Review Figure 2a illustrates these differences and demonstrates that the initialized geometry remains broadly consistent with the available historical observations. Biases in the calibration are certainly possible, given that the model is initialized using this specific combination of historical satellite datasets and the largest misfit is at the grounding line. We have added this figure in the supplement, instead of the modeled thickness to convey the idea and now mention the difference between initial modelled geometry and the observations, and mention the discrepancy in the Methods section (around line 354).

To this end, the modification of the bed topography was necessary to bring the modeled grounding line into closer agreement with the DInSAR-derived grounding-line position available for the mid-1990s. The fact that a purely hydrostatic assumption from the historical DEM and Bedmachine3.7 places the grounding line farther seaward than observed suggests that the glacier in this sector was possibly not in hydrostatic equilibrium at that time. As a result, modifying the bed geometry provides a physically plausible means of reconciling the modeled and observed grounding-line positions together with changes in ice thickness and ice speed, while remaining within the uncertainties of gravity inversion techniques.

For this reason, we believe that the remaining discrepancies are more likely attributable to the limitations of representing a partially grounded, non-hydrostatic glacier with a hydrostatic ice-sheet model than to the use of an temporal inconsistencies. As discussed above, the DEM, grounding-line position, and ice velocities

used for initialization are all representative of the 1990s. While these historical datasets are coarser, calculated as average over multiple years and more uncertain than modern observations, they are the best data available for reconstructing the glacier state during that period.

Concluding, the purpose of the spin-up is precisely to bring the model into a dynamically consistent state representative of the mid-1990s. Because correcting the bed geometry inevitably modifies the ice thickness near the grounding line, some differences between the spun-up geometry and the historical DEM are expected. However, the spin-up produces a glacier geometry and velocity field that together are consistent with the available observations. This is particularly important because the ice flux across the grounding line, which depends on both ice velocity and cross-sectional geometry, is the quantity that governs the modeled ice-loss rate. Therefore, while individual fields such as ice thickness or velocity may not perfectly match the observations everywhere, their combined effect yields a dynamically consistent reconstruction of the historical glacier state.

Reviewer Comment 2.3 — Furthermore, in Supp Fig. 3 the result of the spin-up shows a larger pinning point under the western tongue compared to 1996 observed – this could be providing additional buttressing which requires higher melt to match observed dynamics. Related to this, it is not clear from any of the figures what the extent of the pinning point at the front of the Eastern Ice Shelf is and how it compares against observations.

Reply: Regarding the reviewer's comment concerning pinning points. We agree that the treatment of pinning points is a limitation of the present study. We have not explicitly investigated their role, and in our model their surrounding floating ice is characterized by an ice base elevation shallower than approximately (-300) m (Review Figure 2b), while their extension is slightly larger than observations throughout the simulations. Consequently, basal melting is effectively suppressed over these regions in our simple depth-dependent parameterization. Because our analysis focuses on the differences between sensitivity experiments, this treatment acts as a constant offset that is present in all simulations and therefore does not influence the relative comparisons presented here. Nevertheless, we acknowledge that the representation of pinning points could affect the absolute evolution of the glacier and should be investigated in future work. We now explicitly discuss this limitation in the manuscript (around line 214) and in the methods (around lines 335, 371, 382) . Similarly, ice velocity over floating ice is subject to relatively large observational uncertainties, making direct comparisons challenging. In addition, the ice rheology is held fixed throughout the simulations, preventing us from assessing the influence of temporal variations in ice stiffness.

Reviewer Comment 2.4 — My final query about the initial geometry is about the calving front position, which differs quite a lot from the observed 1990s extended western front. The authors state that calving "is unlikely to be the primary driver of dynamic instability" but the extended front (and larger pinning points) could have played a role in its historical retreat (see Bett et al. 2026 [in review]).

Reply: Finally, regarding the ice-front position in model initialization. We also agree with the reviewer that some of our original statements may have understated the importance of calving and ice-front evolution in the historical retreat of the glacier. We have therefore removed or substantially softened these statements and replaced them with a more balanced discussion acknowledging that changes in the ice front likely played a role alongside ocean-driven melting. However, investigating the impact of calving is beyond the scope of the present study. The ice-front position is prescribed and held fixed in all simulations so that the experiments isolate the sensitivity of the glacier changes from calving. By keeping the ice front constant, its influence is effectively treated as a fixed offset that is common to all simulations, allowing us to isolate and highlight the relative differences arising from the imposed perturbations in melt and grounding-zone length. We have clarified this modeling choice in the text (around line 215 and 356) and now explicitly discuss it as a limitation of the study.

Reviewer Comment 2.5 — A second concern about the initialization is related to the model inversion. It has been previously shown that a snapshot inversion, using only surface velocities, can lead to reduced ice thinning compared to observations (Goldberg and Holland 2022). Furthermore, velocities in 1996-2001 were considerably slower (by 1km/yr) than the 2010s (Mouginot et al., 2014). Therefore, similar to my point above, is the calibration favouring higher melting in the grounding zone due to the lack of modelled ice thinning and slower velocities compared to observations? If a thickness change constraint was included in the inversion, how would this change the results?

Reply: Thank you for raising this important point. During the course of the project, we tested inversions for several different years. However, the resulting basal friction coefficients were generally noisy and did not exhibit a clear temporal trend toward either higher or lower values. The results are shown in Reviewer Figure 2 below. We agree that a transient inversion would provide a more physically consistent representation of the evolving ice thickness and basal conditions. However, such an approach would also continually update the model state toward the observed configuration, causing the simulation to naturally evolve toward the 2023 speed. This differs from the objective of the present study, which is to identify a set of model parameters through a sensitivity test that allows the model, starting from the mid-1990s configuration, to evolve toward the observed 2023 state without repeatedly assimilating available observations. As the reviewer notes, maintaining a fixed friction coefficient likely favors higher melt rates and a wider grounding zone to compensate for the absence of evolving basal friction associated with ice thinning and changing ice velocities, but this, in our opinion, is consistent with available observations, showing speedup across the area. We therefore view this as a modeling choice rather than a uniquely correct approach, with each strategy (fixed vs transient inversion) having different advantages depending on the scientific objective. We now explicitly acknowledge this limitation in the manuscript (around line 315 and in paragraph starting at line 338).

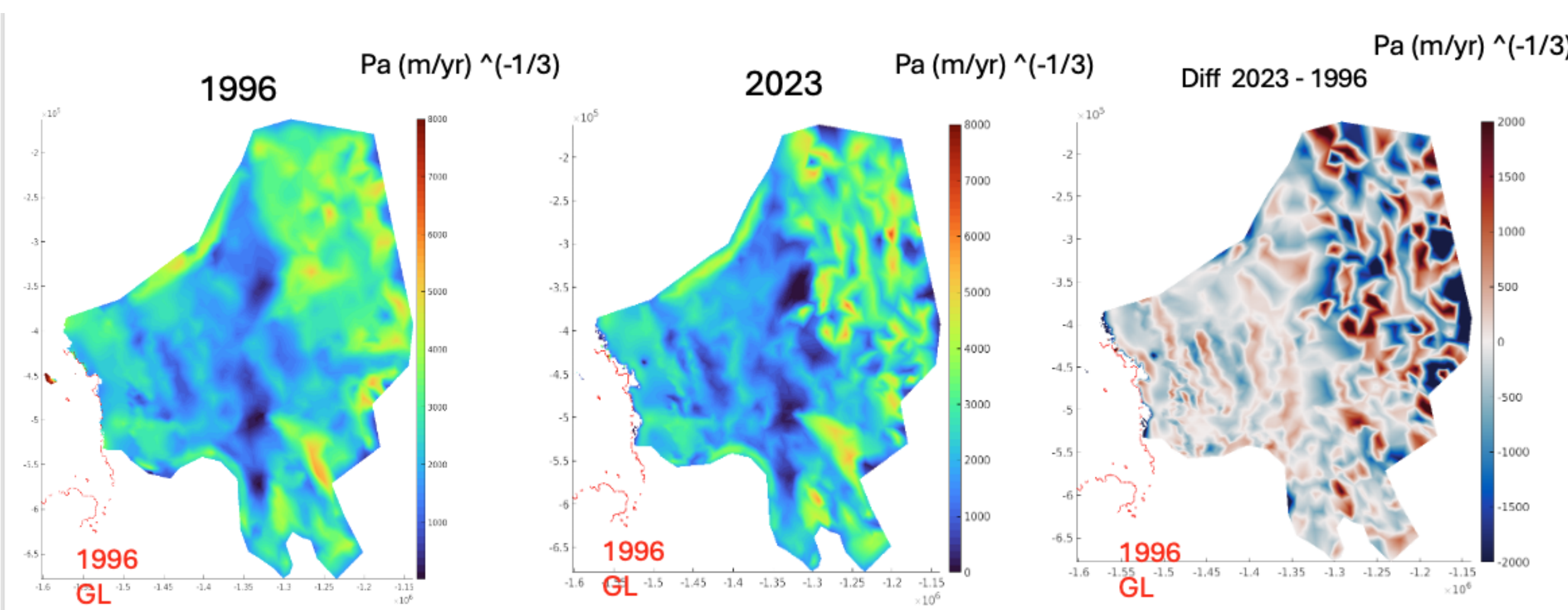

Review Figure 3. A) Friction inversion for 1996, B) friction inversion for 2023, C) difference between friction inversion in 2023 and 1996

Reviewer Comment 2.6 — If the authors add more details about the starting geometry and initialization procedure as well as putting the results into context regarding the initialization uncertainties, I would be happy for the manuscript to be published. Please see below more specific comments.

Reply: Thank you again for pointing out the need to clarify our choices in the model initialization procedure and put results into the context of initialization uncertainties. Finally, we have addressed the specific comments below.

Reviewer Comment 2.7 — Specific comments 12: "... only when grounding zones are represented as 2–5 km wide regions with melt rates of 110 m/yr" – this implies melt rates of 110m/yr across the grounding zone, but presumably this is just the maximum rate at X_2 (in Fig 1C) which then tapers down to 0. Can this be clarified and if so, adjust the wording here.

Reply: We have revised the abstract to reflect that melt applied is indeed at the entrance of the grounding zone i.e. X_2.

Reviewer Comment 2.8 — 12: "...producing 30±6 mm double previous projections" – is it fair to use this as the headline result when it is produced from a parameter set that is not the highest scoring? The top-scoring parameters lead to 20mm SLR.

Reply: Thank you for this insightful comment. As discussed later in the manuscript, the highest-scoring simulation produces a sea-level contribution that is consistent with the ISMIP ensemble mean, now included in Figure 4B. However, this parameter set provides the best fit to the observed historical evolution, during which observations indicate that the grounding-zone extent remained limited to approximately 2 km over the past two decades. More recent observations (ICEYE), however, suggest that seawater intrusion has increased to approximately 5–6 km, and even farther in some locations. Therefore, although these simulations receive relatively lower Bayesian scores when calibrated against the historical record, in the manuscript we speculate that they may be more representative of future glacier evolution, particularly given that melt rates and ocean thermal forcing have not changed substantially over the same period. Nevertheless, we agree with the reviewer's concern. We have toned down the corresponding statement in the Abstract and have included the highest-scoring simulation in Figure 4, as recommended. In our opinion, this provides a fairer comparison with previous studies and a more balanced evaluation of our results.

Reviewer Comment 2.9 — 17: "... where grounded ice becomes"

Reply: Done

Reviewer Comment 2.10 — 18: "...in the form of ice shelves"

Reply: Done

Reviewer Comment 2.11 — 18: Should this be modified Circumpolar Deep Water (mCDW)?

Reply: Yes, we have corrected the acronym here and throughout the manuscript.

Reviewer Comment 2.12 — 19: Is the Dutrieux reference for the "destabilizes the glacier" part? If not, put it after the ocean-induced melting phrase

Reply: Thanks for spotting it. We have moved the reference accordingly, as Dutrieux's reference does not discuss glacier destabilization.

Reviewer Comment 2.13 — 20: mCDW?

Reply: Done.

Reviewer Comment 2.14 — 20: "...eroded the floating ice base of Thwaites leading to progressive ungrounding from the seafloor"

Reply: Done.

Reviewer Comment 2.15 — 23: either "retrograde bed" regions/areas or "retrograde seafloor" regions/areas, not both.

Reply: Done. We now use retrograde seafloor throughout the manuscript.

Reviewer Comment 2.16 — 24: Please reword this sentence and be mindful of citation placement. This sentence sounds like the: Morlighem et al. (2020) study and Dos Santos et al. (2021) study show unstable behaviour but this is not the case.

Reply: Done, we have revised the sentence/references accordingly.

Reviewer Comment 2.17 — 25: What does "advances" mean here?

Reply: We meant: "Despite advances in numerical simulations".

Reviewer Comment 2.18 — 29: Is there a reference for "underestimated and overestimated" retreat mismatch?

Reply: This statement was primarily based on our observations from examining previous model reconstructions of Thwaites Glacier. To better support this point, we have now added the appropriate references to the manuscript.

Reviewer Comment 2.19 — 30: Do the references here all "point to deficiencies" as is mention?

Reply: Fricker et al. (Science) provides a clear overview of the key challenges that remain in ice-sheet modeling and highlights grounding-zone processes as one of the major unresolved priorities. We have now included this reference in this sentence.

Reviewer Comment 2.20 — 33: "...calibrate ice-sheet models" - Also see De Rydt (2026)

Reply: Done. We now include this reference.

Reviewer Comment 2.21 — 35: "...recent trends strongly influence near-future glacier evolution" – the Goldberg and Holland (2022) reference should be included here, and as mentioned in the summary above, this study (and Rosier et al. 2025) shows that thinning rates should be included in the model initialization.

Reply: Thank you for recommending the Goldberg and Holland publication, we now include this reference in our manuscript.

Reviewer Comment 2.22 — 41: No need to define CDW again.

Reply: That's right.

Reviewer Comment 2.23 — 42: "pioneering" is a judgement made by the authors, it would be preferable to use a neutral expression here.

Reply: We have removed 'pioneering' from the text.

Reviewer Comment 2.24 — 58: "Following calibration of the hindcast ensemble" – should this be calibration of the model?

Reply: Yes, we have corrected this sentence.

Reviewer Comment 2.25 — 60: Should be "grounding-zone melting" not 'physics'

Reply: Yes, done.

Reviewer Comment 2.26 — 63: Does "western and fastest-flowing sector" also mean 'central' because in line 21 it says "...in the glacier central, fastest-flowing sector" – for a general audience it should be consistent language

Reply: Thanks for pointing out the discrepancy, we now use 'western' to identify the faster flowing sector of Thwaites (TMT).

Reviewer Comment 2.27 — 64: Can the TMT be labelled on one of the figures, maybe Fig 1a or Supp Fig 1

Reply: Done. We now label TMT and TEIS on Figure 1a.

Reviewer Comment 2.28 — 72: It says here the model is initialized to "mid-1990s" but the methods suggest a DEM from mid-2000s

Reply: As mentioned earlier in our response to Reviewer Comment 2.2 , we revisited our code and found that we do not use the 1 km Bamber (2009) DEM, which, as the reviewer notes, would be more appropriate for the mid-2000s. Instead, we use the 5 km DEM from Bamber (1997), which is based on ERS-1 and observations acquired during the 1994–1995 geodetic phase. We have revised the manuscript accordingly to accurately describe the dataset used (line 287).

Reviewer Comment 2.29 — 83: Alterations are made to the bed elevation, but could uncertainties in initial ice geometry also contribute to the mismatch in grounding-line position?

Reply: Please see our response to your Comment 2.2.

Reviewer Comment 2.30 — 85: What impact does the bed lowering of 100m have on melt rates due to the depth-dependent parameterization?

Reply: There is no effect on the melt parameterization, as it depends only on the depth of the ice base. The 100 m lift is applied exclusively to floating ice, where BedMachine relies on gravity inversion in the absence of direct observations and where bed is below 1000 m. Consequently, this adjustment does not directly affect the computed melt rates because ice base is not modified, and the maximum melt rate remains the same. We have added a sentence to the Methods section to clarify this point (line 361).

Reviewer Comment 2.31 — 89: "as in traditional configurations" – is this shown in Fig 2 first column?

Reply: That is correct, we included a reference to this figure.

Reviewer Comment 2.32 — 90: "unrealistically high" – it isn't clear from the figures where these melt rates of 150m/yr are applied, but the results in Ext Fig 2 (and more in Chartrand et al 2024) shows regions of >150m/yr.

Reply: The melt rate is prescribed using a depth-dependent linear parameterization. Under this formulation, the maximum melt rate is reached at an ice-base depth of 900 m and remains constant for deeper ice. Even if there are some areas with melt rate above 150 m/yr, these are extremely localized (Chartrand 2024, Gadi 2025) so we believe that topping the rate at 150 m/yr is a good representation of basal ablation. We have

added clarifying sentences to the Methodology section to make this parameterization more explicit, referring to the maximum melt rate as $\dot{m}_{GL}$ (line 374 and 380).

Reviewer Comment 2.33 — 90: "align with observations" of what? If this refers to Fig 3, put a reference to it here.

Reply: We have added reference to Figure 3a.

Reviewer Comment 2.34 — 91: Replace "these results" with "these simulations..."

Reply: Done.

Reviewer Comment 2.35 — 95: Is the 150m/yr melt "relatively" high or 'unrealistically' high as was mentioned before?

Reply: For sake of consistency we now use 'relatively' rather than 'unrealistically', as there are areas with observed melt rate even higher that 150 m/yr.

Reviewer Comment 2.36 — 108: Why is there only a single value selected for the optimal melt rate, as Fig 4A shows a region between 90-120m/yr that corresponds to the selected grounding-zone length 2-5km.

Reply: That's right, we now mention the melt rate region between 90 and 120m/yr, rather than a defined value.

Reviewer Comment 2.37 — 118: "until reaching the next major topographic high" – this sounds like the retreat stops, but it continues in three of the model simulations

Reply: We have reworded this sentence to reflect this.

Reviewer Comment 2.38 — 133: It would be interesting to see the extended 2100 simulation with 1km grounding-zone length and 110m/yr melting on Fig 4B. In Ext Data Table 1, the final sea-level contribution for this simulation is 14.3mm which is similar to mean of ISMIP6 results.

Reply: Done. It is now in Figure 4b showing that 1 km grounding zone leads to sea level estimates that are similar to the ISMIP6 mean and we also include a statement in the text (line 180).

Reviewer Comment 2.39 — 140: "our results further establish calibration.." – it would be worth also noting (perhaps in the discussion) that whilst grounding-zone melt parameters are tuned here, there are other model parameters that could also be calibrated against these observations to improve projections.

Reply: Thank you for this helpful suggestion. We have revised the sentence to acknowledge that, in addition to the parameters considered in this study, there are many other model parameters and physical processes that can be calibrated and constrained using observations (line 158).

Reviewer Comment 2.40 — 146: "increase ocean temperature" – a shallowing of the thermocline is also predicted (Naughten et al., 2023), how would this impact the depth-dependent melt rates that are used in this study?

Reply: Thank you for this insightful comment! Based on results from Naughten et al. 2023, we can speculate that a shallower thermocline would likely lead to an even greater projected response, as a larger fraction of the ice shelf would be exposed to relatively warm modified Circumpolar Deep Water (mCDW). We have added a

sentence to the manuscript to acknowledge this point and discuss its potential implications (around line 170).

Reviewer Comment 2.41 — 151: It isn't clear from the figures what is meant here that the timing of unpinning from Mouginot Ridge "exerts a first-order control"

Reply: Thank you for this comment. What we intended to convey is that ridge unpinning plays a key role in controlling glacier retreat and the resulting sea-level contribution, as demonstrated, for example, by Houriez et al. (2024). We have revised the sentence accordingly to better reflect this point.

Reviewer Comment 2.42 — 153: Where is S6A?

Reply: Thanks for spotting it. This was a figure included in a previous version of the manuscript. We have removed the reference.

Reviewer Comment 2.43 — 155: The sea-level contribution of 8.3mm is for the 0m grounding-zone simulation only, not the "1-km grounding zone" which is also mentioned here and has a contribution of 14.3mm. Also include a reference to Ext Table 1 here.

Reply: Thanks for spotting this, we have revised the paragraph to distinguish between 0 km and 1 km grounding zone and added reference to Table 1 and Figure 4b.

Reviewer Comment 2.44 — 156: "inconsistent with the calibrated hindcast constraints" – some discussion is needed about how uncertainties in initialization would impact this sea-level contribution which is being compared against the ISMIP6 ensemble

Reply: As mentioned in our response to Reviewer Comment 2.2, the initial glacier geometry is derived from the historical Bamber DEM. Following the spin-up, the total ice volume differs from the Bamber DEM by 1.4%, and we now explicitly report this difference in the Methods section. It is difficult to directly compare our results in absolute sense with ISMIP6 or other modeling studies because each simulation starts from a different initial state (Seroussi 2024), including differences in initial ice flux at the grounding line. For this reason, we focus on changes relative to the 1996 state rather than differences in the absolute glacier geometry. We nevertheless acknowledge these sources of uncertainty and have expanded the discussion to place our results in the context of uncertainties associated with the initial conditions (notably around lines 197, 212, 315,).

Reviewer Comment 2.45 — 170: "grounding-zone extent exerts a stronger control on retreat and unpinning than moderate variations in thermal forcing." – This should be reworded considering the study uses a simple depth-dependent melt rate, which will not capture oceanic and geometric feedbacks as the grounding line retreats and the sub-shelf cavity changes shape (Holland et al., 2023; De Rydt , Naughten 2024)

Reply: We have revised the sentence to make the statement more balanced and have added the suggested references to support it.

Reviewer Comment 2.46 — 184: How does the melt parameterization prescribe "spatially uniform" rates if it is depth dependent (line 302)?

Reply: That's right, we have removed this sentence as it is confusing.

Reviewer Comment 2.47 — 187: If the melt rates are uniform (see above comment), then applying melt rates of 110m/yr uniformly would surely not be "consistent with satellite-derived estimates"?

Reply: That is correct and melt is not applied uniformly. We have revised this sentence.

Reviewer Comment 2.48 — 188: What does "effective" melt rates mean?

Reply: We have removed the word 'effective' as it is confusing.

Reviewer Comment 2.49 — 191: "limited by sparse observational constraints" – coupled ice-ocean models can also be constrained by ice dynamics, not just oceanic (e.g., Reed et al. 2026)

Reply: We have revised this sentence and add the reference to express this idea.

Reviewer Comment 2.50 — 195: "More importantly" than what?

Reply: We have reworded this.

Reviewer Comment 2.51 — 199: "substantially larger" is not true if taking the high-scoring 3km grounding-zone length.

Reply: We recognize that, although the highest-scoring simulation represents the best parameter set for reproducing the historical evolution of Thwaites Glacier, recent observations indicate that the grounding-zone extent has increased from approximately 3 km to 5–6 km over the past year. Consequently, for future projections, simulations with a wider grounding zone may be more representative, resulting in sea-level contributions that lie in the upper half of previous estimates. Nevertheless, we have reworded the corresponding sentence to better reflect this interpretation and to avoid overstating our conclusions.

Reviewer Comment 2.52 — Figures General: Figure panel labels are a mixture of lower case and upper case – please be consistent.

Reply: We have edited the figure labels to use lowercase throughout for consistency and to comply with the *Nature Communications* style guidelines.

Reviewer Comment 2.53 — Fig 1:

- (A) is showing the ice speed not "velocity".
- Add the hydrostatic-derived grounding line to (C)
- In (C) and elsewhere in the manuscript the grounding-zone length is an 'L' whereas in the caption it is correctly given as an italic. Please keep consistent.
- The caption says "dark brown" and "light brown" but there only appears to be one brown in the image.

Reply: Done, we have fixed these issues in Figure 1.

Reviewer Comment 2.54 — Fig 2: • Hyphen in figure x-label (Grounding-zone length)

Reply: Done.

Reviewer Comment 2.55 — Fig 3: • Y-label in (A) uses Gt/yr whereas in (C) the y-label uses m yr-1. Please be consistent.

Reply: Done, we have fixed the unit of Figure 3a.

Reviewer Comment 2.56 — Fig 4: • The title of this figure does not apply to (A).

Reply: That's right. We have changed the short title for Fig. 4.

Reviewer Comment 2.57 — Fig 5

- Why is it "Ridge Mouginot" in this figure, and "Mouginot Ridge" elsewhere?
- Caption: Should be "Modeled grounding-line position" rather than 'retreat'

Reply: We now use Mouginot Ridge consistently throughout the manuscript and have replaced the generic term retreat with the more precise grounding-line retreat where appropriate.

Reviewer Comment 2.58 — Ext Fig 1:

- Why is the 2021 grounding line shown here, rather than the end of the simulation?
- Caption: As above comment, why use "Ridge Mouginot" here?

Reply: You are correct. Thanks for spotting the typo. We intended to refer to the 2023 grounding line, not the 2021 grounding line. We have corrected the figure caption accordingly and have also updated the terminology to use Mouginot Ridge consistently.

Reviewer Comment 2.59 — Ext Fig 2:

- It is not clear what the multiple red lines are in (b). Are these the different melt rates that are included in the calibration?
- What year are the blue grounding lines referring to?

Reply: Yes, that is correct. The red lines in panel b represent the prescribed melt-rate parameterizations used in each calibration simulation along the dashed transect. Depending on the simulation, the maximum prescribed melt rate ranges from 10 to 150 m/yr. The grounding-zone extents shown correspond to the observed configurations in the 1990s and 2023. We have updated the figure caption to clarify these points.

Reviewer Comment 2.60 — Ext Fig 4: • Why is there an abbreviation MAP here?

Reply: This comes from a previous version of the manuscript. We have therefore removed it.

Reviewer Comment 2.61 — Ext Fig 5: • Insert a bracket before C) • Change the final (B) to (D)

Reply: Done.

Reviewer Comment 2.62 — Supp Fig 2: • (D) Why is there no data over much of the grounded domain? Methods

Reply: That is because we make misfits smaller than 30 m/yr transparent to improve the visibility of the larger differences. We now clarify this in the figure caption.

Reviewer Comment 2.63 — 234: Is there a mesh size of 500m throughout the grounding zone? With the SEM2 parameterization, how close is the melting to the grounded region?

Reply: Yes, the mesh resolution is 500 m throughout the grounding zone. Basal melting near the grounded ice is linearly tapered to zero over the length of the grounding zone, and the final grounded element is treated using the partial melting (SEM2) formulation of Seroussi et al. (2014). We have revised the Methods section to make this treatment clearer (line 277).

Reviewer Comment 2.64 — 236: "conditions representative of the 1990s" – are the input datasets temporally consistent (see the main comment in the summary)?

Reply: Please see our response to Comment 2.2.

Reviewer Comment 2.65 — 238: "model domain is based on a 1996-2001 velocity map" – Is this why the ice front does not match the 1996 observations? How would an extended ice front impact the results?

Reply: That is correct, the ice front was selected to use available velocity map. We have not tested the impact of an extended ice front but other studies, which we reference in the manuscript have done so, showing different impacts of ice front on dynamics. This goes beyond the scope of this simulations and we mention this in the methods section (around lines 197, 212, 315).

Reviewer Comment 2.66 — 244: As mentioned above in the summary, could the mismatch in grounding-line position be due to inconsistencies in data input rather than bed elevation errors?

Reply: This is certainly a possibility, given the uncertainties associated with satellite observations from the 1990s and with our model initialization. However, in our view, combining the available historical observations with a spin-up designed to bring the model into a state consistent with the observed historical ice-loss signal represents the best achievable approach for reconstructing past glacier evolution and sea level commitment over the century. Nevertheless, uncertainties remain, particularly regarding the historical ice-front position and the location of pinning points, as highlighted by the reviewer. Furthermore, bed elevation under grounded ice is obtained with gravity inversions which has errors of hundred of meters. We have expanded the discussion of these limitations and the associated uncertainties throughout the manuscript (notably around lines 197, 212, 315) to better acknowledge these sources of error and address the reviewer's concerns on model initialization and uncertainties.

Reviewer Comment 2.67 — 248: What is the effective timestamp of the DEM?

Reply: The effective timestamp of the DEM is 1994-1995 as indicated in Bamber 1997. We have corrected this in the text where we had erroneously mentioned the 2001 paper.

Reviewer Comment 2.68 — 254: Why is the velocity difference in Supp Fig 2D only shown for a small region? Also, is this actually a difference in speed (not velocity)?

Reply: That is because we make misfits smaller than 30 m/yr transparent to improve the visibility of the larger differences. We now clarify this in the figure caption, also referring to speed rather than velocity.

Reviewer Comment 2.69 — 270: Is this "maximum value at the grounding line" the parameter that is being calibrated in this study? If so, it would be helpful to mention here and in Fig 1C.

Reply: Yes, this is the calibration procedure used in our study. We now state this explicitly in the Hindcast Ensemble section of the Methods (line 384) and have also added $\dot{m}_{GL}$ to Figure 1c for clarity.

Reviewer Comment 2.70 — 284: "absence of a basal melt rate" – Is there a subsequent shock when the basal melt rate is applied?

Reply: Thank you for spotting this inconsistency. During the spin-up, we actually apply a constant basal melt rate of 10 m/yr. The reference to the absence of basal melt was a remnant from an earlier version of the manuscript. We have corrected this statement accordingly. Introducing a different prescribed melt rate at the start of the sensitivity simulations does produce an initial model adjustment. However, we deliberately use the same spin-up configuration for all simulations so that each experiment starts from a common baseline, allowing for a consistent comparison of the sensitivity experiments..

Reviewer Comment 2.71 — 285: How much does the geometry and velocities deviate from the initial 1990s state during the spinup?

Reply: The purpose of the spin-up is to bring the model to a state that is representative of the glacier in the 1990s. Specifically, we seek to reproduce both the observed geometry and ice velocity, such that the resulting ice flux across the grounding line (i.e., velocity multiplied by the grounding-line cross-sectional area) is consistent with observations. We therefore use grounding-line ice flux as the primary metric to evaluate the spin-up and determine when the model has reached an appropriate initial state for the sensitivity experiments. The initial modeled ice volume is 1.4% lower from observations.

Reviewer Comment 2.72 — 294: It is not clear what this sentence about the ice front is saying. It cannot be used as a constraint because the ice front is fixed, isn't it?

Reply: Yes, in our simulations the ice front is fixed. What we intended to convey is that reproducing the observed ice-front retreat would provide an additional metric for evaluating model performance against observations. However, because the calving front is prescribed and does not evolve in our simulations, we do not investigate this aspect here. We have revised the sentence accordingly to make this distinction clear.

Reviewer Comment 2.73 — 299: "melting rate under floating ice" – isn't this parameter describing the maximum melt rate at and below 800m (line 303)? So, it is depth-dependent, not "under floating ice". Is this parameter $\dot{m}$ also the same as $\dot{m}_{GL}$ in Eq. (2)?

Reply: That is correct, we meant $\dot{m}_{GL}$ in Eq. (2). We have corrected the usage of $\dot{m}_{GL}$ throughout the manuscript when referring to the maximum melt rate as calibration parameter of our simulations.

Reviewer Comment 2.74 — 304: Is this maximum melt rate the parameter being calibrated in the study?

Reply: Yes, that is correct. The maximum melt rate reached at an ice-base depth of 900 m is the parameter that we calibrate in this study. To make this clearer, we have revised the corresponding paragraph and now consistently refer to this calibrated parameter as $\dot{m}_{GL}$.

Reviewer Comment 2.75 — 322: Ensure that "grounding-zone length" has a hyphen, it is not consistent in the manuscript.

Reply: Done. We have revised this throughout the manuscript.

Reviewer Comment 2.76 — 325: Should the posterior distribution parameter be $\hat{P}$ instead of P? Similarly, elsewhere: L343, 347, Ext Fig 3 caption

Reply: That's correct, thanks for spotting it. We have corrected this throughout the manuscript.

Reviewer Comment 2.77 — 340: What are the model and observational uncertainties used here?

Reply: In this equation, (σ) represents the uncertainty associated with the observed values, (y). Specifically, we use the observational uncertainties (standard deviations) provided with each observational dataset. We now clarify this immediately following the equation in the manuscript. As our model produces a deterministic solution for a given set of initial and boundary conditions, we do not explicitly propagate uncertainties through the model results. Instead, our sensitivity experiments are designed to assess and reduce the impact of uncertainties in the initial state by quantifying how variations in the initial conditions influence the simulated glacier evolution.

Reviewer Comment 2.78 — 345: What does "weighted simulation" mean here? It isn't clear how many simulations are extended to 2100.

Reply: Since we extend all simulations to 2100, we have removed 'weighted' to avoid confusion.

Reviewer Comment 2.79 — Supplement Make sure the Supplement title is the same as main manuscript.

Reply: Done.

Reviewer Comment 2.80 — Remarks on code availability: The title of the repository does not match the current manuscript. The file description on the repository says there is a 'step 16' to reproduce the sensitivity simulations in the paper, but I cannot find this. The repository also mentions step 11 in the 'readme.m' but this doesn't seem to exist. I could not load the mat file as intended by the author (as a 'model' format), error is: "Warning: Variable 'md' originally saved as a model cannot be instantiated as an object and will be read in as a uint32." I could not run the Matlab script, I got the error: Unrecognized function or variable 'organizer'.

Reply: We have updated the Zenodo repository to reflect the revised manuscript title. The MATLAB file can only be loaded once ISSM has been properly installed. ISSM can be installed by following the instructions provided on the official GitHub repository: `https://github.com/ISSMteam/ISSM`. We now include this information in the README file. Similarly, running the routines requires a local ISSM installation, and we now direct readers to the installation instructions in the documentation. Step 16 of the workflow can then be executed using the command `perform(org,'Projection.GZ-Melt-Sensitivity')`, after introducing this global variable `step=16`.

Manuscript: NCOMMS-26-033269-T

Title: Sea-level contribution from Thwaites Glacier doubles in an observation-constrained ice-sheet model

Authors: Mattia Poinelli, Eric Rignot, Hélène Seroussi, Josh Cuzzone, Tyler Pelle, Eric Larour

A Response to the anonymous reviewers

We would like to thank again the two anonymous reviewers of *Nature Communications* for their constructive comments and suggestions. Please find our responses below to this point. For the reviewers' and editor's convenience, we have highlighted our responses to the comments in blue. Please note that when we mention specific lines in the response to reviewers, we are referring to lines number in the new version of the manuscript.

Contents

A Response to the anonymous reviewers **1**

A.1 Response to reviewer 1 2

A.2 Response to reviewer 2 5

A.1 Response to reviewer 1

Reviewer Comment 1.1 — General Comments

The authors did a fantastic job addressing reviewer concerns from the first round. Congratulations on your important, carefully-planned, and well-presented research contribution. Thank you for your thorough revision efforts. As this is my second review, I have a few minor points to consider.

Reply: Thank you once more for your time assessing our work. We are happy to hear that you have enjoyed our work. Please find our responses to your comments below.

Reviewer Comment 1.2 — - Specific Comments

Fig. 1 caption: I realize this is in direct conflict with Ref. 2's first round of comments, but I see blue, dark brown, and light brown on my image per the original caption. I do not see "grey" elements. Please triple check this.

Reply: That's odd. In our PDF version, the elements are blue, grey and brown, but there may be an issue with rendering of this image, as the parameterization panel was uploaded after the figure was generated. We took note of this issue and will work with the editorial team at publication/proofreading time to make sure the colors are appropriately labeled.

Reviewer Comment 1.3 — Line 106: calculated as spatial average −− > calculated as a spatial average

Reply: Done.

Reviewer Comment 1.4 — Line 156 and Supplementary Fig. 8 d): If I'm interpreting Supp Fig 8 correctly, both panels (a) and (c) include the full suite of experiments (all grounding zone lengths, L, in (a) and all melt rates in (c)). What (b) and (c) are illustrating is that i) for melting rates, above about 40 m/yr, additional melting still leads to more SLE, but at a similar % increase for each 10 m/yr jump, whereas ii) for L, the biggest SLE % change occurs by simply introducing a grounding zone; thereafter, while SLE still rises with increasing grounding zone length, the rate of SLE change diminishes with each added km. Nevertheless, the mean increase in % SLE change is higher for L changes than it is for basal melting rate changes. If this is all correct, then shouldn't the text in the Fig. 8 panel d state "Mean increase", not "Mean decrease", despite the downward trend in % SLE change with increasing L?

Reply: Thanks for spotting this. Yes, that is the correct interpretation of the figure. What we calculate in Supplementary Fig. 8 is exactly the average increase in % SLE change with consecutive increases in grounding-zone length and basal melt rate. We have corrected the figure accordingly, which now says 'Mean increase' and added a sentence in the manuscript around line 157 to state that the biggest SLE % change occurring by simply introducing a grounding zone, when comparing it to simulations with no grounding zones.

Reviewer Comment 1.5 — Line 216: extra period

Reply: Done, thanks for spotting this typo.

Reviewer Comment 1.6 — Line 229: M.P. build model domain and run the sensitivity study −− > M.P. built the model domain and ran the sensitivity study

Reply: Done.

Reviewer Comment 1.7 — Line 269: to remove any ambiguity between ice bed and seafloor consider either: The elevation of the bed is increased −− > the elevation of the seabed is increased -or- the bathymetry is increased

Reply: Done, we now use seafloor for consistency throughout the manuscript.

Reviewer Comment 1.8 — Line 270: with bed elevation of approximately 1000 m. −− > with seafloor depth of approximately -1000 m.

Reply: Done.

Reviewer Comment 1.9 — Line 274: Best to remain consistent throughout the manuscript with ice stiffness, ice hardness, and rheology, all being used interchangeably for the same parameter, B, which is often referred to as the ice hardness parameter. I would recommend against calling B the ice rheology, as this is a temperature dependent parameter within the flow law that governs the rheology of ice that is implemented. While indeed part of the rheological equation, the power, n, is more influential in specifying the simulated "power-law" ice rheology, just as m is more influential than the basal friction coefficient in specifying the simulated basal rheology. We derive ice stiffness for floating ice −− > We derive the ice hardness parameter for floating ice -or- We derive the ice hardness for floating ice

Reply: Done, thank you for the suggestion. We now realize the ambiguity of using different terms for the parameter B, which can be confusing. We now use ice hardness when referring to the parameter B, throughout the manuscript.

Reviewer Comment 1.10 — Lines 280-281, 285-287: Though you use a regularized Coulomb friction law that depends on N, you are not including a water system (stated on Line 194; helpful to reiterate here). You then elect to calculate the effective pressure as equal to the evolving ice overburden pressure everywhere for THW (i.e., you are thereby assuming zero water pressure, which equates to either a frozen bed, highly efficient infiltration into underlying aquifers, or an extremely efficient, channelized basal water system to help dewater most of the bed). This simplifying assumption, N−− >Pi, does not match existing data for most of THW, other than near channelized systems along the margins, around bedrock highs and fluted bedforms, and/or perhaps proximal to the grounding zone to help efficiently feed subglacial water to the ocean (until the point where ocean influences reach upglacier into the grounding zone). Furthermore, isn't N = Pi directly contrary to the Iken assumption of a perfectly lubricated bed, which is the essence of that particular component of the Coulomb friction law? Based on your careful post-processing analysis to address another review question about where the Iken limit is activated in the model domain versus sliding following an m=3 power-law, you now report that Coulomb sliding only kicks in across the fastest-flowing regions, just upstream of the grounding line. Therefore, while Pw = 0 Pa does not match data nor the conditions for Coulomb sliding and ocean incursion, given that Coulomb sliding was only activated in regions we might anticipate are ripe for reduced dynamic drag, the choice of this sliding law might have fortuitously helped partially compensate for the static and high basal friction coefficient field across this region. Inasmuch, the targeted sensitivity results and discussions remain reasonable under your stated modeling assumptions. However, for the benefit of the wider readership, please consider including an additional comment regarding the known oversimplification of assigning N = Pi when simulating THW. In that discussion, you might point out that, fortunately, across most of the natural domain where Pw −− > Pi and therefore effective pressures are actually low, a power-law bed rheology dominates your simulated sliding and therefore the assignment of overburden pressures everywhere is essentially ignored.

Reply: Thanks for your comment and suggestion regarding the proper usage of friction laws. It's correct that we do not include any subglacial hydrological network, and we have added a further sentence around line

280 to reiterate on this important limitation of our model set-up. To further address the reviewer's comment, we have included a few sentences describing the usage of $N = P_i$ as a limitation of the model, also pointing out the across most of the natural domain where effective pressures are typically low, the $m = 3$ power-law dominates the sliding of the glacier, as the reviewer correctly suggests. You will find these changes around line 294. Thank you again for your comments about sliding laws, which led us to a more accurate investigation of the model behavior under the applied assumptions and simplifications. In our opinion, the results and the analysis are now much more robust and considerate of model limitations.

Reviewer Comment 1.11 — Line 291: As above To retrieve the rheology of grounded ice $-->$ To retrieve the ice hardness of grounded ice

Reply: Done.

Reviewer Comment 1.12 — Line 296: As above To inform the ice rheology $-->$ to inform the ice hardness

Reply: Done.

Reviewer Comment 1.13 — Line 325: As above To this end, the bed correction $-->$ To this end, the seabed correction -or- To this end, the bathymetric correction

Reply: Done.

Reviewer Comment 1.14 — Lines 334-336 and again on Lines 344-345: Please check your output to make sure the drag on the pinning points remained unchanged throughout the simulations. With enhanced flux across the grounding line and the highest melt rates concentrated near the grounding line, there is significant potential to change the drag across the far-field pinning points. Changes in flux lead to thickness propagations across the ice shelf and concentrating melt at depth effectively planes off ice layers that would otherwise be advected across all remaining down-glacier sites. (i.e., Basal melt need not be proximal to the pinning point to impact the buttressing it offers.) Even if the simulated drag happens to remain unchanged on the pinning points, the wording here needs to be changed because of these potential dynamic feedbacks.

Reply: To address the reviewer's comment we have calculated the basal traction beneath the pinning points and find that, over the course of the simulations, the maximum increase in basal drag is approximately 5% relative to the original drag distribution, even under the very high melt rate of 150 m yr^{-1}. In the other simulations, basal traction remains within 1% of its initial value at the beginning of the simulations. To factor this, we have reworded the sentences regarding pinning points, in particular removing the sentence around line 355, where our wording suggested that melt nearby pinning point is the only factor to take into account. Indeed, as the reviewer suggests, basal melt does not need to be proximal to a pinning point to affect buttressing.

Reviewer Comment 1.15 — Supplementary Figure 4 caption: As above Implications of the 100 m bed correction $-->$ Implications of the 100 m seabed correction with original bed topography $-->$ with original seabed topography -or- with original bathymetry

Reply: Done.

Reviewer Comment 1.16 — Again, well done and thank you for your contribution.

Reply: Thank you again! We sincerely appreciate the reviewer's thoughtful comments and constructive suggestions. In our opinion, the review process has substantially improved the readability, clarity, and overall quality of this manuscript.

A.2 Response to reviewer 2

Reviewer Comment 2.1 — Having read the revised manuscript from Poinelli et al., I am pleased to see that all of my suggestions have been taken into account. In particular, the inclusion of statements regarding uncertainty in initial geometry and limitations with the model setup. I am satisfied that all of my previous comments have been addressed and will be happy to see the manuscript published.

Reply: We are happy to hear that the reviewer is satisfied with our edits. Thank you for reviewing our work and for your suggestions, which, in our opinion made the message of the paper much stronger.

Sea-level contribution from Thwaites Glacier doubles in an observation-constrained ice-sheet model

Summary

Poinelli et al. use an ice-sheet model to investigate the impact of basal melting, due to seawater intrusion, in the grounding-zone of Thwaites Glacier. They calibrate their model over a historical period and then use this to produce estimates of future sea-level contributions. Their method is to first initialize the model to an approximate 1990s state and then run an ensemble of hindcast simulations, while varying two parameters which control the magnitude and extent of basal melting. Using a Bayesian framework, they calibrate the grounding-zone melt parameters by comparing model performance with historical observations of ice thickness, ice speed and grounding-line position. To match the 1996 to 2023 observations they find that the model needs melt rates above 100m/yr and a grounding-zone of at least 1km, whereas simulations with no grounding-zone melting fail to reproduce observations. The authors therefore conclude that some degree of grounding-zone melting, due to seawater incursion, is needed in future modelling studies, and including this could push projections to the higher end of those given in the ISMIP6 ensemble.

The manuscript is very well written, with high quality figures, it flows well and is easy to read. The methodology is almost sufficient but would benefit from further details about model initialization (see below), as would the discussion. The results are clear from the figures and whilst the basal melt-rate parameterization is a simple depth-dependent approach, it shows how melting in the grounding-zone (via seawater intrusion or other means) could impact the future evolution of Thwaites Glacier. Whether the level of this melting is realistic (see below), it demonstrates that it plays a non-negligible role that certainly needs considering in other modelling studies and for other regions, particularly those glaciers that experience tidal uplift. I am encouraged to see the authors using combined observational constraints to calibrate their ice-sheet model over a historical period, before extending into future projections – in my mind this is definitely needed in more modelling studies.

I have some concerns related to the initialization of the ice-sheet model. My main concern is how the initialization impacts the transient evolution, particularly over the hindcast period as this is used for calibration, and whether the result of higher grounding-zone melting is compensating for errors in the initial setup.

My first question is how was the initial geometry generated? It is not clear whether the 2003-2004 ICESAT-corrected DEM from Bamber et al. (2009) represents the 1990s or mid-2000s. Could there be any temporal mismatches in data (surface elevation, grounding-line position, speed) that leads to a bias in the calibration? The authors have made adjustments to the bed to achieve matching grounding-line positions, but could the mismatch be coming from temporal inconsistencies in input data? Whilst the authors show a limited comparison of modelled velocities against observations (Supp Fig 2D), it would be useful to see how the initial modelled geometry also compares. Furthermore, in Supp Fig. 3 the result of the spin-up shows a larger pinning point under the western tongue compared to 1996 observed – this could be providing additional buttressing which requires higher melt to match observed dynamics. Related to this, it is not clear from any of the figures what the extent of the pinning point at the front of the Eastern Ice Shelf is and how it compares against observations. My final query about the initial geometry is about the calving front position, which differs quite a lot from the observed 1990s extended western front. The authors state that calving “is unlikely to be the primary driver of dynamic instability” but the extended front (and larger pinning points) could have played a role in its historical retreat (see Bett et al. 2026 [in review]).

A second concern about the initialization is related to the model inversion. It has been previously shown that a snapshot inversion, using only surface velocities, can lead to reduced ice thinning compared to observations (Goldberg and Holland 2022). Furthermore, velocities in 1996-2001 were considerably slower (by 1km/yr) than the 2010s (Mouginot et al., 2014). Therefore, similar to my point above, is the calibration favouring higher melting in the grounding zone due to the lack of modelled ice thinning and slower velocities compared to observations? If a thickness change constraint was included in the inversion, how would this change the results?

If the authors add more details about the starting geometry and initialization procedure as well as putting the results into context regarding the initialization uncertainties, I would be happy for the manuscript to be published. Please see below more specific comments.

Specific comments

- 12: "…only when grounding zones are represented as 2–5 km wide regions with melt rates of 110 m yr^{-1}" – this implies melt rates of 110m/yr across the grounding zone, but presumably this is just the maximum rate at x_2 (in Fig 1C) which then tapers down to 0. Can this be clarified and if so, adjust the wording here.
- 12: "…producing 30±6 mm double previous projections" – is it fair to use this as the headline result when it is produced from a parameter set that is not the highest scoring? The top-scoring parameters lead to 20mm SLR.
- 17: "…where grounded ice becomes"
- 18: "…in the form of ice shelves"
- 18: Should this be modified Circumpolar Deep Water (mCDW)?
- 19: Is the Dutrieux reference for the "destabilizes the glacier" part? If not, put it after the ocean-induced melting phrase
- 20: mCDW?
- 20: "…eroded the floating ice base of Thwaites leading to progressive ungrounding from the seafloor"
- 23: either "retrograde bed" regions/areas or "retrograde seafloor" regions/areas, not both.
- 24: Please reword this sentence and be mindful of citation placement. This sentence sounds like the Morlighem et al. (2020) study and Dos Santos et al. (2021) study show unstable behaviour but this is not the case.
- 25: What does "advances" mean here?
- 29: Is there a reference for "underestimated and overestimated" retreat mismatch?
- 30: Do the references here all "point to deficiencies" as is mention?
- 33: "…calibrate ice-sheet models" - Also see De Rydt (2026)
- 35: "…recent trends strongly influence near-future glacier evolution" – the Goldberg and Holland (2022) reference should be included here, and as mentioned in the summary above, this study (and Rosier et al. 2025) shows that thinning rates should be included in the model initialization.
- 41: No need to define CDW again.
- 42: "pioneering" is a judgement made by the authors, it would be preferable to use a neutral expression here.
- 58: "Following calibration of the hindcast ensemble" – should this be calibration of the model?
- 60: Should be "grounding-zone melting" not 'physics'
- 63: Does "western and fastest-flowing sector" also mean 'central' because in line 21 it says "…in the glacier central, fastest-flowing sector" – for a general audience it should be consistent language
- 64: Can the TMT be labelled on one of the figures, maybe Fig 1a or Supp Fig 1
- 72: It says here the model is initialized to "mid-1990s" but the methods suggest a DEM from mid-2000s

- 83: Alterations are made to the bed elevation, but could uncertainties in initial ice geometry also contribute to the mismatch in grounding-line position?
- 85: What impact does the bed lowering of 100m have on melt rates due to the depth-dependent parameterization?
- 89: “as in traditional configurations” – is this shown in Fig 2 first column?
- 90: “unrealistically high” – it isn’t clear from the figures where these melt rates of 150m/yr are applied, but the results in Ext Fig 2 (and more in Chartrand et al 2024) shows regions of >150m/yr.
- 90: “align with observations” of what? If this refers to Fig 3, put a reference to it here.
- 91: Replace “these results” with “these simulations...”
- 95: Is the 150m/yr melt “relatively” high or ‘unrealistically’ high as was mentioned before?
- 108: Why is there only a single value selected for the optimal melt rate, as Fig 4A shows a region between 90-120m/yr that corresponds to the selected grounding-zone length 2-5km.
- 118: “until reaching the next major topographic high” – this sounds like the retreat stops, but it continues in three of the model simulations
- 133: It would be interesting to see the extended 2100 simulation with 1km grounding-zone length and 110m/yr melting on Fig 4B. In Ext Data Table 1, the final sea-level contribution for this simulation is 14.3mm which is similar to mean of ISMIP6 results.
- 140: “our results further establish calibration..” – it would be worth also noting (perhaps in the discussion) that whilst grounding-zone melt parameters are tuned here, there are other model parameters that could also be calibrated against these observations to improve projections.
- 146: “increase ocean temperature” – a shallowing of the thermocline is also predicted (Naughten et al., 2023), how would this impact the depth-dependent melt rates that are used in this study?
- 151: It isn’t clear from the figures what is meant here that the timing of unpinning from Mouginot Ridge “exerts a first-order control”
- 153: Where is S6A?
- 155: The sea-level contribution of 8.3mm is for the 0m grounding-zone simulation only, not the “1-km grounding zone” which is also mentioned here and has a contribution of 14.3mm. Also include a reference to Ext Table 1 here.
- 156: “inconsistent with the calibrated hindcast constraints” – some discussion is needed about how uncertainties in initialization would impact this sea-level contribution which is being compared against the ISMIP6 ensemble
- 170: “grounding-zone extent exerts a stronger control on retreat and unpinning than moderate variations in thermal forcing.” – This should be reworded considering the study uses a simple depth-dependent melt rate, which will not capture oceanic and geometric feedbacks as the grounding line retreats and the sub-shelf cavity changes shape (Holland et al., 2023; De Rydt & Naughten 2024)
- 184: How does the melt parameterization prescribe “spatially uniform” rates if it is depth dependent (line 302)?
- 187: If the melt rates are uniform (see above comment), then applying melt rates of 110m/yr uniformly would surely not be “consistent with satellite-derived estimates”?
- 188: What does “effective” melt rates mean?

- 191: “limited by sparse observational constraints” – coupled ice-ocean models can also be constrained by ice dynamics, not just oceanic (e.g., Reed et al. 2026)

- 195: “More importantly” than what?

- 199: “substantially larger” is not true if taking the high-scoring 3km grounding-zone length.

Figures

General: Figure panel labels are a mixture of lower case and upper case – please be consistent.

Fig 1:
- (A) is showing the ice speed not “velocity”.
- Add the hydrostatic-derived grounding line to (C)
- In (C) and elsewhere in the manuscript the grounding-zone length is an ‘L’ whereas in the caption it is correctly given as an italic L. Please keep consistent.
- The caption says “dark brown” and “light brown” but there only appears to be one brown in the image.

Fig 2:
- Hyphen in figure x-label (Grounding-zone length)

Fig 3:
- Y-label in (A) uses Gt/yr whereas in (C) the y-label uses m yr^{-1}. Please be consistent.

Fig 4:
- The title of this figure does not apply to (A).

Fig 5
- Why is it “Ridge Mouginot” in this figure, and “Mouginot Ridge” elsewhere?
- Caption: Should be “Modeled grounding-line <u>position</u>” rather than ‘retreat’

Ext Fig 1:
- Why is the 2021 grounding line shown here, rather than the end of the simulation?
- Caption: As above comment, why use “Ridge Mouginot” here?

Ext Fig 2:
- It is not clear what the multiple red lines are in (b). Are these the different melt rates that are included in the calibration?
- What year are the blue grounding lines referring to?

Ext Fig 4:
- Why is there an abbreviation MAP here?

Ext Fig 5:
- Insert a bracket before C)
- Change the final (B) to (D)

Supp Fig 2:
- (D) Why is there no data over much of the grounded domain?

Methods

- 234: Is there a mesh size of 500m <u>throughout</u> the grounding zone? With the SEM2 parameterization, how close is the melting to the grounded region?

- 236: “conditions representative of the 1990s” – are the input datasets temporally consistent (see the main comment in the summary)?
- 238: “model domain is based on a 1996-2001 velocity map” – Is this why the ice front does not match the 1996 observations? How would an extended ice front impact the results?
- 244: As mentioned above in the summary, could the mismatch in grounding-line position be due to inconsistencies in data input rather than bed elevation errors?
- 248: What is the effective timestamp of the DEM?
- 254: Why is the velocity difference in Supp Fig 2D only shown for a small region? Also, is this actually a difference in speed (not velocity)?
- 270: Is this “maximum value at the grounding line” the parameter that is being calibrated in this study? If so, it would be helpful to mention here and in Fig 1C.
- 284: “absence of a basal melt rate” – Is there a subsequent shock when the basal melt rate is applied?
- 285: How much does the geometry and velocities deviate from the initial 1990s state during the spin-up?
- 294: It is not clear what this sentence about the ice front is saying. It cannot be used as a constraint because the ice front is fixed, isn’t it?
- 299: “melting rate under floating ice” – isn’t this parameter describing the maximum melt rate at and below 800m (line 303)? So, it is depth-dependent, not “under floating ice”. Is this parameter $\dot{m}$ also the same as $\dot{m}_{GL}$ in Eq. (2)?
- 304: Is this maximum melt rate the parameter being calibrated in the study?
- 322: Ensure that “grounding-zone length” has a hyphen, it is not consistent in the manuscript.
- 325: Should the posterior distribution parameter be $\hat{P}$ instead of P? Similarly, elsewhere: L343, 347, Ext Fig 3 caption
- 340: What are the model and observational uncertainties used here?
- 345: What does “weighted simulation” mean here? It isn’t clear how many simulations are extended to 2100.

Supplement

Make sure the Supplement title is the same as main manuscript.

References

Bett et al. (2026), "*Modelling the evolution of Thwaites Glacier over the 20th century*", EGUsphere [in review]

De Rydt & Naughten (2024), “*Geometric amplification and suppression of ice-shelf basal melt in West Antarctica*”, The Cryosphere

De Rydt (2026) "*Basal sliding and ice rheology in West Antarctica constrained by multi-epoch satellite observations*", Journal of Glaciology [in review]

Goldberg and Holland (2022), "*The Relative Impacts of Initialization and Climate Forcing in Coupled Ice Sheet-Ocean Modeling: Application to Pope, Smith, and Kohler Glaciers*", JGR: Earth Surface

Holland et al. (2023), "*Strong Ocean Melting Feedback During the Recent Retreat of Thwaites Glacier*", Geophysical Research Letters

Mouginot et al. (2014), "*Sustained increase in ice discharge from the Amundsen Sea Embayment, West Antarctica, from 1973 to 2013*", Geophysical Research Letters

Reed et al. (2026), "*Calibration of a coupled ice sheet-ocean model using observations of ice dynamics and basal melt in West Antarctica*" EGUsphere [in review]
